# Supplementary material for: Accelerated SARS-CoV-2 intrahost evolution leading to distinct genotypes during chronic infection
Source: Cell Rep Med. 2023 Jan 27;4(2):100943. doi: 10.1016/j.xcrm.2023.100943 (PMC9906997; doi:10.1016/j.xcrm.2023.100943)
Supplement: Document S1. Tables S1–S4 and Figures S1–S10 [file mmc1.pdf]

Cell Reports Medicine, Volume *4*

Supplemental information

Accelerated SARS-CoV-2 intrahost evolution leading to distinct genotypes during chronic infection

Chrispin Chaguza, Anne M. Hahn, Mary E. Petrone, Shuntai Zhou, David Ferguson, Mallery I. Breban, Kien Pham, Mario A. Peña-Hernández, Christopher Castaldi, Verity Hill, Yale SARS-CoV-2 Genomic Surveillance Initiative, Wade Schulz, Ronald I. Swanstrom, Scott C. Roberts, and Nathan D. Grubaugh

Supplemental information

Accelerated SARS-CoV-2 intrahost evolution leading to distinct genotypes during chronic infection

Chrispin Chaguza, Anne M. Hahn, Mary E. Petrone, Shuntai Zhou, David Ferguson, Mallery I. Breban, Kien Pham, Mario A. Peña-Hernández, Christopher Castaldi, Verity Hill, Yale SARS-CoV-2 Genomic Surveillance Initiative,

Table S1. Summary of chronic SARS-CoV-2 lineage B.1.517 infection samples included in this study. Related to Figure 2.

Sequence ID	GISAID accession	Days since first positive RT-PCR test	RT-PCR Ct value	Virus copies per mL	Infectious virus	Primer ID sequencing	Dominant intrahost genotype	Primers
hCoV-19/USA/CT-Yale-12056/2021	EPI_ISL_10548915	79	20.5	3.01×10^{08}	Yes	Yes	1	NEB V3
hCoV-19/USA/CT-Yale-12057/2021	EPI_ISL_10548916	89	22.1	1.13×10^{08}	Not tested	Not done	1	NEB V3
hCoV-19/USA/CT-Yale-12058/2021	EPI_ISL_10548917	97	15.6	6.04×10^{09}	Yes	Yes	1	NEB V3
hCoV-19/USA/CT-Yale-12059/2021	EPI_ISL_10548918	104	25.2	1.69×10^{07}	No tested	Not done	1	NEB V3
hCoV-19/USA/CT-Yale-12060/2021	EPI_ISL_10548919	135	24.4	2.76×10^{07}	Not tested	Yes	1	NEB V3
hCoV-19/USA/CT-Yale-4087/2021	EPI_ISL_2035047	149	23.2	5.76×10^{07}	Yes	Yes	1	Illumina V3
hCoV-19/USA/CT-Yale-12061/2021	EPI_ISL_10548920	162	25.9	1.10×10^{07}	Not tested	Not done	1	NEB V3
hCoV-19/USA/CT-Yale-12062/2021	EPI_ISL_10548921	184	28.5	2.25×10^{06}	Not tested	Not done	1	NEB V3
hCoV-19/USA/CT-Yale-5581/2021	EPI_ISL_2716246	192	23.5	4.80×10^{07}	Not tested	Yes	1	Illumina V3
hCoV-19/USA/CT-Yale-5673/2021	EPI_ISL_2776212	205	21.5	1.63×10^{08}	Yes	Yes	1	Illumina V3
hCoV-19/USA/CT-Yale-5792/2021	EPI_ISL_2860316	212	29	1.66×10^{06}	Not tested	Yes	1	NEB V3
hCoV-19/USA/CT-Yale-12063/2021	EPI_ISL_10548922	219	27.2	4.98×10^{06}	Not tested	Not done	1	NEB V3
hCoV-19/USA/CT-Yale-6136/2021	EPI_ISL_3133023	233	22.3	1.00×10^{08}	Not tested	Yes	1	NEB V3
hCoV-19/USA/CT-Yale-6819/2021	EPI_ISL_3370176	247	20.9	2.35×10^{08}	Not tested	Yes	1	Illumina V3
hCoV-19/USA/CT-Yale-9391/2021	EPI_ISL_4198270	281	17.6	1.77×10^{09}	Yes	Yes	2	Illumina V3
hCoV-19/USA/CT-Yale-9977/2021	EPI_ISL_4576991	291	23.53	4.71×10^{07}	Not tested	Yes	2	Illumina V3
hCoV-19/USA/CT-Yale-10101R/2021	EPI_ISL_10548912	296	22.11	1.12×10^{08}	Not tested	Not done	1	NEB V3

hCoV-19/USA/CT-Yale-10960/2021	EPI_ISL_10548913	310	29.9	9.54×10^{05}	Yes	Not done	2	NEB V3
hCoV-19/USA/CT-Yale-11558/2021	EPI_ISL_5395558	317	24.6	2.45×10^{07}	Not tested	Yes	2	NEB V3
hCoV-19/USA/CT-Yale-11887/2021	EPI_ISL_5639913	325	29.1	1.56×10^{06}	Yes	Not done	2	NEB V3
hCoV-19/USA/CT-Yale-12124/2021	EPI_ISL_5865553	332	25.4	1.50×10^{07}	Not tested	Yes	2	NEB V3
hCoV-19/USA/CT-Yale-13443/2021	EPI_ISL_7361483	347	28.3	2.54×10^{06}	Yes	Yes	1	IDT V4
hCoV-19/USA/CT-Yale-13444/2021	EPI_ISL_7361527	353	32.5	1.94×10^{05}	Not tested	Not done	1	IDT V4
hCoV-19/USA/CT-Yale-14026/2021	EPI_ISL_7980711	360	26.3	8.64×10^{06}	Not tested	Yes	2	IDT V4.1
hCoV-19/USA/CT-Yale-15439/2021	EPI_ISL_8563219	381	21.2	1.96×10^{08}	Yes	Yes	1	IDT V4
hCoV-19/USA/CT-Yale-15438/2021	EPI_ISL_8563218	394	33.6	9.91×10^{04}	No	Not done	3	IDT V4
hCoV-19/USA/CT-Yale-15437/2021	EPI_ISL_8563217	401	26.1	9.77×10^{06}	Yes	Yes	3	IDT V4
hCoV-19/USA/CT-Yale-17291/2022	EPI_ISL_10815044	446	34.1	7.30×10^{04}	Not tested	Not done	2	IDT V4.1
hCoV-19/USA/CT-Yale-17881/2022	EPI_ISL_11025821	459	30.03	8.81×10^{05}	Not tested	Not done	2	IDT V4.1
hCoV-19/USA/CT-Yale-18086/2022	EPI_ISL_11503909	471	30.9	5.18×10^{05}	No	Not done	2	IDT V4.1

Table S2. Summary of sequenced SARS-CoV-2 genomes analyzed in this study collected from the infected immunocompromised patient. Related to Figures 2-6.

Sequence ID	Sequence data repository	Accession number
hCoV-19/USA/CT-Yale-12056/2021	GISAID	EPI_ISL_10548915
hCoV-19/USA/CT-Yale-12057/2021	GISAID	EPI_ISL_10548916
hCoV-19/USA/CT-Yale-12058/2021	GISAID	EPI_ISL_10548917
hCoV-19/USA/CT-Yale-12059/2021	GISAID	EPI_ISL_10548918
hCoV-19/USA/CT-Yale-12060/2021	GISAID	EPI_ISL_10548919
hCoV-19/USA/CT-Yale-4087/2021	GISAID	EPI_ISL_2035047
hCoV-19/USA/CT-Yale-12061/2021	GISAID	EPI_ISL_10548920
hCoV-19/USA/CT-Yale-12062/2021	GISAID	EPI_ISL_10548921
hCoV-19/USA/CT-Yale-5581/2021	GISAID	EPI_ISL_2716246
hCoV-19/USA/CT-Yale-5673/2021	GISAID	EPI_ISL_2776212
hCoV-19/USA/CT-Yale-5792/2021	GISAID	EPI_ISL_2860316
hCoV-19/USA/CT-Yale-12063/2021	GISAID	EPI_ISL_10548922
hCoV-19/USA/CT-Yale-6136/2021	GISAID	EPI_ISL_3133023
hCoV-19/USA/CT-Yale-6819/2021	GISAID	EPI_ISL_3370176
hCoV-19/USA/CT-Yale-9391/2021	GISAID	EPI_ISL_4198270
hCoV-19/USA/CT-Yale-9977/2021	GISAID	EPI_ISL_4576991
hCoV-19/USA/CT-Yale-10101R/2021	GISAID	EPI_ISL_10548912
hCoV-19/USA/CT-Yale-10960/2021	GISAID	EPI_ISL_10548913

hCoV-19/USA/CT-Yale-11558/2021	GISAID	EPI_ISL_5395558
hCoV-19/USA/CT-Yale-11887/2021	GISAID	EPI_ISL_5639913
hCoV-19/USA/CT-Yale-12124/2021	GISAID	EPI_ISL_5865553
hCoV-19/USA/CT-Yale-13443/2021	GISAID	EPI_ISL_7361483
hCoV-19/USA/CT-Yale-13444/2021	GISAID	EPI_ISL_7361527
hCoV-19/USA/CT-Yale-14026/2021	GISAID	EPI_ISL_7980711
hCoV-19/USA/CT-Yale-15439/2021	GISAID	EPI_ISL_8563219
hCoV-19/USA/CT-Yale-15438/2021	GISAID	EPI_ISL_8563218
hCoV-19/USA/CT-Yale-15437/2021	GISAID	EPI_ISL_8563217
hCoV-19/USA/CT-Yale-17291/2022	GISAID	EPI_ISL_10815044
hCoV-19/USA/CT-Yale-17881/2022	GISAID	EPI_ISL_11025821
hCoV-19/USA/CT-Yale-18086/2022	GISAID	EPI_ISL_11503909
hCoV-19/USA/CT-Yale-12056/2021	Sequence Read Archive	SRR23085675
hCoV-19/USA/CT-Yale-12057/2021	Sequence Read Archive	SRR23085674
hCoV-19/USA/CT-Yale-12058/2021	Sequence Read Archive	SRR23085663
hCoV-19/USA/CT-Yale-12059/2021	Sequence Read Archive	SRR23085652
hCoV-19/USA/CT-Yale-12060/2021	Sequence Read Archive	SRR23085651
hCoV-19/USA/CT-Yale-4087/2021	Sequence Read Archive	SRR23085650
hCoV-19/USA/CT-Yale-12061/2021	Sequence Read Archive	SRR23085649
hCoV-19/USA/CT-Yale-12062/2021	Sequence Read Archive	SRR23085648

hCoV-19/USA/CT-Yale-5581/2021	Sequence Read Archive	SRR23085647
hCoV-19/USA/CT-Yale-5673/2021	Sequence Read Archive	SRR23085646
hCoV-19/USA/CT-Yale-5792/2021	Sequence Read Archive	SRR23085673
hCoV-19/USA/CT-Yale-12063/2021	Sequence Read Archive	SRR23085672
hCoV-19/USA/CT-Yale-6136/2021	Sequence Read Archive	SRR23085671
hCoV-19/USA/CT-Yale-6819/2021	Sequence Read Archive	SRR23085670
hCoV-19/USA/CT-Yale-9391/2021	Sequence Read Archive	SRR23085669
hCoV-19/USA/CT-Yale-9977/2021	Sequence Read Archive	SRR23085668
hCoV-19/USA/CT-Yale-10101R/2021	Sequence Read Archive	SRR23085667
hCoV-19/USA/CT-Yale-10960/2021	Sequence Read Archive	SRR23085666
hCoV-19/USA/CT-Yale-11558/2021	Sequence Read Archive	SRR23085665
hCoV-19/USA/CT-Yale-11887/2021	Sequence Read Archive	SRR23085664
hCoV-19/USA/CT-Yale-12124/2021	Sequence Read Archive	SRR23085662
hCoV-19/USA/CT-Yale-13443/2021	Sequence Read Archive	SRR23085661
hCoV-19/USA/CT-Yale-13444/2021	Sequence Read Archive	SRR23085660
hCoV-19/USA/CT-Yale-14026/2021	Sequence Read Archive	SRR23085659
hCoV-19/USA/CT-Yale-15439/2021	Sequence Read Archive	SRR23085658
hCoV-19/USA/CT-Yale-15438/2021	Sequence Read Archive	SRR23085657
hCoV-19/USA/CT-Yale-15437/2021	Sequence Read Archive	SRR23085656
hCoV-19/USA/CT-Yale-17291/2022	Sequence Read Archive	SRR23085655

hCoV-19/USA/CT-Yale-17881/2022	Sequence Read Archive	SRR23085654
hCoV-19/USA/CT-Yale-18086/2022	Sequence Read Archive	SRR23085653

Table S3. Nucleotide substitution or mutation rates of the chronic infection samples and other SARS-CoV-2 variants. Related to Figure 3.

Strain	Mutations or substitutions per year (s/y)			Mutations or substitutions per site year (s/s/y)			Regression R^2
	Estimate	Lower 95% CI	Upper 95% CI	Estimate	Lower 95% CI	Upper 95% CI	
Chronic infection	35.55	31.56	39.54	1.21×10^{-03}	1.07×10^{-03}	1.34×10^{-03}	0.92
Theta	24.74	22.23	27.26	8.41×10^{-04}	7.56×10^{-04}	9.27×10^{-04}	0.67
All lineages	17.16	16.36	17.96	5.83×10^{-04}	5.56×10^{-04}	6.11×10^{-04}	0.41
B.1.517 (other)	16.93	13.46	20.41	5.76×10^{-04}	4.58×10^{-04}	6.94×10^{-04}	0.51
Lambda	15.30	13.96	16.64	5.20×10^{-04}	4.75×10^{-04}	5.66×10^{-04}	0.59
Omicron	12.26	9.68	14.84	4.17×10^{-04}	3.29×10^{-04}	5.05×10^{-04}	0.24
Mu	11.05	9.62	12.49	3.76×10^{-04}	3.27×10^{-04}	4.25×10^{-04}	0.35
Gamma	10.62	9.27	11.97	3.61×10^{-04}	3.15×10^{-04}	4.07×10^{-04}	0.34
Zeta	10.26	8.56	11.96	3.49×10^{-04}	2.91×10^{-04}	4.07×10^{-04}	0.27
Iota	9.31	7.45	11.17	3.17×10^{-04}	2.53×10^{-04}	3.80×10^{-04}	0.20
Epsilon	8.98	7.56	10.41	3.05×10^{-04}	2.57×10^{-04}	3.54×10^{-04}	0.30
Delta	8.96	7.64	10.27	3.05×10^{-04}	2.60×10^{-04}	3.49×10^{-04}	0.24
Beta	8.93	7.46	10.39	3.04×10^{-04}	2.54×10^{-04}	3.53×10^{-04}	0.27
Alpha	8.91	7.83	9.99	3.03×10^{-04}	2.66×10^{-04}	3.40×10^{-04}	0.29

Table S4. cDNA and forward primer sequences for Multiplexed Primer ID (MPID) MiSeq library preparation for the SARS-CoV-2 S gene and nsp12. Related to Figure 6.

**Mix A**		
**Region**	**cDNA primer with Primer ID**	**First Round PCR Forward**
S-N-3	GTGACTGGAGTTCAGACGTGTGCTCTTCCGATCTNNNNNNNNNNNNCAGTAGTACCAAAAATCCAGCCTCT	GCCTCCCTCGCGCCATCAGAGATGTGTATAAGAGACAGNNNNAAGGGGTACTGCTGTTATGT
S-RBD-1	GTGACTGGAGTTCAGACGTGTGCTCTTCCGATCTNNNNNNNNNNNNCAGTAGTTGCTGATTCTCTTCCTGT	GCCTCCCTCGCGCCATCAGAGATGTGTATAAGAGACAGNNNNAATTTAGTGCGTGATCTCCCT
S-RBD-10	GTGACTGGAGTTCAGACGTGTGCTCTTCCGATCTNNNNNNNNNNNNCAGTTGCTGGTGCATGTAGAAGTT	GCCTCCCTCGCGCCATCAGAGATGTGTATAAGAGACAGNNNNTTCCGCATCATTTTCCACTTT
S2-9	GTGACTGGAGTTCAGACGTGTGCTCTTCCGATCTNNNNNNNNNNNNCAGTGGCAATGATGGATTGACTAGC	GCCTCCCTCGCGCCATCAGAGATGTGTATAAGAGACAGNNNNGGCACAGGTGTTCTTACTGA
NSP12-5	GTGACTGGAGTTCAGACGTGTGCTCTTCCGATCTNNNNNNNNNNNNCAGTGTGCCAACCACCATAGAATTT	GCCTCCCTCGCGCCATCAGAGATGTGTATAAGAGACAGNNNNCTTCTTCTTTGCTCAGGATGG
**Mix B**		
**Region**	**cDNA primer with Primer ID**	**First Round PCR Forward**
S1-15	GTGACTGGAGTTCAGACGTGTGCTCTTCCGATCTNNNNNNNNNNNNCAGTGTTCTAAAGCCGAAAAACCCT	GCCTCCCTCGCGCCATCAGAGATGTGTATAAGAGACAGNNNNTTGATAACCCTGTCCTACCA
S-9	GTGACTGGAGTTCAGACGTGTGCTCTTCCGATCTNNNNNNNNNNNNCAGTAGCTATAACGCAGCCTGTAA	GCCTCCCTCGCGCCATCAGAGATGTGTATAAGAGACAGNNNNTGTACGTTGAAATCCTTCACTG

S-gap-4	GTGACTGGAGTTCAGACGTGTGCTCTTCCGATCTNNNNNNNNNNNNCAGTCAGGGACTTCTGTGCAGTTA	GCCTCCCTCGCGCCATCAGAGATGTGTATAAGAGACAGNNNNCCGGTAGCACACCTTGTAAT
S2-3	GTGACTGGAGTTCAGACGTGTGCTCTTCCGATCTNNNNNNNNNNNNCAGTGACCTCTTGCTTGGTTTTGA	GCCTCCCTCGCGCCATCAGAGATGTGTATAAGAGACAGNNNNTGCAGGTATATGCGCTAGTT

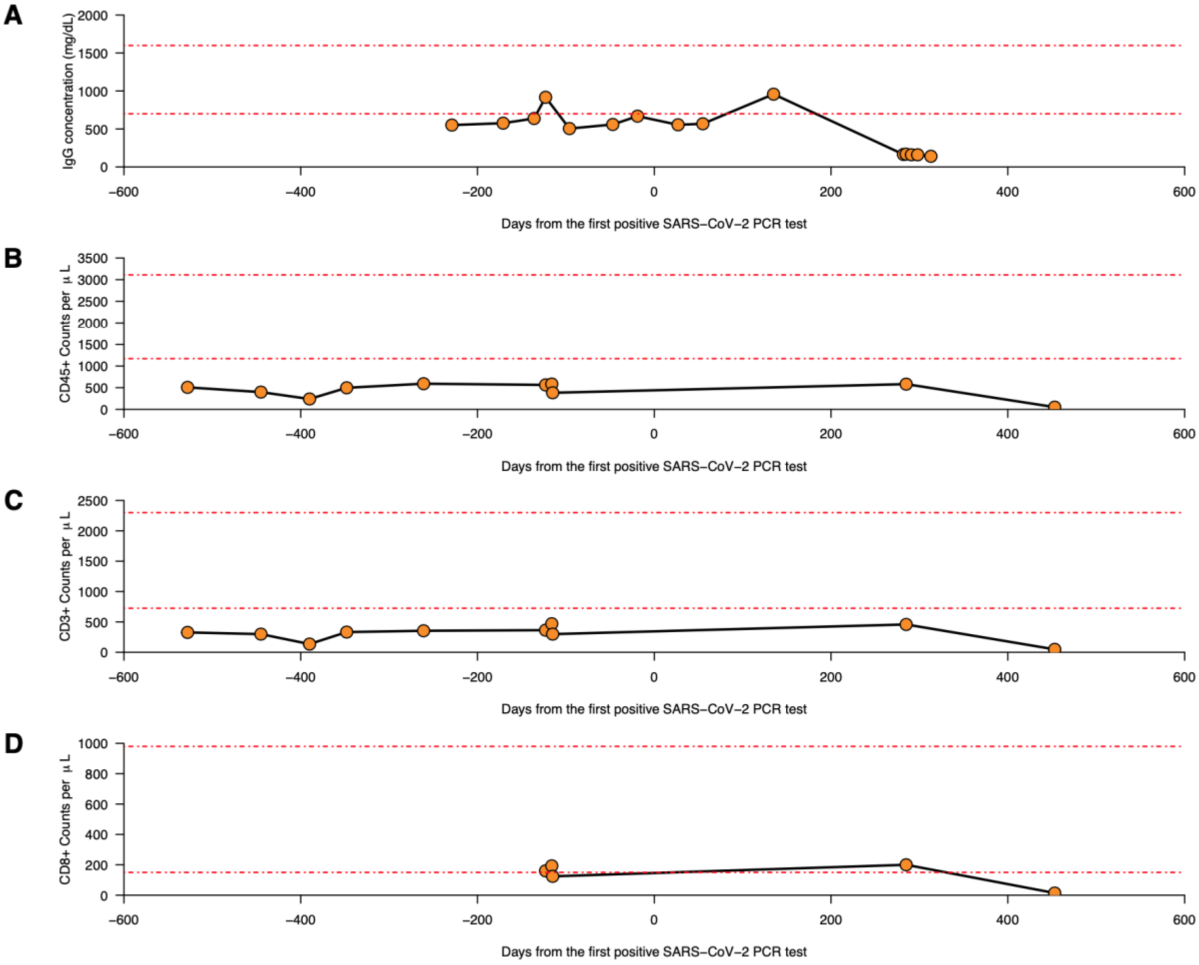

Figure S1: Key adaptive immune parameters as obtained from longitudinal chart reviews. Related to Figure 2. **(A)** The red dotted lines show the serum concentration of Immunoglobulin G (IgG) (reference range from 700-1600 mg/dL). **(B)** Cell counts per µl blood for $CD45^{+}$ lymphocytes (reference range from 1170-3110/µl are shown by the red dotted lines) **(C)**, $CD3^{+}$ T cells (reference range from 725-2300/µl are indicated by the red dotted lines) and **(D)** $CD8^{+}$ cytotoxic T cells (reference range from 150-980/µl are shown by the red dotted lines).

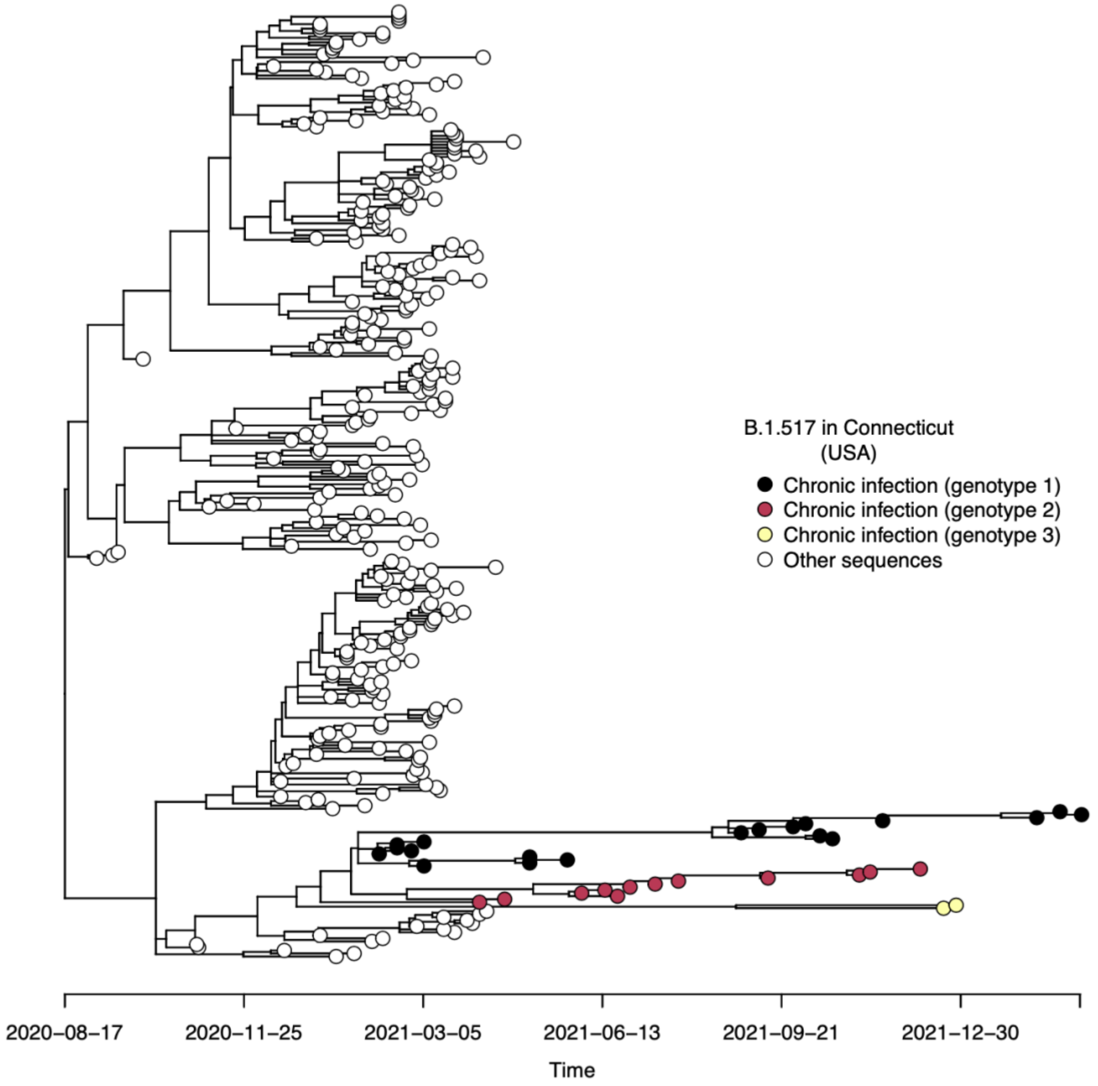

Figure S2: Time-resolved phylogenetic tree showing genetic relatedness of the B.1.517 SARS-CoV-2 strains from the chronic infection and contextual genomes from Connecticut, USA. Related to Figures 1 and 2. The B.1.517 sequences associated with the chronic infection formed a separate monophyletic clade from the rest of the B.1.517 sequences from Connecticut, USA indicating that there were no detectable onward transmission events from the patient with the chronic B.1.517 infection into the wider population in Connecticut, USA.

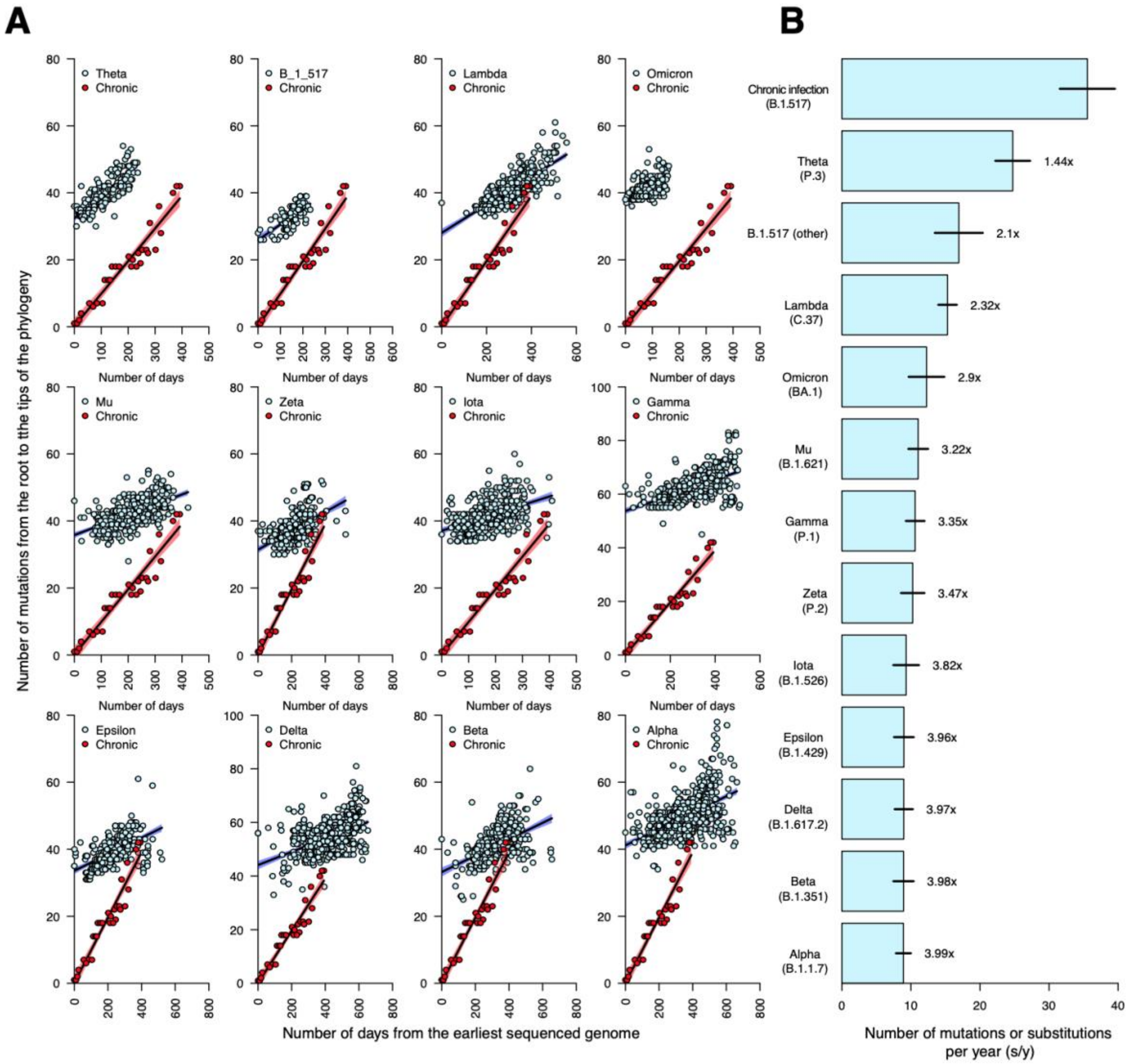

Figure S3: Genomes from B.1.517 chronic infection samples accelerated genetic divergence or higher mutation rates than other SAR-CoV-2 variants of interest and concern. **Related to Figure 3. (A)** Scatter plots show the relationship between phylogenetic root-to-tip distances, expressed as the number of mutations or nucleotide substitutions per site, and time as the number of days from the first sampled genome. The data points associated with the chronic infection are colored in red while those representing other variants are colored in sky blue. The lines and shaded bands surrounding them represent the linear regression models fitted to the data points for the chronic infection data and other variants. **(B)** Bar graph showing the average mutation rates expressed as the number of nucleotide substitutions per site per year (s/s/y) for the chronic infection samples and other variants based on the regression coefficients (β) generated from the plots in panel A. Specific values for the mutation rate are shown in **Table S3**.

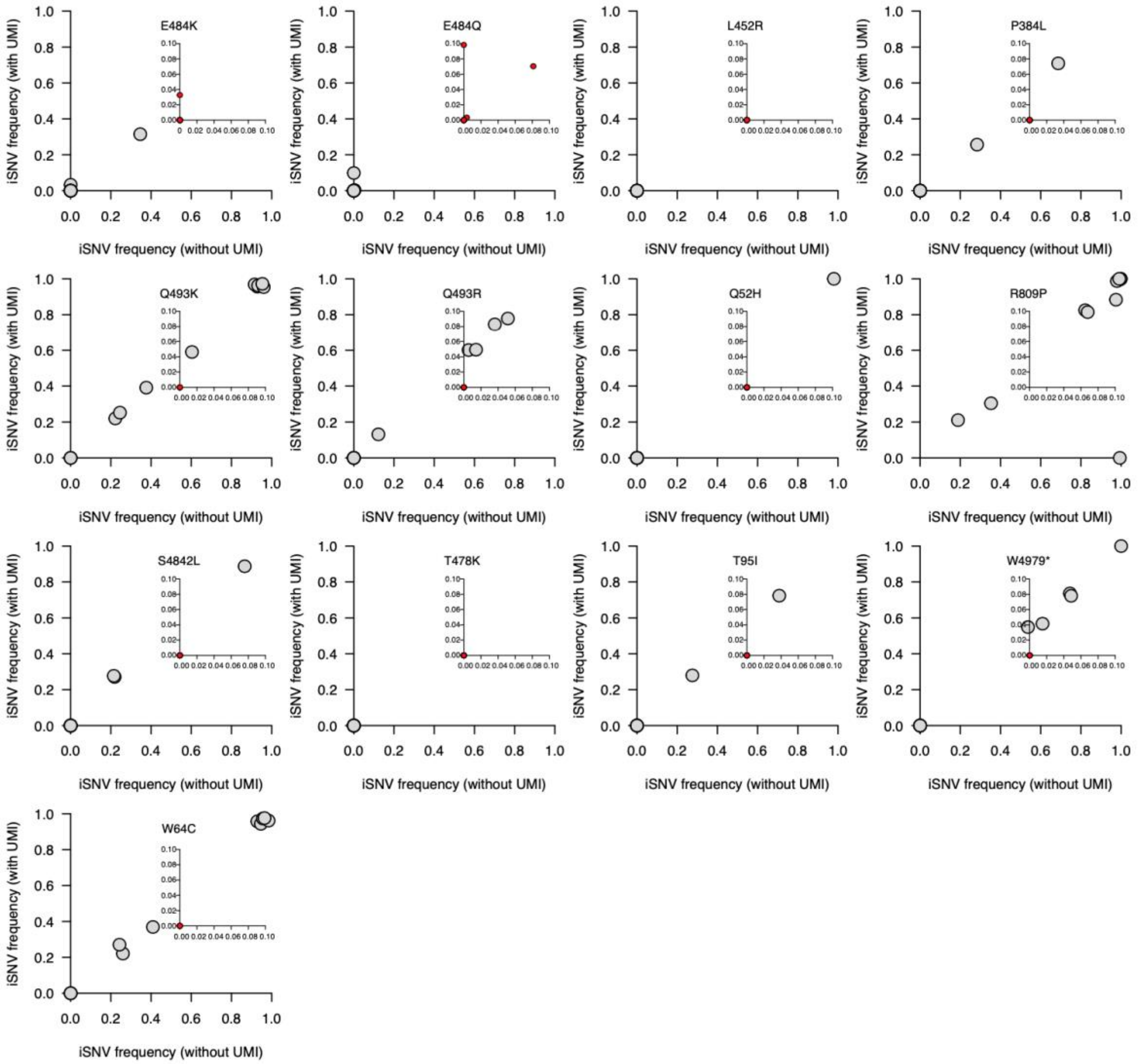

Figure S4: High concordance of the frequency of single nucleotide variants of the spike glycoprotein mutations between samples deep sequenced with and without unique molecular identifiers (UMI). Related to Figure 6. The genomic data without the UMIs were deep-sequenced using routine amplicon-based sequencing protocol using ARTIC V3, V4, and V4.1 primers for amplicon generation using 2×150 bp paired-end reads on an Illumina NovaSeq. Samples selected for deep-sequencing with the UMIs based on the Primer ID next-generation sequencing protocol were sequenced using MiSeq 2×300 bp paired-end reads. The insets show variants, colored in red, with frequencies between 0 to 0.1.

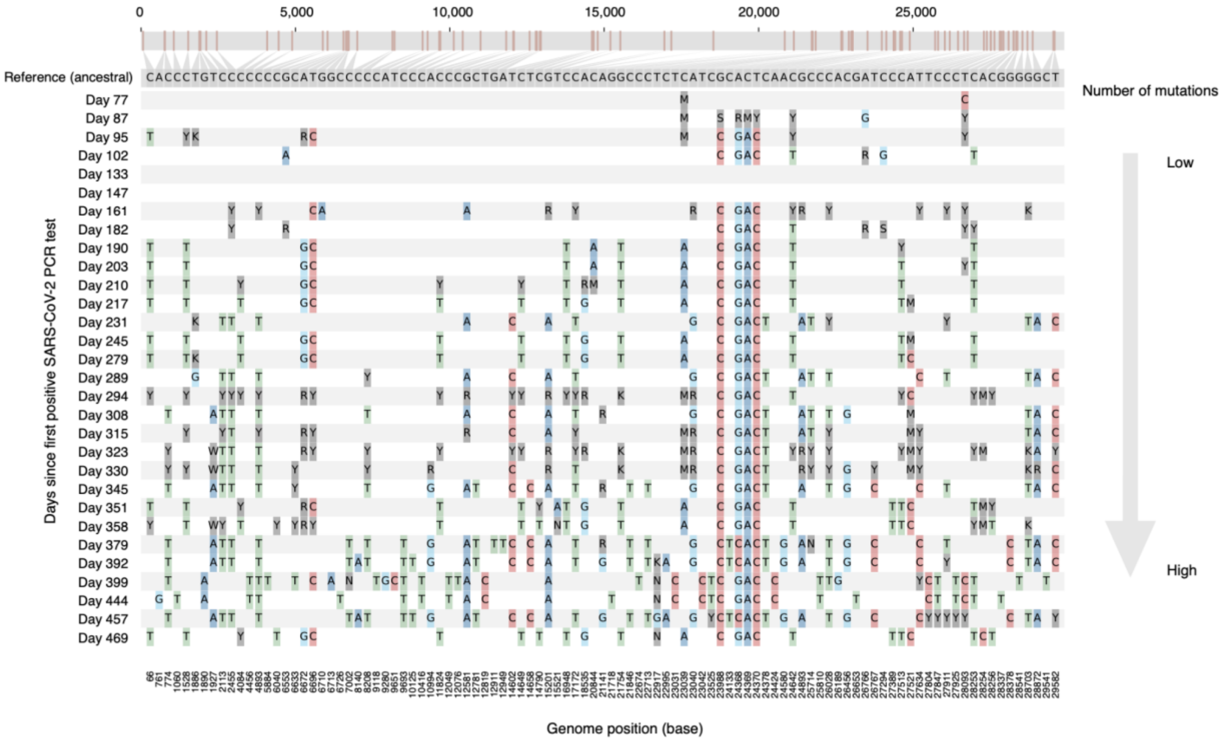

Figure S5: Nucleotide variation in B.1.517 SARS-CoV-2 genomes longitudinally sampled from a chronically infected patient. Related to Figures 4-5. Consensus sequences for each genome were compared against a reference genome, a reconstructed ancestral sequence for all the chronic infection genomes.

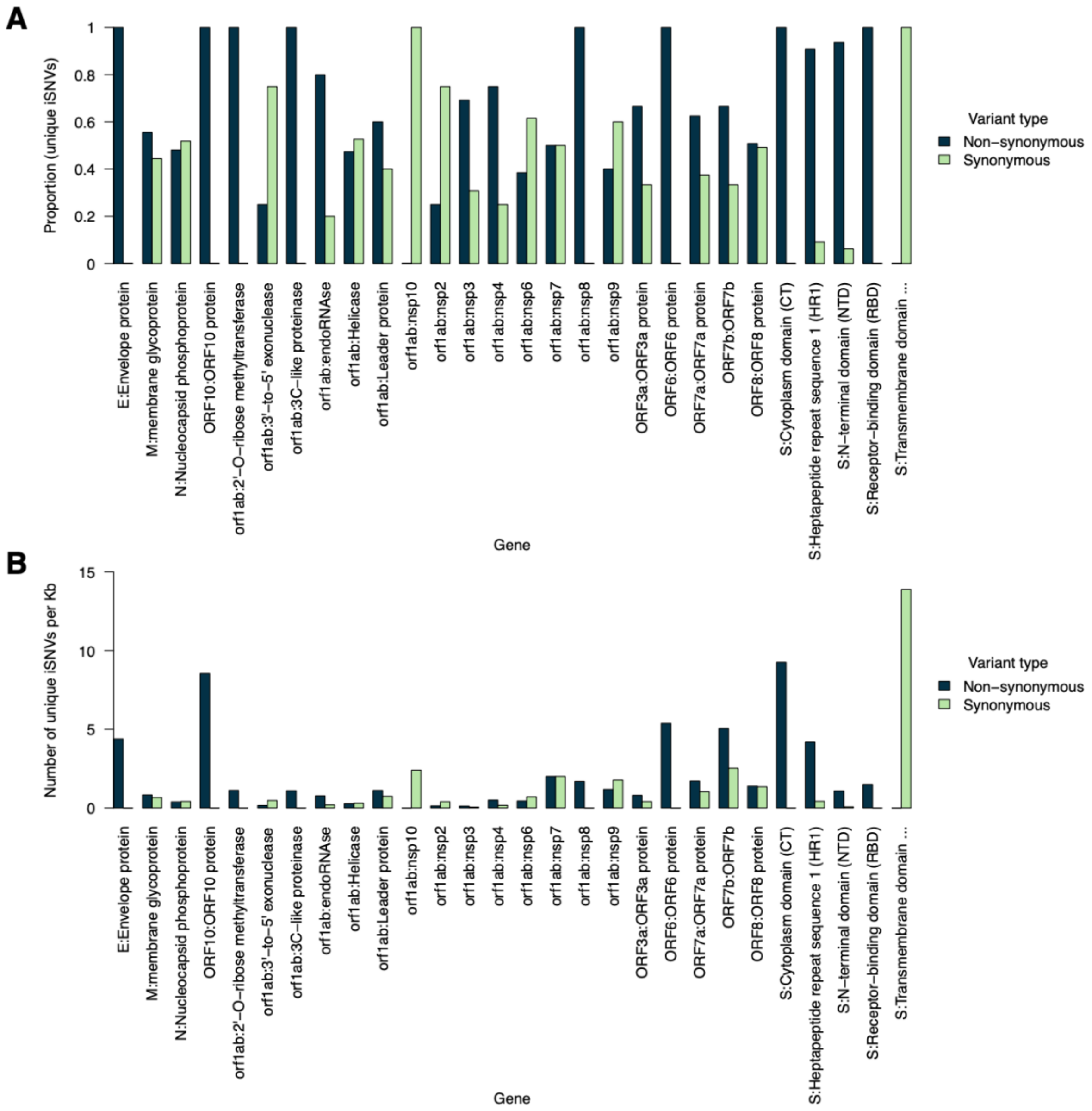

Figure S6: Distribution of iSNVs or mutations detected in the deep-sequenced longitudinal chronic infection samples. Related to Figures 4-5. The y-axis shows the number of iSNVs per kilobase for different SARS-CoV-2 genomic features based on the sequence annotations in the ancestral SARS-CoV-2 reference genome (GenBank accession: NC_045512.2). The bars in the graph are colored by the variant or mutation type. Additional information is provided in **Data S1**.

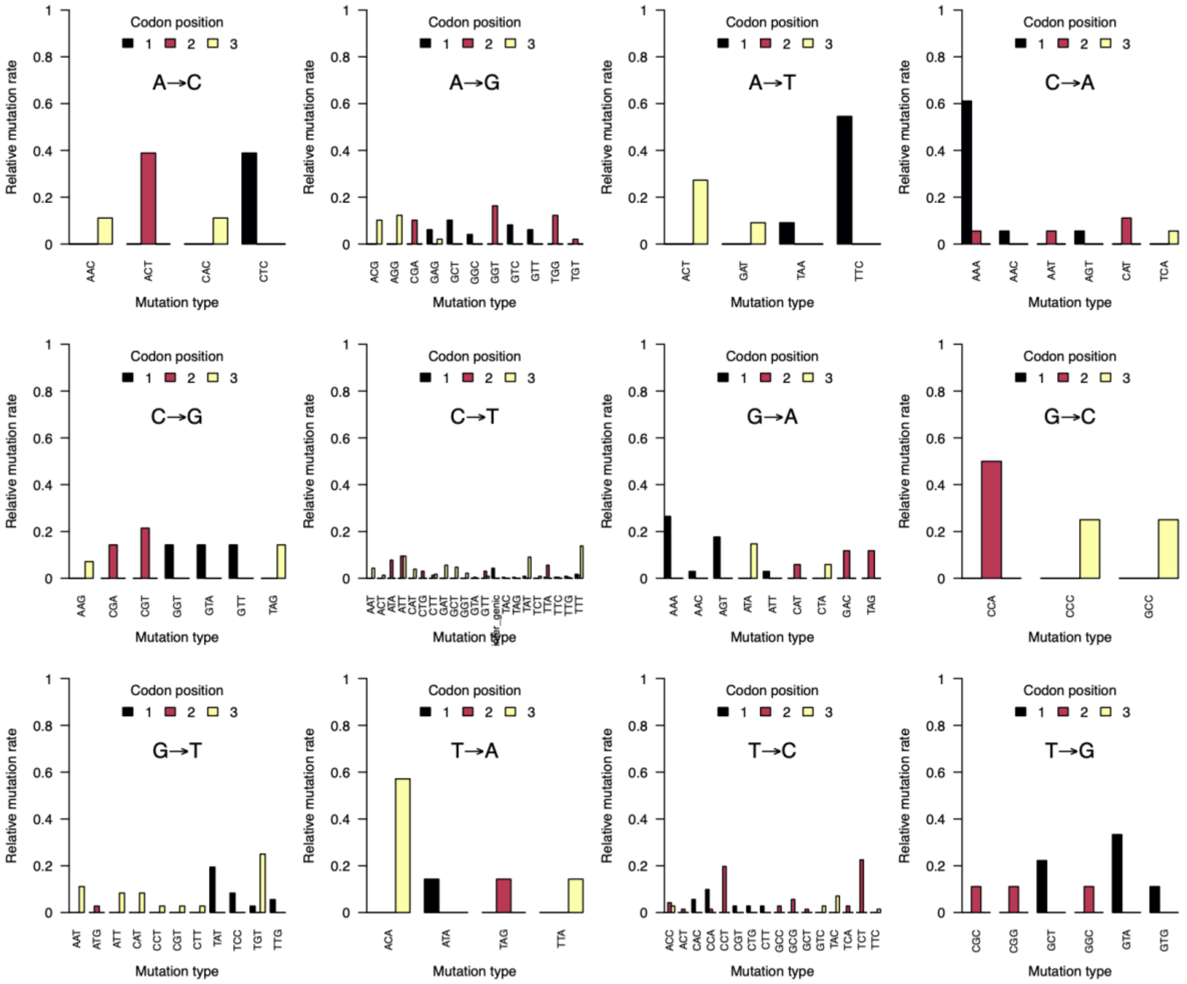

Figure S7: Mutation spectra of identified twelve trinucleotides during the B.1.517 chronic infection stratified by codon position. Related to Figures 4-5. Additional information is provided in **Data S1**.

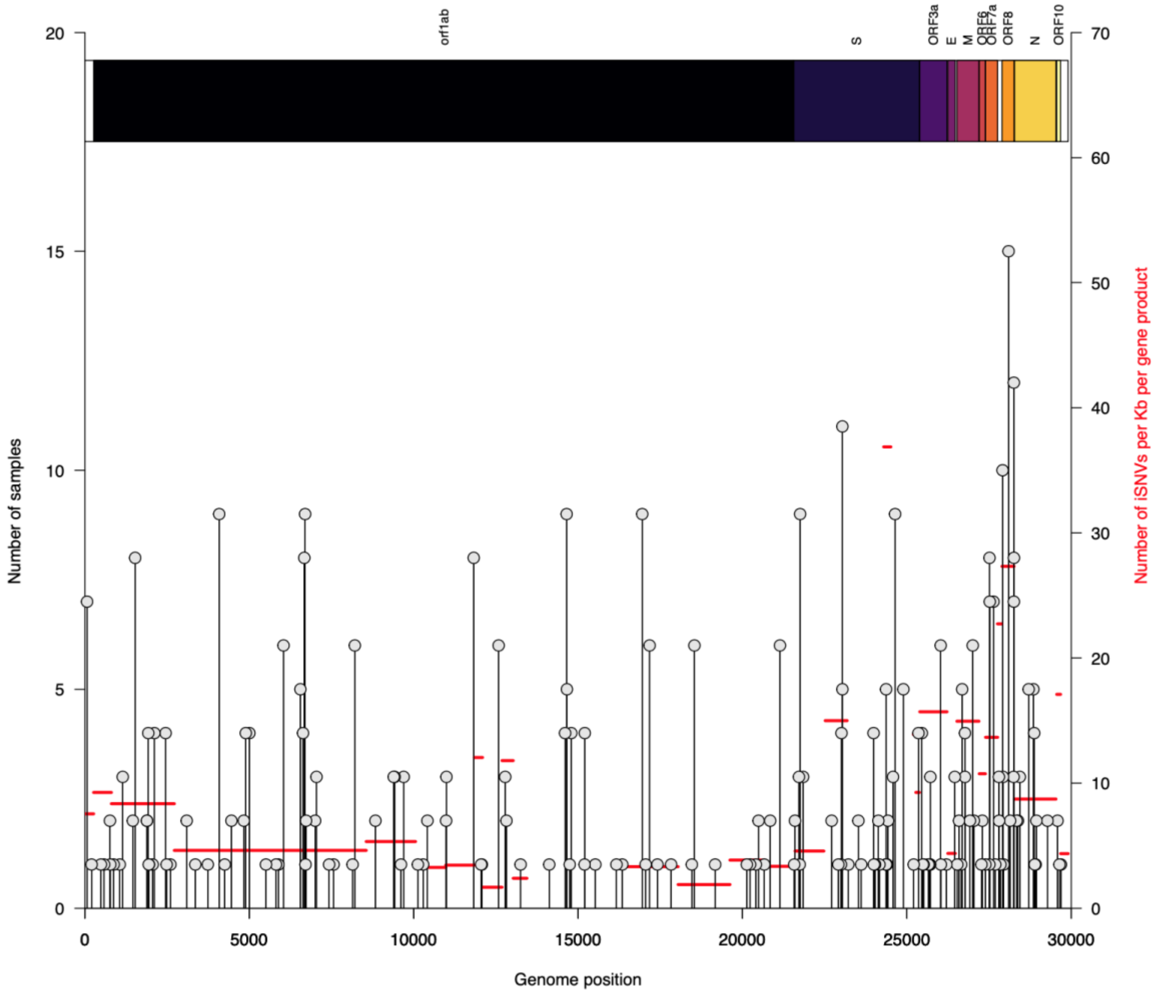

Figure S8: Distribution of iSNVs or mutations detected in the deep-sequenced longitudinal chronic infection samples. Related to Figures 4-5. Graph showing the number of samples containing each unique iSNV and its position in the ancestral SARS-CoV-2 reference genome. The y-axis labels on the right side of the plot show the number of iSNVs per gene and their position in the SARS-CoV-2 genomes stratified based on the sequence feature annotations in the ancestral SARS-CoV-2 reference genome (GenBank accession: NC_045512.2). Additional information is provided in **Data S1**.

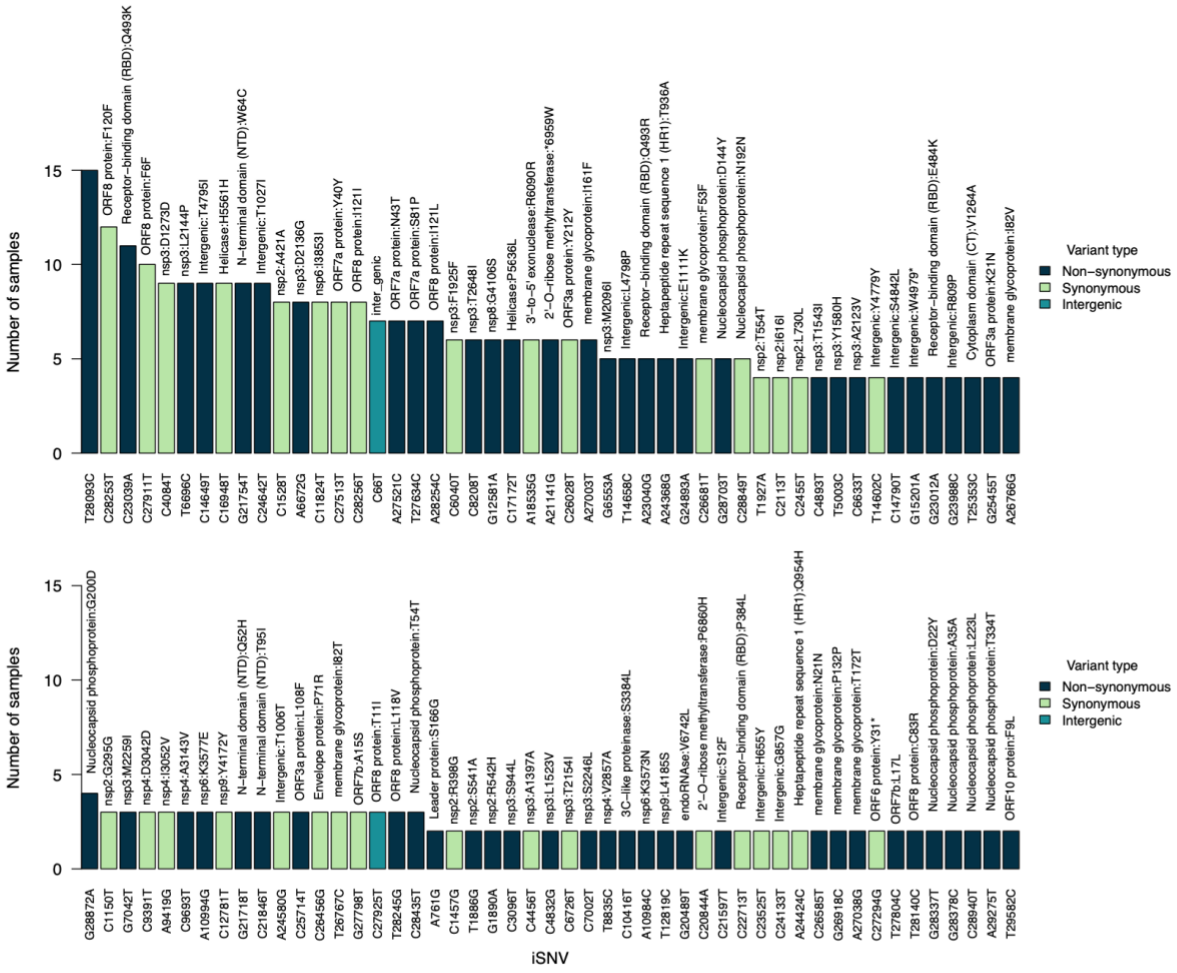

Figure S9: Distribution of iSNVs or mutations detected in the deep-sequenced longitudinal chronic infection samples. Related to Figures 4-5. The x-axis labels represent iSNVs corresponding to specific nucleotide substitutions and positions in the genome. The labels above the bars show the specific amino acid changes and their specific position in the SARS-CoV-2 genomes stratified based on the sequence feature annotations in the ancestral reference genome (GenBank accession: NC_045512.2). All the iSNVs are colored by the variant or mutation type. Additional information is provided in **Data S1**.

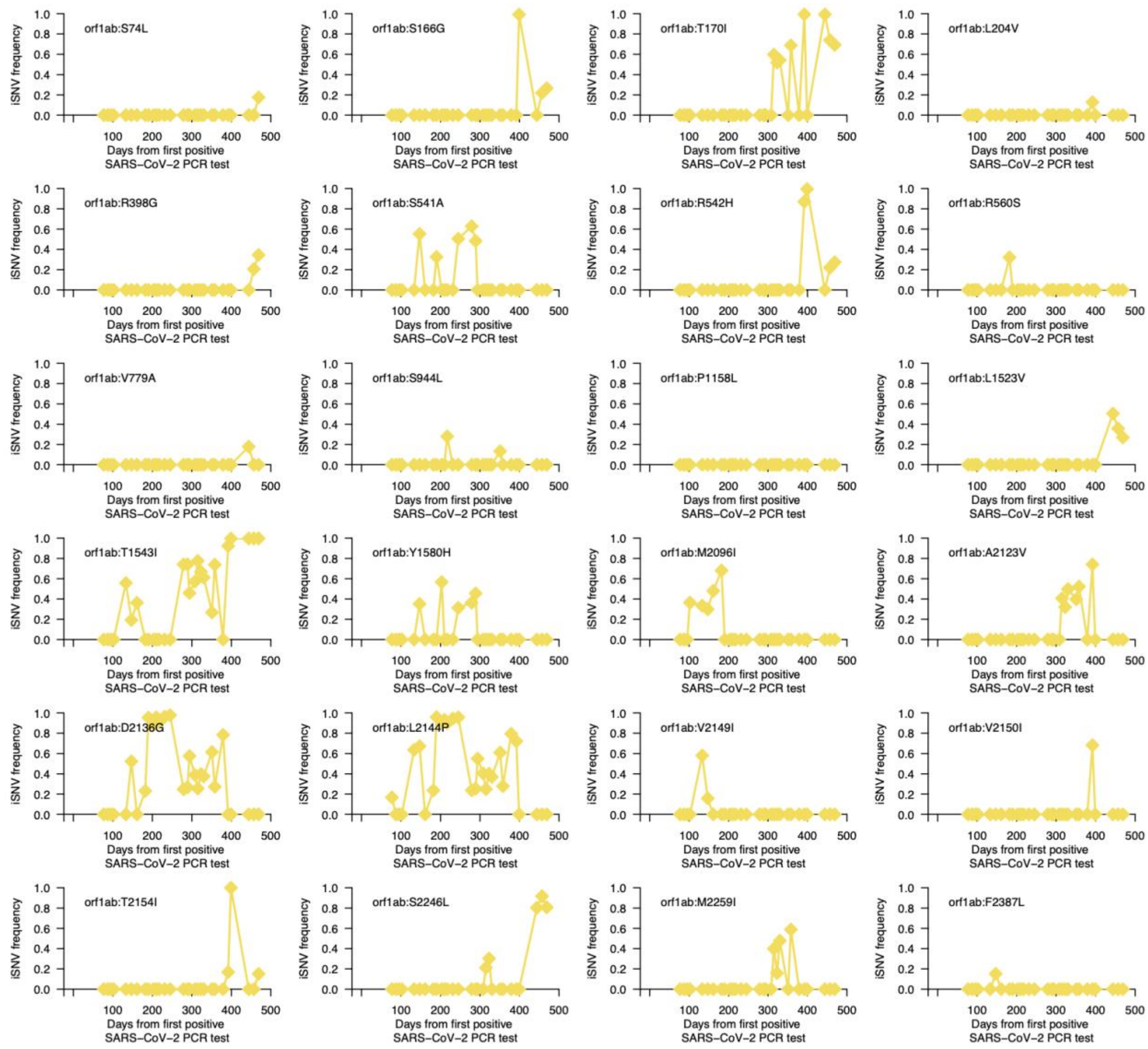

Figure S10: Intrahost non-synonymous mutation dynamics during chronic B.1.517 SARS-CoV-2 infection. Related to Figure 6. The graphs show temporal frequencies of non-synonymous iSNVs or mutations identified in the entire genome. Additional information for all the identified mutations (intergenic, synonymous, and non-synonymous) are provided in **Data S1**.

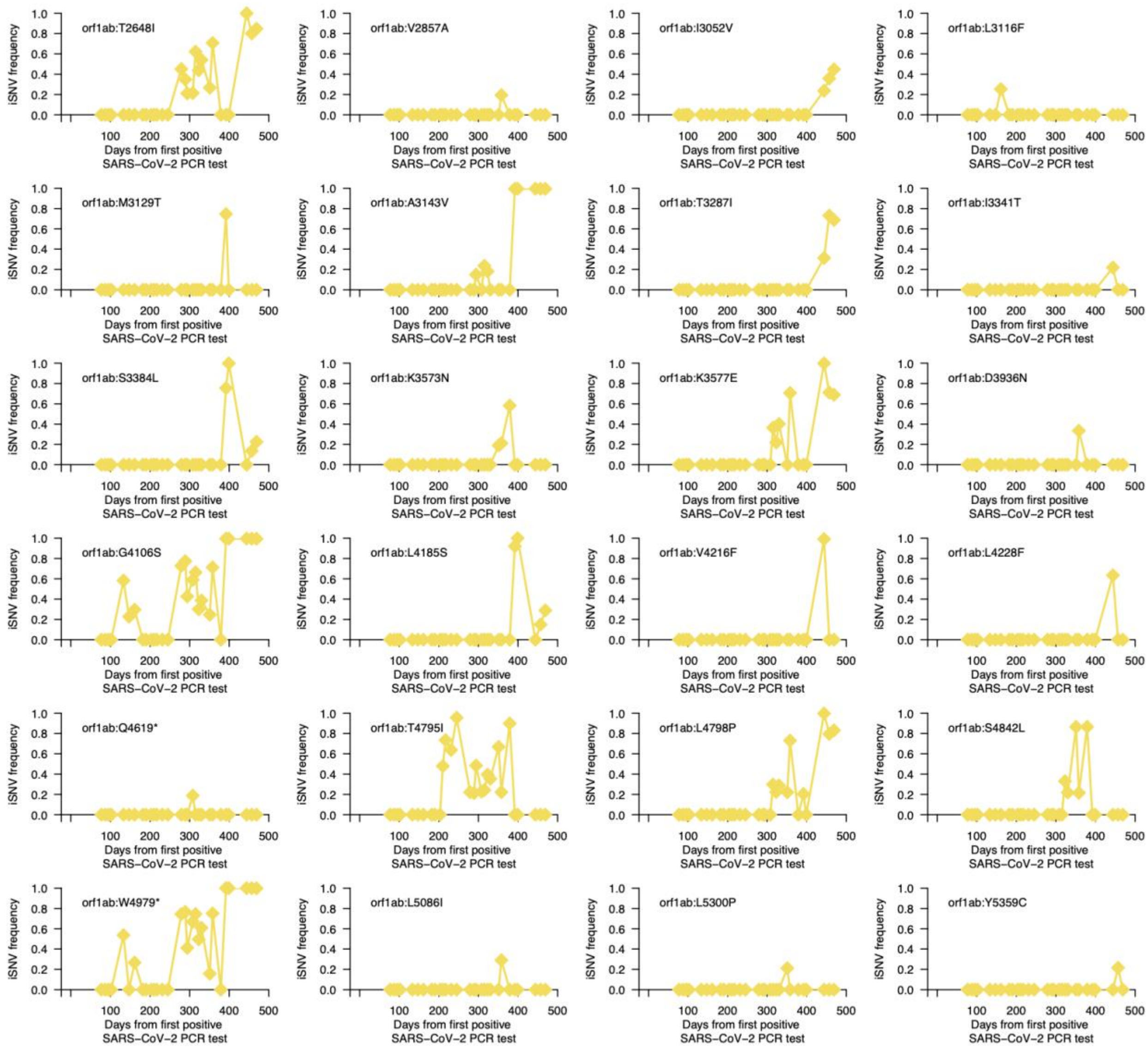

Figure S10 (continued): Intrahost non-synonymous mutation dynamics during chronic B.1.517 SARS-CoV-2 infection. Related to Figure 6. The graphs show temporal frequencies of non-synonymous iSNVs or mutations identified in the entire genome. Additional information for all the identified mutations (intergenic, synonymous, and non-synonymous) are provided in **Data S1**.

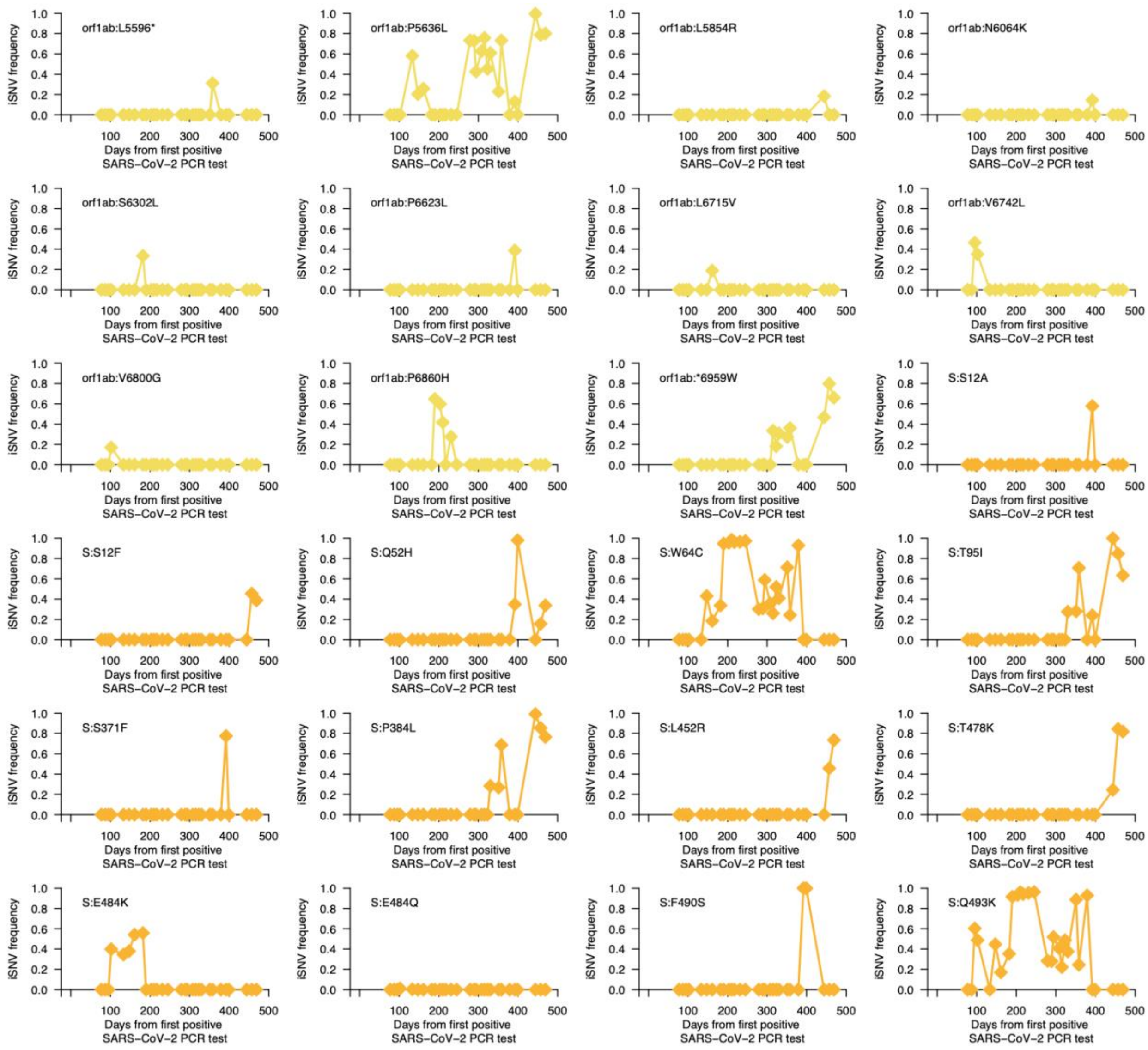

Figure S10 (continued): Intrahost non-synonymous mutation dynamics during chronic B.1.517 SARS-CoV-2 infection. Related to Figure 6. The graphs show temporal frequencies of non-synonymous iSNVs or mutations identified in the entire genome. Additional information for all the identified mutations (intergenic, synonymous, and non-synonymous) are provided in **Data S1**.

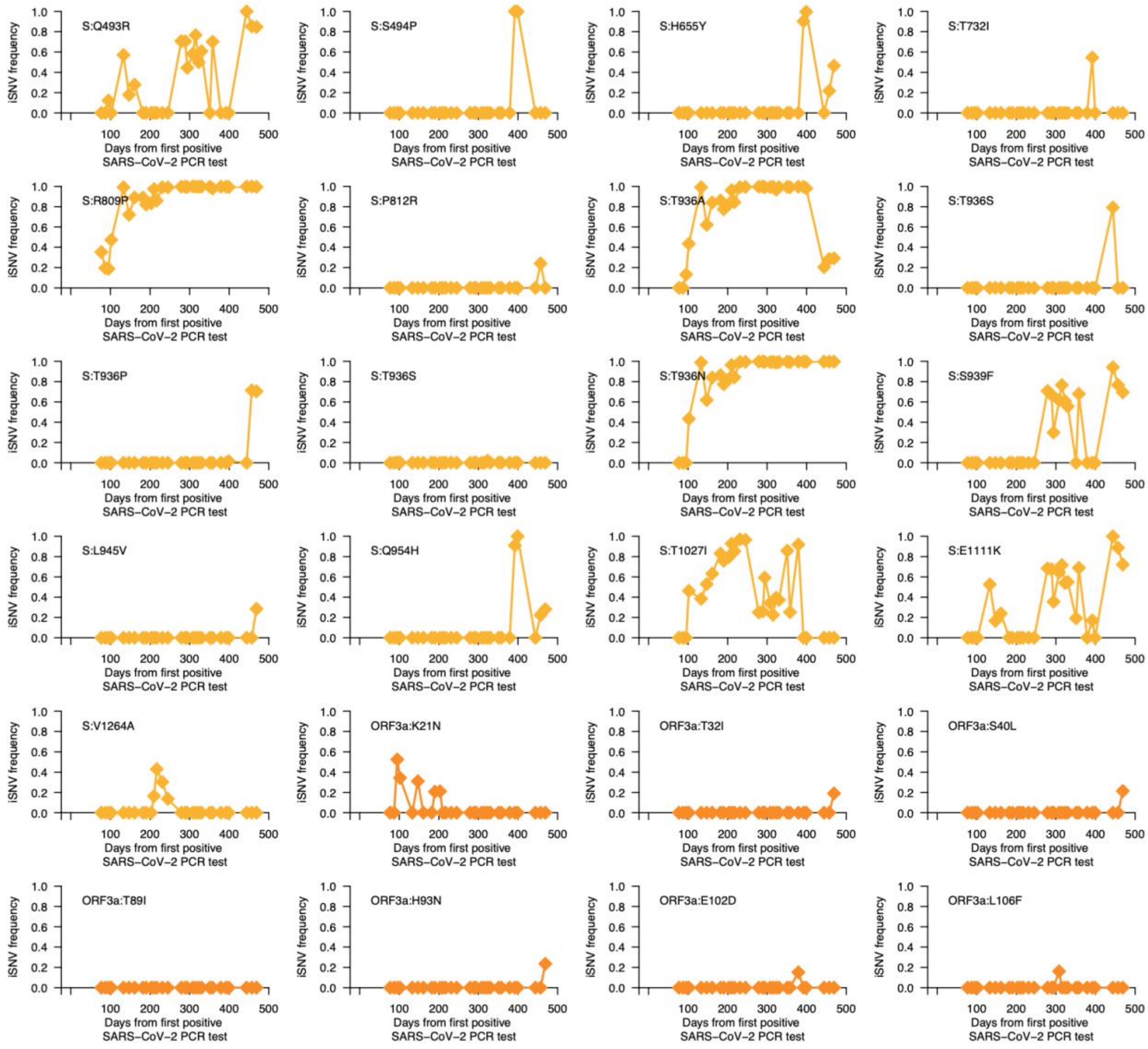

Figure S10 (continued): Intrahost non-synonymous mutation dynamics during chronic B.1.517 SARS-CoV-2 infection. Related to Figure 6. The graphs show temporal frequencies of non-synonymous iSNVs or mutations identified in the entire genome. Additional information for all the identified mutations (intergenic, synonymous, and non-synonymous) are provided in **Data S1**.

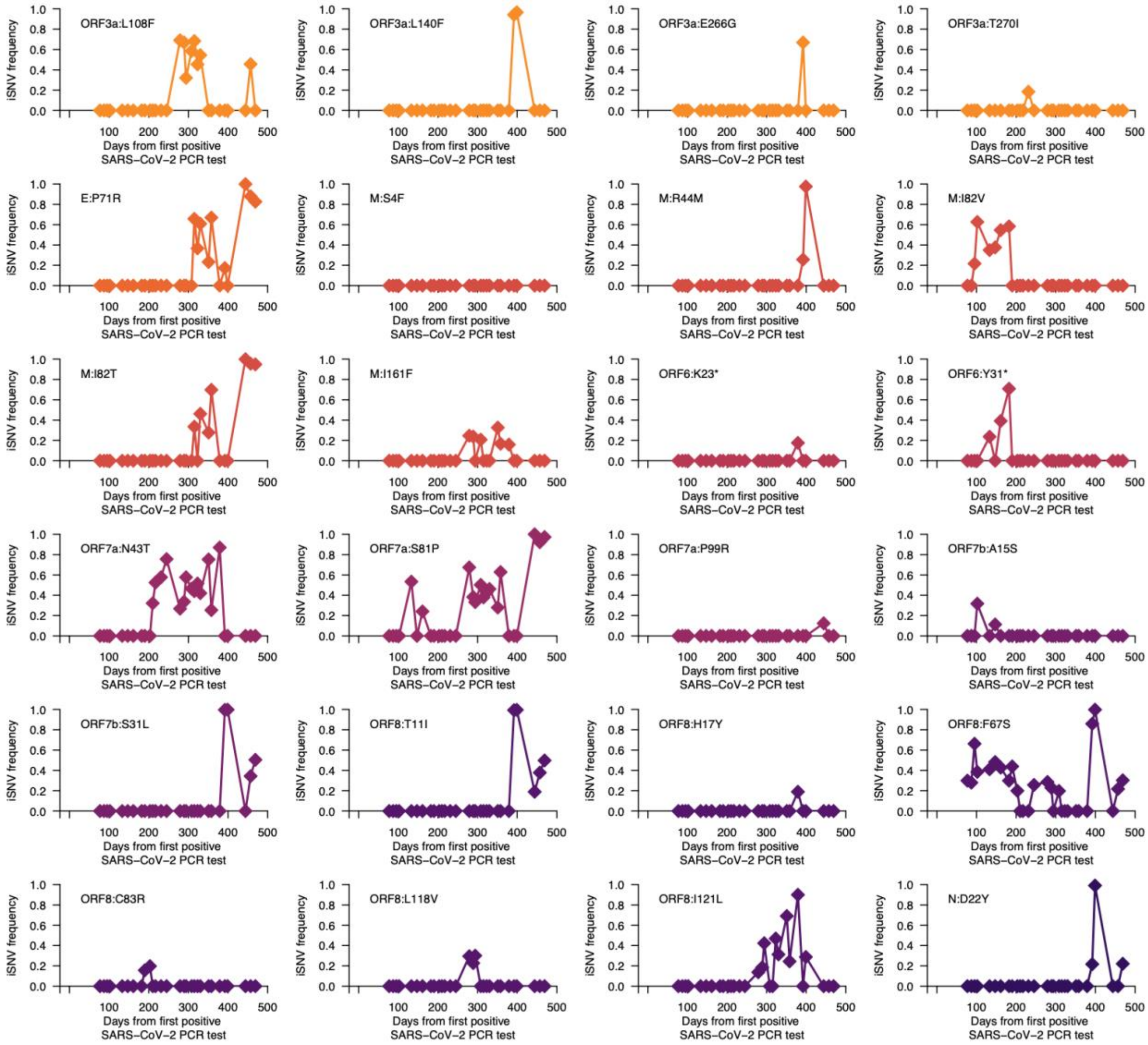

Figure S10 (continued): Intrahost non-synonymous mutation dynamics during chronic B.1.517 SARS-CoV-2 infection. Related to Figure 6. The graphs show temporal frequencies of non-synonymous iSNVs or mutations identified in the entire genome. Additional information for all the identified mutations (intergenic, synonymous, and non-synonymous) are provided in **Data S1**.

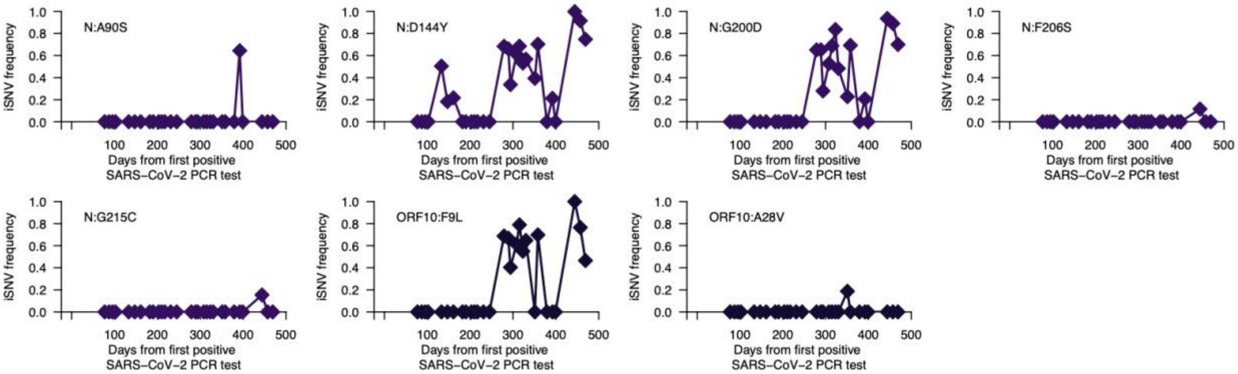

Figure S10 (continued): Intrahost non-synonymous mutation dynamics during chronic B.1.517 SARS-CoV-2 infection. Related to Figure 6. The graphs show temporal frequencies of non-synonymous iSNVs or mutations identified in the entire genome. Additional information for all the identified mutations (intergenic, synonymous, and non-synonymous) are provided in **Data S1**.
