# Supplementary material for: Accelerated SARS-CoV-2 intrahost evolution leading to distinct genotypes during chronic infection
Source: Cell Rep Med. 2023 Jan 27;4(2):100943. doi: 10.1016/j.xcrm.2023.100943 (PMC9906997; doi:10.1016/j.xcrm.2023.100943)
Supplement: Document S2. Article plus supplemental information [file mmc3.pdf]

Cell Reports
Medicine

Article

Accelerated SARS-CoV-2 intrahost evolution leading to distinct genotypes during chronic infection

Graphical abstract

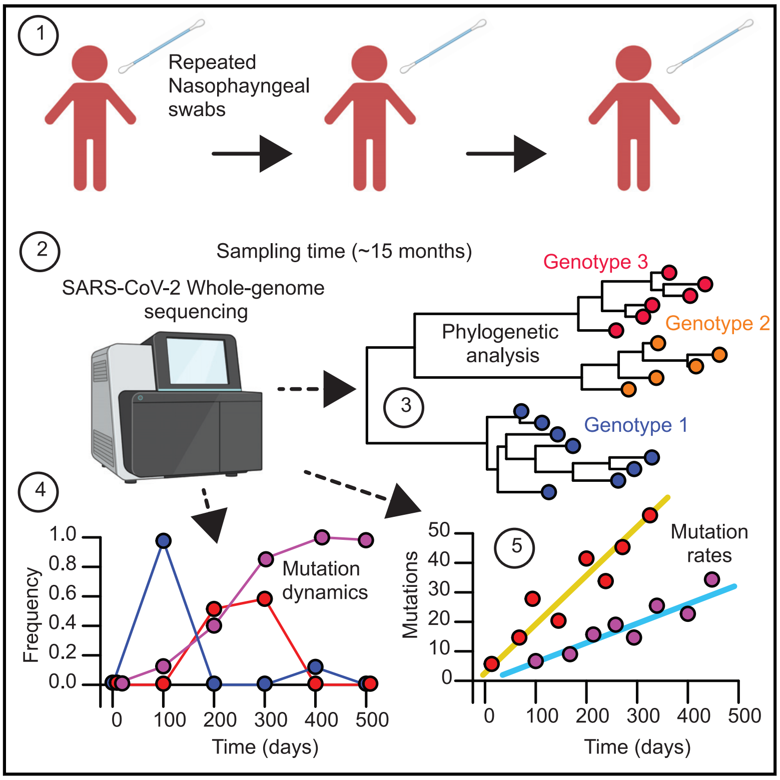

Authors

Chrispin Chaguza, Anne M. Hahn, Mary E. Petrone, ..., Ronald I. Swanstrom, Scott C. Roberts, Nathan D. Grubaugh

Correspondence

chrispin.chaguza@yale.edu (C.C.),
nathan.grubaugh@yale.edu (N.D.G.)

In brief

To understand the intrahost evolution of SARS-CoV-2 from a single patient chronically infected for at least 471 days, Chaguza et al. use whole-genome sequencing to estimate the evolutionary rate, the genetic divergence of viral lineages, relative mutation rates, and the frequency of mutational variants during the course of the infection.

Highlights

- SARS-CoV-2 evolutionary rate is higher during chronic infection than the global rate
- At least three genetically divergent genotypes emerge during the chronic infection
- Variable fluctuation of mutations partially correlates with antibody treatment
- Genetic diversity varies by gene and is highest in the envelope, ORF6, and ORF10

Chaguza et al., 2023, Cell Reports Medicine *4*, 100943
February 21, 2023 © 2023 The Author(s).
https://doi.org/10.1016/j.xcrm.2023.100943

CellPress

Cell Reports Medicine

CellPress
OPEN ACCESS

Article

Accelerated SARS-CoV-2 intrahost evolution leading to distinct genotypes during chronic infection

Chrispin Chaguza,[1,12,*] Anne M. Hahn,[1,12] Mary E. Petrone,[1,2,12] Shuntai Zhou,[3,4] David Ferguson,[5] Mallery I. Breban,[1] Kien Pham,[1] Mario A. Peña-Hernández,[6] Christopher Castaldi,[7] Verity Hill,[1] Yale SARS-CoV-2 Genomic Surveillance Initiative, Wade Schulz,[5,8] Ronald I. Swanstrom,[4,9] Scott C. Roberts,[10] and Nathan D. Grubaugh[1,11,13,14,*]
[1]Department of Epidemiology of Microbial Diseases, Yale School of Public Health, New Haven, CT, USA
[2]School of Life and Environmental Sciences, University of Sydney, Sydney, NSW, Australia
[3]Department of Microbiology and Immunology, University of North Carolina, Chapel Hill, NC, USA
[4]Lineberger Comprehensive Cancer Center, University of North Carolina at Chapel Hill, Chapel Hill, NC, USA
[5]Department of Laboratory Medicine, Yale School of Medicine, New Haven, CT, USA
[6]Department of Biological and Biomedical Sciences, Yale School of Medicine, New Haven, CT, USA
[7]Yale Center for Genome Analysis, Yale University, New Haven, CT, USA
[8]Center for Outcomes Research and Evaluation, Yale New Haven Hospital, New Haven, CT, USA
[9]Department of Biochemistry and Biophysics, University of North Carolina at Chapel Hill, Chapel Hill, NC, USA
[10]Infectious Disease, Yale School of Medicine, New Haven, CT, USA
[11]Department of Ecology and Evolutionary Biology, Yale University, New Haven, CT, USA
[12]These authors contributed equally
[13]Senior author
[14]Lead contact
*Correspondence: chrispin.chaguza@yale.edu (C.C.), nathan.grubaugh@yale.edu (N.D.G.)
https://doi.org/10.1016/j.xcrm.2023.100943

SUMMARY

The chronic infection hypothesis for novel severe acute respiratory syndrome coronavirus 2 (SARS-CoV-2) variant emergence is increasingly gaining credence following the appearance of Omicron. Here, we investigate intrahost evolution and genetic diversity of lineage B.1.517 during a SARS-CoV-2 chronic infection lasting for 471 days (and still ongoing) with consistently recovered infectious virus and high viral genome copies. During the infection, we find an accelerated virus evolutionary rate translating to 35 nucleotide substitutions per year, approximately 2-fold higher than the global SARS-CoV-2 evolutionary rate. This intrahost evolution results in the emergence and persistence of at least three genetically distinct genotypes, suggesting the establishment of spatially structured viral populations continually reseeding different genotypes into the nasopharynx. Finally, we track the temporal dynamics of genetic diversity to identify advantageous mutations and highlight hallmark changes for chronic infection. Our findings demonstrate that untreated chronic infections accelerate SARS-CoV-2 evolution, providing an opportunity for the emergence of genetically divergent variants.

INTRODUCTION

Since the initial introduction of severe acute respiratory syndrome coronavirus 2 (SARS-CoV-2) in late 2019, subsequent coronavirus disease 2019 (COVID-19) waves have been predominantly driven by the emergence of variants with either enhanced transmissibility or the ability to evade human immune responses.[1–7] The SARS-CoV-2 lineage B.1.1.7, designated as Alpha by the World Health Organization (WHO), was the first named variant. Alpha was initially associated with a large cluster of cases in the United Kingdom before spreading globally.[3] Analysis of the phylogenetic branch leading up to the B.1.1.7 clade revealed a faster evolutionary rate compared with the background evolutionary rate,[8] and the clade's defining constellation of substitutions was associated with higher transmissibility compared with other lineages circulating at the time.[9] Similar patterns of an unexpectedly long phylogenetic branch preceding a clade with increased transmissibility, disease severity, or immune evasion have been observed multiple times with other variants, like Beta (B.1.351), Gamma (P.1), Delta (B.1.617.2), and Omicron (B.1.529), causing extensive morbidity and mortality on national and international levels.[1,2,10,11]

Three mechanisms have been proposed for the emergence of genetically divergent SARS-CoV-2 variants: (1) prolonged human-human transmission in an unsampled population, (2) circulation in an unsampled zoonotic reservoir, and (3) chronic infection in an immunocompromised individual. Of these, chronic infection is the most plausible. Cryptic human-human transmission is unlikely

This is an open access article under the CC BY license (http://creativecommons.org/licenses/by/4.0/).

to result in the increased evolutionary rate that is a hallmark of variants. Retrospective sequencing of cases may shorten the length of clade-defining branches, as was the case for Gamma (P.1), which likely emerged through stepwise diversification via multiple interhost transmissions.[12] However, human-animal, followed by animal-human, transmission has been documented repeatedly, particularly in farmed mink populations,[13] but there is no evidence to suggest that these events would produce monophyletic clades observed in most variants. Documented spillovers have not been associated with increased evolutionary rates, nor have they led to community transmission. In contrast, a chronic SARS-CoV-2 infection in an immunocompromised individual is the best explanation for the emergence of Alpha based on evolutionary theory, when gaps in surveillance can be discounted.[8] Compared with between-host transmission, within-host dynamics can lead to increased evolutionary rates because the larger viral population is subject to fewer genetic bottlenecks.[14–16] This increases the selective impact imposed by a semi-functioning immune system relative to drift[17] and, in the case of SARS-CoV-2, increases the opportunity for recombination.[18] While extended community transmission associated with spillovers from animal reservoirs has not been observed, viruses from chronic infections have been detected in the broader community.[19,20] Despite this theoretical and epidemiological evidence that chronic infections could drive the emergence of variants, there is still a need for genomic analyses investigating the prolonged within-host evolutionary dynamics of the virus population in a chronically infected individual.

Previous studies of chronic infections have shown that individuals who are immunocompromised are at an elevated risk of developing a persistent SARS-CoV-2 infection (Table 1).[21–26] However, the majority of these studies have primarily focused on the clinical characteristics of the patients rather than detailed intrahost evolution of the viral genomes during chronic infection. An improved understanding of SARS-CoV-2 evolution during chronic infections could reveal targets for therapeutics to treat these infections and, as discussed above, curb the evolution and emergence of novel genetically divergent variants. In this study, we investigate the intrahost genetic diversity and evolution of the SARS-CoV-2 B.1.517 lineage during 471 days of chronic infection of an immunocompromised individual suffering from advanced lymphocytic leukemia and B cell lymphoma. Here, we characterize the longitudinal dynamics of viral RNA titers and infectious copies, intrahost genetic diversity, mutational spectrum and frequency, and recombination. We observe the accelerated evolution of SARS-CoV-2 during infection, marked by the emergence of distinct coexisting genotypes that could be designated as new lineages if transmitted to the community. We further demonstrate that the mutation accrual patterns of these genotypes resemble those seen in SARS-CoV-2 variants, including Omicron, and describe intrahost evolution dynamics to identify potential hallmark mutations associated with chronic infection. Together, our findings support the hypothesis that chronic infections could lead to the emergence of genetically divergent novel lineages with potentially high transmissibility and immune escape.

RESULTS

Chronic infection driving continued detection of B.1.517 in the United States

We identified the recurrent SARS-CoV-2 lineage B.1.517 in Connecticut (USA), extinct elsewhere in the US and globally, through our SARS-CoV-2 genomic surveillance initiative dataset (started in January 2021 with the emergence of Alpha) (Figures 1A and 1B). The B.1.517 lineage emerged in North America (likely in the US) in approximately early January 2020 (95% confidence interval [CI]: November 2019 to March 2020) (Figure 1C). Following its emergence, B.1.517 spread to several US states and internationally, predominantly causing sporadic cases, except in Australia, where an outbreak occurred (Figure 1D). Sequenced cases of B.1.517 in other countries remained sporadic and relatively low in frequency (Figures 1A and 1B). Lineage B.1.517 circulated until April 2021 in the US and other countries; however, we continued to detect B.1.517 in our genomic surveillance in Connecticut (USA) until March 2022 (Figures 1A and 1B). We traced the recurrent B.1.517 sequences to an immunocompromised individual experiencing a chronic SARS-CoV-2 infection lasting 471 days at the time of writing (Figure 1E; Table 1). Our surveillance system captured 30 nasal swabs from this individual, and we sequenced SARS-CoV-2 genomes from days 79 to 471 (February 2021 to March 2022).

The patient found to be chronically infected with B.1.517 is in their 60s with a history of diffuse large B cell lymphoma and underwent an allogeneic haploidentical stem cell transplantation in 2019. In early 2020, the disease relapsed, and the patient started a new chemotherapy regimen, ultimately requiring chimeric antigen receptor T cell therapy in mid-2020. The patient was noted to have persistent but improving disease until November 2020, when it started to relapse again. This is when the patient first tested positive for SARS-CoV-2 (November 2020, day 0), likely from a household contact that first tested positive for SARS-CoV-2 2 days prior (Figure 2A). The patient was started on palliative radiation therapy on day 278 and was admitted three times from days 279 to 452 for malignancy-related complications. Clinical courses related to the infection are provided in Figure 2A, and longitudinal immune parameters such as immunoglobulin G (IgG) serum levels as well as lymphocyte and T cell counts are provided in Figure S1. The patient's IgG levels were within or near the reference range when receiving regular intravenous Ig therapy (IVIG) infusions until day 205, then the IgG levels dropped after IVIG treatment was suspended. The patient also had low lymphocyte, T cell, and IgA (non-detectable, data not shown in Figure S1) levels before and during the infection, consistent with their immunocompromised state.

Aside from the initial presentation of several days with mild upper respiratory tract symptoms not requiring oxygenation or hospitalization, the patient has remained asymptomatic for the duration of their SARS-CoV-2 infection. The only COVID-19 treatment the patient received was a bamlanivimab (LY-CoV555) monoclonal antibody infusion on day 90, after which the patient did not wish to obtain any additional COVID-19 therapies or vaccines. The patient continues to test positive for SARS-CoV-2 471 days and counting after the initial diagnosis.

CellPress
OPEN ACCESS

Table 1. Summary of persistent SARS-CoV-2 infections in immunocompromised individuals reported to date

Lineage	Age (years)	Total days	Country	Patient condition	Infection outcome	Reference
B.1.517	60s	471; ongoing	USA	multiple sclerosis, chronic lymphocytic leukemia	ongoing	present study
20B	70–79	101	UK	diagnosed with B cell lymphoma	died	Kemp et al.[26]
B.1.1.27	30s	216	South Africa	advanced HIV disease	cleared	Cele et al.[21]
B.1.1 or 20B	58	145	Germany	autosomal dominant polycystic kidney disease (kidney transplant)	cleared	Weigang et al.[24]
Unknown	45	154	USA	severe antiphospholipid syndrome (APS)	died	Choi et al.[27]
A.1	71	70	USA	chronic lymphocytic leukemia and acquired hypogammaglobulinemia	cleared	Avanzato et al.[25]
Unknown	71	60	China	history of intermittent fever	cleared	Li et al.[28]
Unknown	75	>333	Denmark	chronic lymphocytic leukemia	cleared	Monrad et al.[29]
B.1.351	22	>270	South Africa	poorly controlled HIV infection	cleared	Maponga et al.[22]
B.1 or 20C	70s	292	USA	stage IV non-Hodgkin's lymphoma, acquired B cell deficiency	cleared	Gandhi et al.[23]
Multiple	unknown	218	UK	immunocompromised, condition not specified	unknown	Wilkinson et al.[30]
B.1.332	48	335	USA	type 2 diabetes mellitus, B cell depletion, remission from large B cell lymphoma	cleared	Nussenblatt et al.[31]
B.52	80s	311	UK	chronic lymphocytic leukemia and hypogammaglobulinemia	unknown	Williamson et al.[32]
BA.1	unknown	81	USA	immunocompromised, condition not specified	cleared	Gonzalez-Reiche et al.[20]
20A, 20C	3, 21, 2	27, 144, 162	USA	B cell acute lymphoblastic leukemia	cleared	Truong et al.[33]
B.1.1	47	132	Russia	non-Hodgkin's diffuse B cell lymphoma IV stage B	cleared	[a]
B.1.617.2	53, 67	94, 97	Singapore	acute myeloid leukemia; splenic marginal zone lymphoma and status post splenectomy	cleared	Ko et al.[34]
B.1.1.401	61	171	Portugal	non-Hodgkin's lymphoma	cleared	Borges et al.[35]
Unknown	73	74	USA	treatment-refractory multiple myeloma	cleared	Hensley et al.[36] McCarthy et al.[37]
B.1.1	70s,	154	Germany	follicular lymphoma	died	Khatamzas et al.[38]
B.1.2	50s	∼480; ongoing	USA	immunocompromised, condition not specified	ongoing	Halfmann et al.[39]
B.1.1.50	unknown	56, 65, 88, 36, 75, 37	Israel	chronic lymphocytic leukemia; follicular lymphoma; Hodgkin's lymphoma; autoimmune skin disease; acute lymphoblastic leukemia	cleared	Harari et al.[40]
Unknown	unknown	218	UK	Wiskott-Aldrich syndrome	cleared	Bradley et al.[41]
AY.43	60s	>240	Israel	malignant melanoma, diffuse large B cell lymphoma and squamous cell carcinoma	ongoing	Shapira et al.[42]

[a]https://virological.org/t/emergence-of-y453f-and-69-70hv-mutations-in-a-lymphoma-patient-with-long-term-covid-19/580.

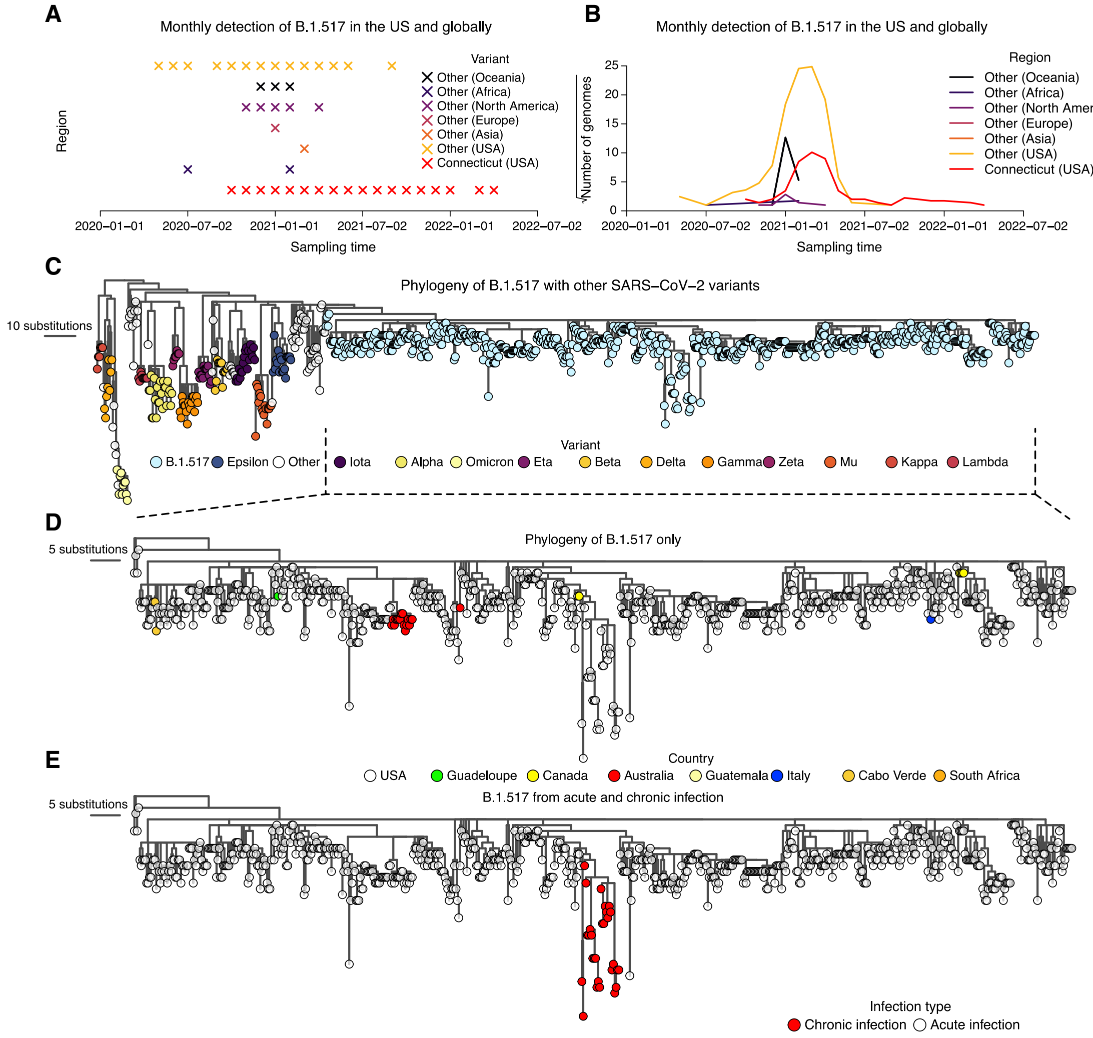

Figure 1. Genomic surveillance and phylogeny showing continued detection and genetic divergence of B.1.517 from chronic infection

(A) Monthly detection of B.1.517 (B.1.517 and B.1.517.1) variants in Connecticut (USA), other US states, and elsewhere.

(B) The total number of sequence genomes for the B.1.517 (B.1.517 and B.1.517.1) variants in Connecticut (USA), the rest of the US, and elsewhere. The y axis is transformed by square root transformation to show time points with non-zero number of genomes, especially those from countries with a low prevalence of B.1.517.

(C) A maximum likelihood phylogeny of B.1.517 in the context of selected genomes from other variants.

(D) A maximum likelihood phylogeny of all sequenced B.1.517 genomes showing country of origin.

(E) A maximum likelihood phylogeny of all sequenced B.1.517 samples highlighting the genomes associated with the chronic infection and other contextual genomes from acute infection (although some could have been sampled from unknown chronic infections).

Persistently high viral RNA copies and infectious virus detected throughout the course of the chronic infection

To track the dynamics of the patient's chronic infection, we quantified the viral RNA titers and investigated the virus infectivity from days 79 to 471 post-diagnosis (February 2020 to March 2022; Figure 2; Tables S1 and S2). The median number of days between successive samples was ~14 days, 95% CI: 8–20). We could not obtain samples from the patient prior to day 79 as they were collected before the establishment of our SARS-CoV-2 biorepository and genomic surveillance initiative. Though the infection has not yet cleared at the time of writing, sample collection was halted in March 2022 due to complications relating to the B cell lymphoma disease, precluding further nasopharyngeal sampling.

We measured SARS-CoV-2 viral genome copies using RT-PCR and performed whole-genome sequencing on 30 samples. We tested a subset of twelve for infectious virus and found that the individual was infectious with high virus copies for almost the entire duration of their infection (Figure 2B). Nasal swab samples collected from days 79 to 471 post-diagnosis had a mean RT-PCR cycle threshold (Ct) of 25.50 (range: 15.6–34.1), equivalent to 3.10 × 10^8 virus genome copies per mL (range: 7.30 × 10^4–6.04 × 10^9), though the genome copies numbers tended to decrease over time (Figure 2B; Table S1). Of the 12 swab

CellPress
OPEN ACCESS

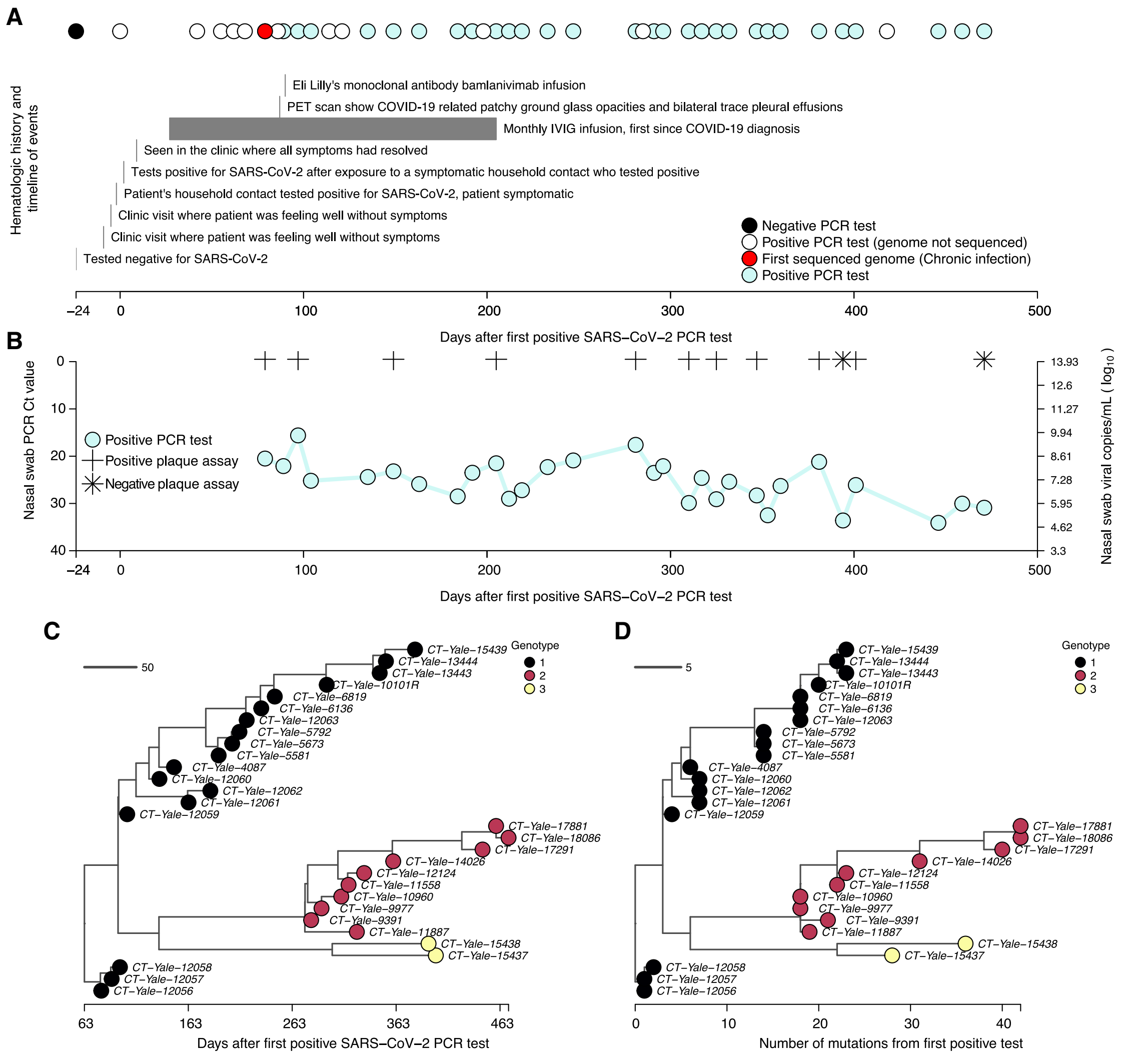

Figure 2. Molecular and virological assays showing isolation of infectious viruses with high copy numbers and the emergence and coexistence of distinct genotypes during the chronic infection

(A) Timeline showing clinical history of the patient from the earliest time they tested negative for SARS-CoV-2, the first positive test following household exposure by a symptomatic household contact who tested positive 2 days prior, until the last sampling point. Note that collection of samples was stopped due to the deteriorating condition of the patient, but the infection had not yet cleared.

(B) Nasal swab RT-PCR cycle threshold (Ct) values for the samples available for whole-genome sequencing showing high viral RNA copy numbers. Additionally, virus infectivity assays performed for selected samples revealed infectious virus at most sampling points. Additional information for the samples, including plaque assay results, are provided in Table S1.

(C) Time-resolved phylogeny of the chronic infection samples with branch lengths scaled by the number of days since the first positive RT-PCR SARS-CoV-2 test. The phylogeny was generated based on near full whole genomes after trimming the 3′ and 5′ ends to remove poor quality nucleotides (see STAR Methods).

(D) Maximum likelihood phylogeny of the chronic B.1.517 samples showing branch lengths scaled by the genetic divergence expressed as the number of accrued substitutions over time. The phylogeny shows the intrahost emergence and persistence of multiple divergent genotypes.

samples that we tested for the presence of the viable virus, the infectious virus could be detected *in vitro* from ten sampling points (between days 79 and 401) but not on days 394 and 471, corresponding to samples with higher Ct values (33.6 and 30.9, respectively; Figure 2B; Table S1). However, the patient has been presumed to be asymptomatic for COVID-19 after the resolution of the initial acute infection in November 2020, and all the patient's admissions were secondary to malignancy. Given the sustained high viral load and infectiousness of viral particles in the nasopharynx, we concluded that the patient's immune system was unable to suppress active SARS-CoV-2 replication throughout the infection (Figure S1).

Three distinct virus genotypes emerged during chronic infection

We hypothesized that SARS-CoV-2 from prolonged chronic infection would diversify into distinct populations, reflecting infection of spatially structured human cells and tissues.

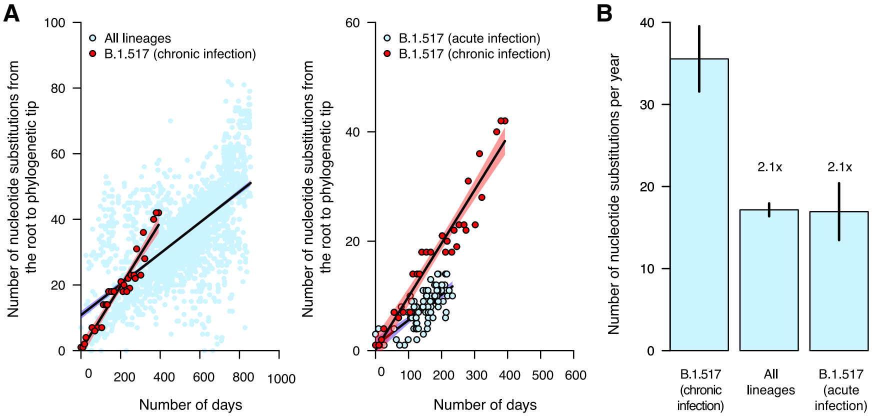

Figure 3. Nucleotide substitution rates are faster during chronic infection than acute infection and the global evolutionary rate

(A) Scatterplots showing the relationship between phylogenetic root to tip distances, expressed as the number of nucleotide substitutions per site, and time as the number of days from the first sampled genome for the B.1.517 from chronic infection versus all SARS-CoV-2 lineages and other B.1.517 from acute infections. The data points associated with the chronic infection are colored in red, while those representing other variants are colored in sky blue. The lines and shaded bands surrounding them represent the linear regression models fitted to the data points for the chronic infection data and other variants.

(B) Bar graph showing the average mutation rates, expressed as the number of nucleotide substitutions per year for the chronic infection samples and other variants based on the regression coefficients (β) generated from the plots in (A). Specific values for the evolutionary rates for all lineages combined, the parental and chronic infection B.1.517 strains, and other lineages are shown in Table S3 and Figure S3.

SARS-CoV-2 can infect diverse human cell populations and tissues,[43] similar to other pathogens including influenza virus[44,45] and bacterial pathogens.[46,47] To test this hypothesis, we constructed a phylogeny of the 30 longitudinally sequenced SARS-CoV-2 genomes from days 79 to 471 since the first positive SARS-CoV-2 test.

We identified three genetically divergent genotypes based on the phylogenetic clustering (numbered 1–3), which emerged and coexisted during the infection (Figures 2C and 2D). While we first sequenced genotype 1 on day 79, we cannot confirm that it was the founding genotype due to missing earlier samples. Genotype 1 accumulated up to 24 nucleotide substitutions (13 amino acid substitutions) through day 379 in a ladder-like evolutionary pattern. Genotype 2 diverged from genotype 1, with a maximum of 40 nucleotide substitutions (28 amino acid substitutions) from days 281 to 471. Genotype 3 also diverged from genotype 1 into two sister subgenotypes sampled on days 394–401. The first subgenotype accumulated 37 nucleotide substitutions (30 amino acid substitutions), while the second subgenotype contained 29 nucleotide substitutions (27 amino acid substitutions) and diverged from each other on day ~316 (95% CI: ~288–336). These findings support our hypothesis that the founding B.1.517 virus independently diverged into coexisting genetically distinct populations.

Though the identified genotypes coexisted for the duration of the infection, the relative composition of the viral population changed over time (Figures 2C and 2D). We found that genotype 1 was dominant in nasal swabs from days 79 to 247; however, from days 281 to 471, the dominant genotype frequently switched between the three. From day 281 to 381, the sampled dominant genotype alternated between genotypes 1 and 2 five times. Genotype 3 became dominant on days 394 and 401 before being replaced again by genotype 2 from days 446 to 471. The rapid and sometimes temporary replacement of genotypes during this infection suggested continual reseeding of the nasopharynx with distinct virus populations that likely independently evolved elsewhere in the body.[48]

We then compared the B.1.517 sequences from the patient with the chronic SARS-CoV-2 infection against other B.1.517 sequences from Connecticut (USA) to identify potential onward transmission into the wider population. Our phylogenetic analysis showed separate clustering of the chronic infection sequences from the rest of the sequence cases from the population, demonstrating that there was no detectable onward transmission (Figure S2). These findings were consistent with the clinical observations that the patient had become reclusive, which would minimize the potential transmission of the evolved intrahost genotypes into the community.

SARS-CoV-2 evolution was accelerated during the chronic infection

The within-host evolutionary rate of microbes tends to exceed rates observed at the population level because of the absence of stringent bottlenecks imposed by transmission.[27,49] We thus hypothesized that the SARS-CoV-2 evolutionary rate during this chronic infection would be higher than the estimated global evolutionary rate. To test this hypothesis, we randomly sampled an equal number of genomes from the global dataset, ~1 to 3 genomes per continent per month (n = 2,539), for the WHO-designated SARS-CoV-2 variants and performed a regression of distance from the root of the phylogeny against the time of sampling for the global dataset and the sequences from the chronic infection (Figures 3 and S3). We found that the evolutionary rate during the chronic infection was 35.55 (95% CI: 31.56–39.54) substitutions per year or ~1.21×10^{-3} (95% CI: 1.07×10^{-3}–1.34×10^{-3}) nucleotide substitutions per site per year (s/s/y). This was ~2 times higher than our estimated average global (all lineages) SARS-CoV-2 evolutionary rate (5.83×10^{-4} [95% CI: 5.56×10^{-4}–6.11×10^{-4}] s/s/y; Figures 3A, 3B, and S3; Table S3). Our estimate for the global evolutionary rate, based on a careful random sampling of representative genomes from GISAID per month per variant, is within the expected range of what is reported in other studies that use the same regression method.[8,50] It is worth noting that estimates of the background rate of evolution vary due to different methodologies and downsampling used: 8×10^{-4} s/s/y is commonly used in phylodynamic analyses,[51–53] and the current (June 2022) Nextstrain estimate is approximately 9.9×10^{-4} s/s/y. However, even at these upper ends of the rate estimates, the rate

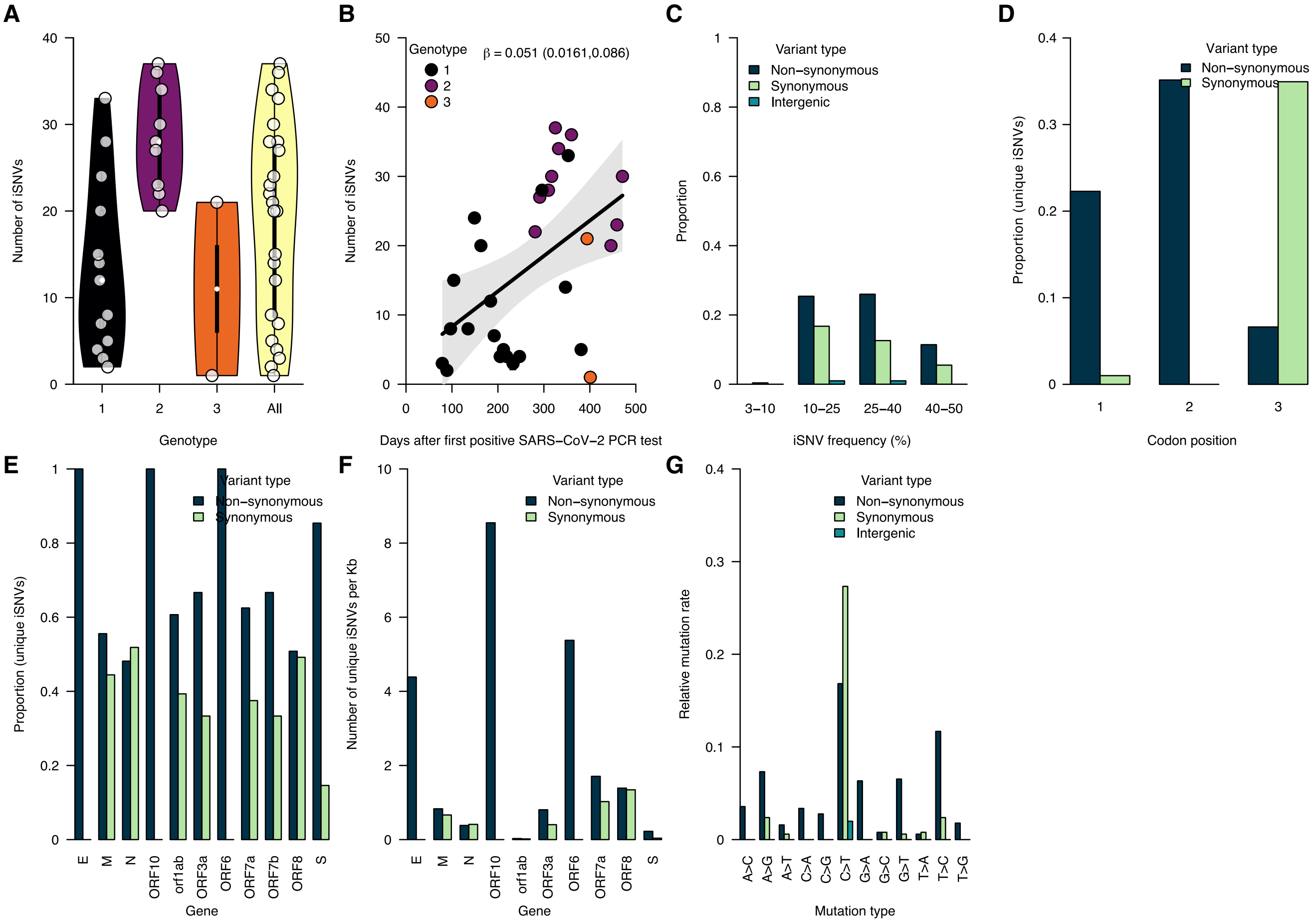

Figure 4. Increasing intrahost genetic diversity during chronic infection

(A) The number of intrahost single-nucleotide variants (iSNVs) >3% frequency across all the samples and genotypes detected during the infection (see Figures 2C and 2D).

(B) The number of iSNVs accumulated over time during the chronic infection. The black solid line represents a fitted linear regression.

(C) Proportion of iSNVs binned at different frequencies and stratified by variant or mutation type (intergenic, synonymous, and non-synonymous).

(D) The proportion of the overall number of unique iSNVs coding for synonymous and non-synonymous amino acid changes at different codon positions.

(E) The proportion of unique iSNVs grouped by variant type to highlight potential selection across different SARS-CoV-2 genes.

(F) The number of unique iSNVs per gene normalized by the gene length to highlight variability in selection independent of gene size.

(G) The mutation spectra showing the relative mutation rate across the SARS-CoV-2 genome-stratified variant type. Additional information for all the identified mutations (intergenic, synonymous, and non-synonymous) are provided in Data S1.

of evolution in the chronic infection documented here is still faster. Our estimated evolutionary rate of this chronic B.1.517 infection is also ∼2 times higher than the evolutionary rate for the parental B.1.517 lineage (5.76×10^{-4} [95% CI: 4.58×10^{-4}–6.94×10^{-4}] s/s/y). These findings show that this chronic infection resulted in accelerated SARS-CoV-2 evolution and divergence, a mechanism potentially contributing to the emergence of genetically diverse SARS-CoV-2 variants, including Omicron, Delta, and Alpha.

Increasing intrahost genetic diversity and variable gene-specific evolutionary rates during the chronic infection

Having detected three genotypes and observed the overall increased SARS-CoV-2 evolutionary rate during chronic infection, we hypothesized that intrahost virus genetic diversity would also increase over the course of infection. To test this hypothesis, we used deep sequencing to quantify the number of unique intrahost single-nucleotide variants (iSNVs; i.e., "mutations") present at >3% within sample frequency in each sample (Figures 4A–4D). To validate the iSNV frequencies that we generated from whole-genome amplicon-based sequencing, we sequenced the spike gene of a subset of the samples using unique molecular index (UMI)-tagged primers that improve the accuracy of iSNV detection.[54,55] We found a high concordance between the iSNV frequencies measured from our whole-genome amplicon-based and UMI sequencing (median [β]: 0.999) (Figure S4; Table S4).

The number of iSNVs increased over time across all three genotypes, and the viral effective population size (N_e) fluctuated similarly. We observed a variable number of iSNVs per sample

(mean: 32.07, range: 2–65). Genotype 2 comprised more iSNVs than genotype 1, which emerged earlier in the infection (Figure 4A). We used regression to assess the accrual rate of iSNVs and found a strong positive association between the number of iSNVs and sampling time (regression slope [β]: 0.013, 95% CI: 0.058–0.148 iSNVs per day) (Figure 4B). Next, we assessed the dynamics of the N_e from the sequenced consensus genomes during the chronic infection using a coalescent Bayesian skyline model.[56] The dynamics of the N_e estimates mirrored those of the number of unique iSNVs, especially in the early stages of the chronic infection, and peaked at ~370 days post-diagnosis (Figure 4C). Finally, we characterized the iSNVs with frequencies between 3% and 50% and found that ~40%–45% of the iSNVs found in intergenic regions, and those associated with synonymous and non-synonymous amino acid changes in genic regions, rose to frequencies of 40%–50% during the infection (Figure 4D). These patterns were consistent for intergenic, synonymous, and non-synonymous iSNVs. Such high iSNV frequencies combined with the increasing number of iSNVs (Figures 4B and S5) are in line with the coexistence of multiple genotypes within a sequenced sample and help to explain the consensus genotype switching that we described after day 281 of the infection (Figures 2C and 2D). Collectively, these data support our hypothesis that intrahost SARS-CoV-2 genetic diversity increased with time during the chronic infection to levels not typically reported during acute infections.[15,57]

We investigated the potential impact of this diversity on virus evolution by analyzing the types of mutations and the gene-specific evolutionary rates during the chronic infection (Figures 4E–4G). Stratifying the >3% iSNVs by codon position, we found that most occurred at the second and third codon positions (Figure 4D). Most of the substitutions at the first and second codon positions resulted in ~22% and ~35% non-synonymous changes, respectively, compared with 0.07% at the third codon. Because these changes could correspond to selection in different genes, we compared the proportion of synonymous and non-synonymous iSNVs. We hypothesized that the spike and other surface and membrane-associated proteins would have a higher abundance of non-synonymous amino acid changes than other genes as the principal targets of the host antibody-mediated immune response. Consistent with our hypothesis, we found a statistically higher abundance of non-synonymous changes than synonymous changes only in the spike glycoprotein (abundance: ~85%, $p = 4.96 \times 10^{-11}$) but not in the envelope (abundance: ~100%, $p = 0.248$), membrane (abundance: ~55%, $p = 0.70$), and nucleocapsid (abundance: 48%, $p = 1$) genes (Figure 4E). We also found a higher abundance of non-synonymous amino acid changes in a non-structural gene, namely ORF1ab polyprotein (abundance: ~61%, $p = 0.001$). We normalized the estimates to account for the gene length to compare the abundance of synonymous and non-synonymous changes in different genes. Contrary to our hypothesis that the genes encoding the surface and membrane-associated proteins (spike, envelope, and membrane) would have the highest normalized frequency of non-synonymous changes, the highest frequencies occurred in the ORF10 gene, followed by ORF6 and envelope, while lower frequencies occurred in the other genes, including spike and membrane (Figures 4F and S6). These differences suggested that other genes evolved faster than the spike gene during this chronic infection. Finally, the mutation spectra showed relatively higher C→T substitution rates, consistent with findings elsewhere,[57–59] but we found that the C→T substitution equally resulted in synonymous and non-synonymous changes. In contrast, some substitutions, including A→G, G→A, G→T, and T→C, appeared to cause slightly more non-synonymous than synonymous changes (Figures 4G and S7). Our findings suggest that the accelerated evolution during this infection resulted in a variable accumulation of potentially advantageous substitutions across the SARS-CoV-2 genome.

Persistently detected mutations associated with major variants

We hypothesized that specific iSNVs, particularly in the spike glycoprotein gene, were selectively advantageous and therefore were more prevalent than iSNVs in other genes. We tested this hypothesis by comparing the number of unique iSNVs across different samples between the spike and other genes (Figures 5A, 5B, and S8). Overall, we found no differences between the prevalence of unique spike and non-spike iSNVs across different samples ($p = 0.935$). We then investigated if the frequency of the non-synonymous iSNVs across the samples was higher than intergenic and synonymous mutations. Again, we found a similar prevalence of non-synonymous compared with intergenic ($p = 0.912$) and synonymous iSNVs ($p = 0.680$) and between intergenic and synonymous iSNVs ($p = 0.499$). These findings demonstrated that the average persistence of iSNVs from different genes, regardless of their frequency of occurrence, was similar during the course of the infection.

While the distribution of mutations was not concentrated in the spike gene, some specific iSNVs could have been selectively advantageous and/or clinically important. Of the 98 iSNVs detected in more than one sample at >3% intrahost frequency, we found 17 changes in the spike gene, of which ~88% were non-synonymous (Figures 5A, 5B, and S9). The two most common iSNVs, found in 11 of the 30 (36%) whole-genome deep-sequenced samples, were in the ORF8 gene, namely F67S and F120F, while spike:Q493K was the most common spike iSNV and the third most common overall, which may promote adaptation during persistent SARS-CoV-2 infection in humans as seen in murine infection models[19,60] (Figure 5B). Other common spike iSNVs included W64C and T1027I, found in 9 samples.

We also detected several other iSNVs in the spike gene that have clinical relevance and/or are found in other variants. For example, the patient was treated with bamlanivimab (LY-CoV555) on day 90, and we detected two spike gene iSNVs associated with resistance to this antibody: Q493R and E484K.[61–65] In addition, we detected spike:Q493R (found in Omicron) in 5 samples, with the first on day 97, 1 week after bamlanivimab treatment (Figure 6), while the spike:E484K mutation (found in Beta, Gamma, Eta, Iota, and Mu) was detected in five samples from days 104 to 184. These findings provide further evidence that clinically relevant mutations, such as those that confer resistance to antibodies and that are found in other variants, can evolve during the course of chronic infection.

CellPress
OPEN ACCESS

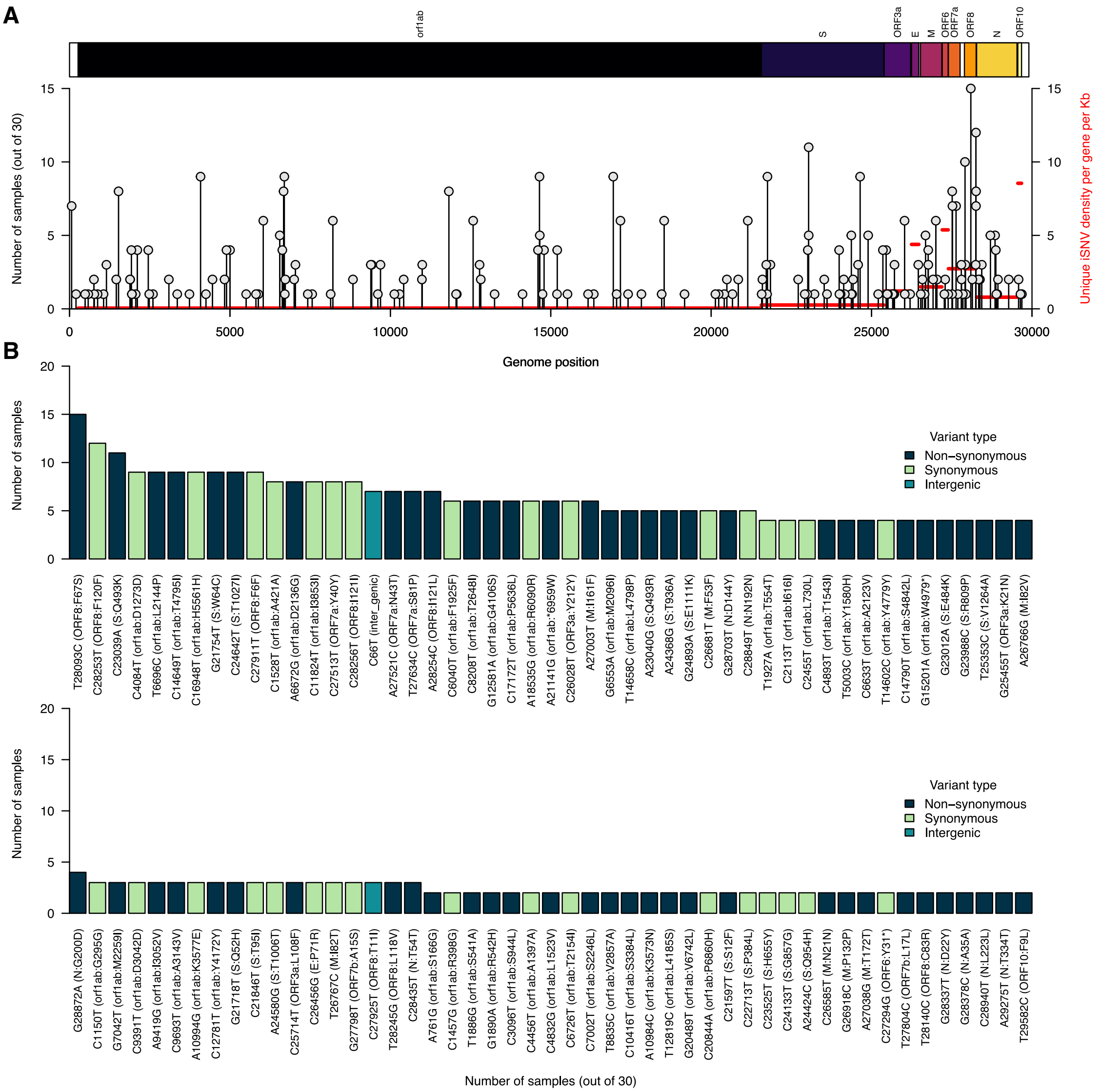

Figure 5. Several intrahost SNVs repeatedly detected during chronic infection

(A) The number of samples containing each unique iSNV and its position on the ancestral SARS-CoV-2 reference genome (GenBank: MN908937.3 or NC_045512.2). The y axis labels represent iSNVs corresponding to specific nucleotide substitutions and position in the genome, while the information within the brackets shows the specific amino acid changes, gene, and position in the gene. The y axis on the right side of the graph, colored in red, shows the average number of iSNVs per kilobase for each gene in the reference genome.

(B) The y axis shows the number of samples containing iSNVs shown on the x axis. The iSNV labels contain the specific nucleotide substitutions and position in the genome. Specific amino acid changes and their specific position in the SARS-CoV-2 genomes are shown in the brackets on the x axis. The bars representing different nucleotide substitutions are colored based on the sequence feature annotations in the ancestral reference genome (GenBank: NC_045512.2). All the iSNVs are colored by the variant or mutation type based on the ancestral SARS-CoV-2 genome sequence feature annotations (GenBank: MN908937.3). Additional information for all the identified mutations (intergenic, synonymous, and non-synonymous) are provided in Data S1.

Temporal mutational dynamics suggest hallmarks of chronic infection

To further understand spike gene iSNVs of potential significance during the chronic infection, we investigated temporal changes in their frequencies using deep sequencing validated with highly accurate UMI-based sequencing (Figures 6 and S10). We hypothesized that the frequency of beneficial non-synonymous spike gene iSNVs likely increased to reach near fixation during the infection. We found two iSNVs, spike:R809P between the fusion peptide and heptapeptide repeat sequence 1 (HR1) regions and spike:T936 A/N in the HR1 region of the spike gene, that increased to near fixation throughout the infection, suggesting they were potentially beneficial to all the coexisting genotypes or reflected high stochasticity due to a low effective population size of the virus (Figure 6). Another notable spike mutation in the receptor-binding domain (RBD), spike:E484K, initially increased in frequency early in the chronic infection, as seen elsewhere,[27] but was replaced by potentially

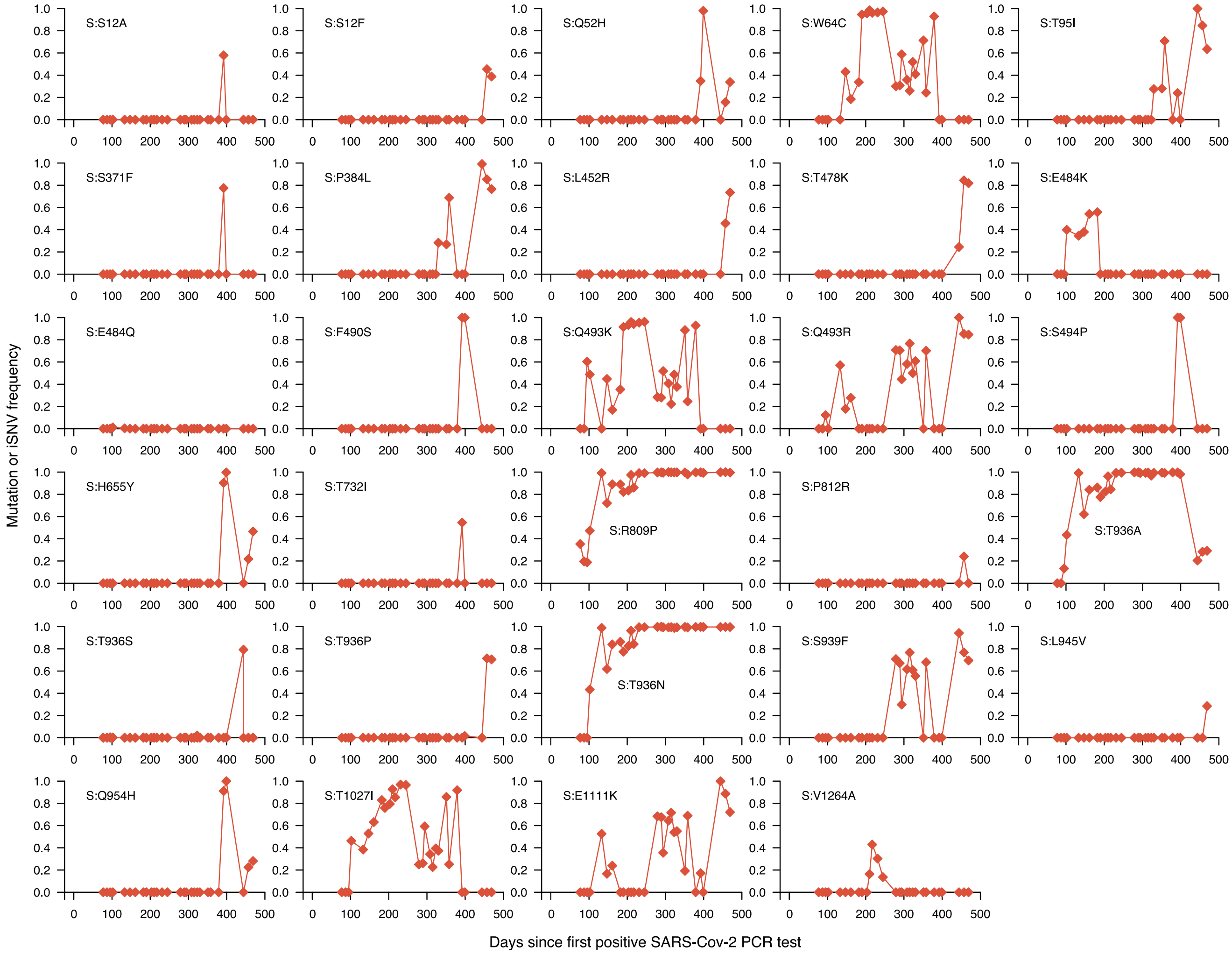

Figure 6. Fluctuating dynamics of iSNVs in the spike gene during chronic infection

Temporal frequencies of 29 non-synonymous iSNVs identified in the spike gene. Additional information for all the identified mutations (intergenic, synonymous, and non-synonymous) are provided in Data S1.

fitter mutations and genotypes. Other spike iSNVs appeared to reach fixation, correlating with the detection of specific genotypes. These included spike:1027I (genotype 1); spike:F490S (RBD; genotype 3); spike:Q52H (genotype 3); spike:P384L (RBD; genotype 2); and spike:493K (RBD; genotype 1). Outside of the spike gene, we detected other iSNVs that appeared to reach fixation: ORF1ab:T1543I (nsp3; genotype 2); ORF1ab:T2154I (nsp3; genotype 1 and 2); ORF1ab:S3384L (nsp5; genotype 3); ORF1ab:G4106S (nsp8; genotype 2); and ORF1ab:A3143V (nsp4; genotype 2; Figure S10), We conclude that most iSNVs fluctuate in frequency and rarely reach fixation. In contrast, a few spike iSNVs, which are novel and previously identified in variants and chronic infections elsewhere, attain fixation. We interpreted this as evidence of a selective advantage, possibly reflecting the escape of the host antibody-mediated immune response, but we could not rule out other neutral evolutionary processes.

No evidence for intrahost recombination during chronic infection

The long duration of this infection, which spanned the emergence of multiple variants (e.g., Alpha, Delta, Omicron), provided favorable conditions for recombination. The occurrence of recombination in the SARS-CoV-2 genome has been demonstrated.[18,66] Therefore, we hypothesized that recombination may have occurred during the chronic infection between coexisting B.1.517 genotypes and between B.1.517 genotypes and other circulating variants transiently causing undetected coinfections. To test this hypothesis, we conducted a recombination analysis of the consensus genomes generated from the persistent infection samples. Since multiple genotypes emerged during the chronic B.1.517 infection, we first investigated the occurrence of intrahost recombination among these genotypes during the infection. We then tested whether recombination occurred between the B.1.517 chronic infection strains and

other non-B.1.517 variants detected in Connecticut (USA), especially Delta and Omicron lineages. We found no evidence of recombination between the chronic B.1.517 genotypes or other variants. These findings suggested that the emergence of multiple genotypes during the B.1.517 infection evolved independently from the ancestral B.1.517 following infection due to random mutational processes rather than intrahost recombination.

DISCUSSION

In our comprehensive genomic investigation, we characterized the intrahost genetic diversity and evolution of SARS-CoV-2 during a chronic infection that has persisted for over a year. Our phylogenetic analysis, based on sequencing 30 nasal swab samples from days 79 to 471 post-diagnosis, revealed accelerated SARS-CoV-2 evolution and the emergence and coexistence of multiple genetically distinct genotypes—a finding not reported in other studies reflecting the duration of the infection and longitudinal sampling. These distinct genotypes appeared to emerge as early as within the first 3 months of the infection, although new genotypes were detected after nearly 10 months, suggesting that multiple novel variants may simultaneously emerge and potentially spread from the same immunocompromised individual over a longer sampling period. Supporting this point, we detected high viral RNA copies and infectious viruses throughout the duration of infection even though the patient remained asymptomatic for COVID-19. A strength of this study was our ability to collect samples for a substantial portion of the infection because it enabled us to document the patient's prolonged infectiousness. This critical finding could potentially be missed if data from chronic infections collected over shorter timescales were used. Our study provides evidence that chronic SARS-CoV-2 infections could be a source for the emergence of genetically diverse variants capable of causing future COVID-19 outbreaks.

During this infection, the viral population accrued twice as many nucleotide substitutions per year as those driving acute infections. Our findings support the prevailing hypotheses that chronic infections in immunocompromised individuals could be the most likely mechanism driving the unpredictable emergence of genetically diverse SARS-CoV-2 variants.[27,67–71] We have shown that the accelerated evolution observed in other SARS-CoV-2 variants such as Omicron and Alpha, which are considered to have emerged during unknown chronic infection, is consistent with the accrual of nucleotide substitutions demonstrated in our study.[8,10,21] Although previous studies have reported that most SARS-CoV-2 populations associated with chronic infections are homogeneous, we found multiple genotypes coexisting throughout a single infection. The prolonged infectiousness of this patient demonstrated that a single chronic infection could cause onward transmission of multiple genetically distinct SARS-CoV-2 variants into the broader population. This could be especially problematic as many people with chronic infections, as was the case with this patient, remain mostly asymptomatic for COVID-19 and may feel well enough to resume regular interactions with other people. The direct, onward transmission of B.1.616 and BA.1 lineage from chronic infections has already been documented.[19,20] Therefore, it is possible that the simultaneous emergence of divergent Omicron sublineages (e.g., BA.1 and BA.2) could have been from a single long chronic infection.[10,21] Altogether, our findings suggest that a novel variant could evolve into genetically divergent forms during a single chronic infection.

We speculate that the emergence and disappearance of multiple genotypes reflect virus competition in the nasopharyngeal niche and/or isolated evolution in different compartments of the respiratory tract or other tissues. These compartments may act as reservoirs for the genotypes and reseed them into the nasopharynx, leading to their fluctuating dynamics that can be observed in the swab material. A similar phenomenon has been reported in studies of acute SARS-CoV-2 infection[48] and chronic bacterial infections.[46,47,72] Infection of multiple tissues leads to spatial isolation and niche partitioning, which ultimately reduces intrahost competition between distinct genotypes and promotes the coexistence of numerous genotypes over longer timescales.[46,47] Niche partitioning is plausible because different SARS-CoV-2 variants preferentially infect different cell types.[73] Recent studies have demonstrated that Omicron has evolved a shift in the cellular tropism toward cells expressing transmembrane protease serine 2 (TMPRSS2), allowing it to more effectively infect upper airway cells compared with endothelial cells of the lung, unlike other lineages.[73] This process may similarly occur during accelerated SARS-CoV-2 evolution in chronically infected persons. While intrahost recombination may accelerate intrahost divergence,[18,66] we did not find evidence for recombination leading to the distinct genotypes found during this chronic infection. This might be an indication of the separated spatial distribution of the viral populations, as recombination events would be expected if different genotypes were to be found in the same tissues and cells. The differences in transmission fitness and cellular tropism among these genotypes require further investigation.

The SARS-CoV-2 spike is a homotrimeric transmembrane glycoprotein critical for receptor recognition and cell attachment and entry and an immunodominant target for host immune responses.[74] We found a higher abundance of non-synonymous than synonymous changes in five of the eleven SARS-CoV-2 genes, including the spike. This suggests positive selection during the course of the infection. Interestingly, although we detected the spike:E484K substitution, it did not reach fixation and lasted for approximately 3 months following bamlanivimab (LY-CoV555) treatment. This suggests that despite E484K being associated with antibody evasion,[65,75,76] it is not necessarily a hallmark of chronic infection involving an immunocompromised person, consistent with previous reports[19] since we propose that iSNVs that reached near fixation (spike R809P and T936 A/N) could be selectively advantageous during chronic infection. However, the trajectories of the majority of the mutations showed random fluctuation over time, suggesting weak selection overall and a predominance of neutral evolution. Furthermore, we hypothesize that spike Q493 K/R mutation could be important for chronic SARS-CoV-2 infections,[19,27,77] even though neither became fixed in our study because they were on different genotypes. By validating the iSNV frequencies using a UMI-based sequencing approach (Primer ID), which

helps to remove PCR artifacts,[54,55] our findings provide a robust assessment of intrahost evolutionary dynamics during chronic infection.

Chronic SARS-CoV-2 infections have been reported in individuals with compromised immunity due to a myriad of factors, including advanced HIV, cancer, organ transplant recipients, kidney disease, and autoimmune disorders.[21–27,31] These infections may drive the rapid evolution of SARS-CoV-2 variants, including from lineages considered to be less virulent, which may spread into the broader population after acquiring mutations promoting increased intrinsic transmissibility and immune escape. As seen with Alpha, which cryptically evolved for >1 year before causing a global epidemic,[10] variants that are likely to cause major future outbreaks could be “lying in wait” in unknown chronic infections. Therefore, control measures for COVID-19 should not only include decreasing cases associated with prevailing variants but also identifying and treating chronic infections to disrupt the potential emergence of novel variants. Moreover, since immunocompromised individuals typically exhibit greater healthcare-seeking behavior, implementation of proactive surveillance of chronic SARS-CoV-2 infections could substantially limit the rate of SARS-CoV-2 evolution.[78,79] Considering that novel variants can emerge and transmit globally from anywhere, as seen with Omicron,[10] these measures need global adoption to maximize their benefits.

In this study, we have shown accelerated intrahost evolution and genetic diversity of SARS-CoV-2 during a chronic infection lasting more than 1 year. Our findings show evolutionary patterns resembling those seen leading up to the Alpha and Omicron variants, highlighting the critical role of chronic SARS-CoV-2 infections in the emergence of novel variants. Therefore, we recommend proactive genomic surveillance of immunocompromised individuals to identify and treat potential chronic infections early, increased global equitable access and uptake of primary and booster COVID-19 vaccine regimens, and continued investment in the development of pan-β-coronavirus vaccines,[80,81] to reduce the likelihood of chronic infections.[78] These strategies could halt the accelerated evolution of SARS-CoV-2 seen in chronically infected individuals, disrupting the emergence of genetically divergent and more transmissible variants, ultimately averting mortality, morbidity, and the tremendous economic impacts of strict COVID-19 prevention and control measures.

Limitations of the study

Although we have performed a detailed genomic investigation of the intrahost evolution and genetic diversity during chronic infection, a potential limitation of our study is that we have characterized a single case. However, we have utilized other published case studies of chronic SARS-CoV-2 infection to contextualize our findings and understand commonalities and differences between infections. In this study, it was not feasible to disentangle the increasing iSNV frequency within lineages from changing frequency of the lineages in the sample, which could likely conflate the increasing diversity within the lineage and possibly make it less clear whether certain sites in the genome, such as those reaching fixation, possibly provide a selective advantage to the virus. Future studies should disentangle these effects using long-read sequencing to resolve haplotypes within the sample to accurately assign iSNVs to distinct lineages coexisting within the sample and perform additional tests to determine whether any mutations or phylogenetic branches are under significant selection pressure. Additionally, we did not compare the antibody neutralization susceptibility of different intrahost genotypes emerging during the chronic infection. Therefore, future studies of chronic infections, especially those utilizing prospectively collected samples, should include longitudinal and parallel samples to monitor several immune parameters such as antibody levels and immune cell composition as well as serum samples for neutralization assays to generate additional insights on the persistence and evolution of multiple genetically distinct genotypes in the same host. For this study, we did not have access to this additional information including human leukocyte antigen (HLA) haplotype data, which would have been valuable in evaluating the contribution of the host's immune system to the emergence of the observed genetic diversity of the viral population.

CONSORTIA

The members of the Yale SARS-CoV-2 Genomic Surveillance Initiative Team are Kendall Billig, Rebecca Earnest, Joseph R. Fauver, Chaney C. Kalinch, Nicholas Kerantzas, Tobias R. Koch, Bony De Kumar, Marie L. Landry, Isabel M. Ott, David Peaper, Irina R. Tikhonova, and Chantal B.F. Vogels.

STAR★METHODS

Detailed methods are provided in the online version of this paper and include the following:

- KEY RESOURCES TABLE
- RESOURCE AVAILABILITY
  - Lead contact
  - Materials availability
  - Data and code availability
- EXPERIMENTAL MODEL AND SUBJECT DETAILS
  - Ethics statement
- METHOD DETAILS
  - PCR testing and whole-genome sequencing
  - Primer ID sequencing using unique molecular identifiers
  - Testing for the infectious virus in nasopharyngeal swab samples
  - Clinical data
- QUANTIFICATION AND STATISTICAL ANALYSIS
  - Phylogenetic reconstruction and recombination analysis
  - Intrahost evolution and genetic diversity analysis
  - Statistical analysis and data visualization

SUPPLEMENTAL INFORMATION

Supplemental information can be found online at https://doi.org/10.1016/j.xcrm.2023.100943.

ACKNOWLEDGMENTS

We would like to thank the Yale New Haven Health COVID-19 testing enterprise for collecting and testing samples, the healthcare workers for supporting the patients, and the patients for contributing samples. We also thank J.T. McCrone, P. Jack, and S. Taylor for technical discussions about the methodologies. This work was supported by the Centers for Disease Control and Prevention (CDC) Broad Agency Announcement #75D30120C09570 (N.D.G.). This work was also supported by NIH award R01-AI140970 to R.I.S. This research received infrastructure support from the University of North Carolina (UNC) CFAR (P30-AI050410) and the UNC Lineberger Comprehensive Cancer Center (P30-CA016086). We acknowledge the support of the UNC High Throughput Sequencing Facility.

AUTHOR CONTRIBUTIONS

C. Chaguza, M.E.P., S.C.R., and N.D.G. conceived the study; S.C.R., D.F., W.S., and N.D.G. collected the clinical data and/or samples; M.I.B., A.M.H., and N.D.G. performed DNA extraction and sequencing library preparation; C. Chaguza, A.M.H., M.E.P., K.P., C. Castaldi, and N.D.G. performed the whole-genome sequencing and analysis; S.Z. and R.I.S. performed unique molecular ID sequencing (Primer ID); A.M.H. and M.A.P.-H. performed plaque assay experiments; C. Chaguza, M.E.P., A.M.H., and N.D.G. designed the analysis methods and analyzed the data; S.C.R. wrote the IRB protocol; C. Chaguza, M.E.P., A.M.H., S.C.R., and N.D.G. drafted the manuscript; N.D.G. secured funds and supervised the project; all authors reviewed and approved the manuscript.

DECLARATION OF INTERESTS

N.D.G. is a consultant for Tempus Labs and the National Basketball Association for work related to COVID-19 but is outside the submitted work. The University of North Carolina is pursuing intellectual property protection for Primer ID sequencing, and R.I.S. has received nominal royalties from licensing.

Received: July 1, 2022
Revised: October 12, 2022
Accepted: January 20, 2023
Published: January 27, 2023

REFERENCES

1. Dhar, M.S., Marwal, R., Vs, R., Ponnusamy, K., Jolly, B., Bhoyar, R.C., Sardana, V., Naushin, S., Rophina, M., Mellan, T.A., et al. (2021). Genomic characterization and epidemiology of an emerging SARS-CoV-2 variant in Delhi, India. Science *374*, 995–999. https://doi.org/10.1126/science.abj9932.
2. Tegally, H., Wilkinson, E., Giovanetti, M., Iranzadeh, A., Fonseca, V., Giandhari, J., Doolabh, D., Pillay, S., San, E.J., Msomi, N., et al. (2021). Detection of a SARS-CoV-2 variant of concern in South Africa. Nature *592*, 438–443. https://doi.org/10.1038/s41586-021-03402-9.
3. Volz, E., Mishra, S., Chand, M., Barrett, J.C., Johnson, R., Geidelberg, L., Hinsley, W.R., Laydon, D.J., Dabrera, G., O'Toole, Á., et al. (2021). Assessing transmissibility of SARS-CoV-2 lineage B.1.1.7 in England. Nature *593*, 266–269. https://doi.org/10.1038/s41586-021-03470-x.
4. Mlcochova, P., Kemp, S.A., Dhar, M.S., Papa, G., Meng, B., Ferreira, I.A.T.M., Datir, R., Collier, D.A., Albecka, A., Singh, S., et al. (2021). SARS-CoV-2 B.1.617.2 Delta variant replication and immune evasion. Nature *599*, 114–119. https://doi.org/10.1038/s41586-021-03944-y.
5. Takashita, E., Kinoshita, N., Yamayoshi, S., Sakai-Tagawa, Y., Fujisaki, S., Ito, M., Iwatsuki-Horimoto, K., Chiba, S., Halfmann, P., Nagai, H., et al. (2022). Efficacy of antibodies and antiviral drugs against Covid-19 Omicron variant. N. Engl. J. Med. *386*, 995–998. https://doi.org/10.1056/NEJMc2119407.
6. Andrews, N., Stowe, J., Kirsebom, F., Toffa, S., Rickeard, T., Gallagher, E., Gower, C., Kall, M., Groves, N., O'Connell, A.M., et al. (2022). Covid-19 vaccine effectiveness against the Omicron (B.1.1.529) variant. N. Engl. J. Med. *386*, 1532–1546. https://doi.org/10.1056/NEJMoa2119451.
7. Grubaugh, N.D., and Cobey, S. (2021). Of variants and vaccines. Cell *184*, 6222–6223. https://doi.org/10.1016/j.cell.2021.11.013.
8. Hill, V., Du Plessis, L., Peacock, T.P., Aggarwal, D., Colquhoun, R., Carabelli, A.M., Ellaby, N., Gallagher, E., Groves, N., Jackson, B., et al. (2022). The origins and molecular evolution of SARS-CoV-2 lineage B.1.1.7 in the UK. https://doi.org/10.1101/2022.03.08.481609.
9. Wu, F., Zhao, S., Yu, B., Chen, Y.M., Wang, W., Song, Z.G., Hu, Y., Tao, Z.W., Tian, J.H., Pei, Y.Y., et al. (2020). A new coronavirus associated with human respiratory disease in China. Nature *579*, 265–269. https://doi.org/10.1038/s41586-020-2008-3.
10. Viana, R., Moyo, S., Amoako, D.G., Tegally, H., Scheepers, C., Althaus, C.L., Anyaneji, U.J., Bester, P.A., Boni, M.F., Chand, M., et al. (2022). Rapid epidemic expansion of the SARS-CoV-2 Omicron variant in southern Africa. Nature *603*, 679–686. https://doi.org/10.1038/s41586-022-04411-y.
11. Naveca, F.G., Nascimento, V., de Souza, V.C., Corado, A.d.L., Nascimento, F., Silva, G., Costa, Á., Duarte, D., Pessoa, K., Mejía, M., et al. (2021). COVID-19 in Amazonas, Brazil, was driven by the persistence of endemic lineages and P.1 emergence. Nat. Med. *27*, 1230–1238. https://doi.org/10.1038/s41591-021-01378-7.
12. Gräf, T., Bello, G., Venas, T.M.M., Pereira, E.C., Paixão, A.C.D., Appolinario, L.R., Lopes, R.S., Mendonça, A.C.D.F., da Rocha, A.S.B., Motta, F.C., et al. (2021). Identification of a novel SARS-CoV-2 P.1 sub-lineage in Brazil provides new insights about the mechanisms of emergence of variants of concern. Virus Evol. *7*, veab091. https://doi.org/10.1093/ve/veab091.
13. Oude Munnink, B.B., Sikkema, R.S., Nieuwenhuijse, D.F., Molenaar, R.J., Munger, E., Molenkamp, R., van der Spek, A., Tolsma, P., Rietveld, A., Brouwer, M., et al. (2021). Transmission of SARS-CoV-2 on mink farms between humans and mink and back to humans. Science *371*, 172–177. https://doi.org/10.1126/science.abe5901.
14. Lythgoe, K.A., Hall, M., Ferretti, L., de Cesare, M., MacIntyre-Cockett, G., Trebes, A., Andersson, M., Otecko, N., Wise, E.L., Moore, N., et al. (2021). SARS-CoV-2 within-host diversity and transmission. Science *372*, eabg0821. https://doi.org/10.1126/science.abg0821.
15. Braun, K.M., Moreno, G.K., Wagner, C., Accola, M.A., Rehrauer, W.M., Baker, D.A., Koelle, K., O'Connor, D.H., Bedford, T., Friedrich, T.C., and Moncla, L.H. (2021). Acute SARS-CoV-2 infections harbor limited within-host diversity and transmit via tight transmission bottlenecks. PLoS Pathog. *17*, e1009849. https://doi.org/10.1371/journal.ppat.1009849.
16. Tay, J.H., Porter, A.F., Wirth, W., and Duchene, S. (2022). The emergence of SARS-CoV-2 variants of concern is driven by acceleration of the substitution rate. Mol. Biol. Evol. *39*, msac013. https://doi.org/10.1093/molbev/msac013.
17. Grenfell, B.T., Pybus, O.G., Gog, J.R., Wood, J.L.N., Daly, J.M., Mumford, J.A., and Holmes, E.C. (2004). Unifying the epidemiological and evolutionary dynamics of pathogens. Science *303*, 327–332. https://doi.org/10.1126/science.1090727.
18. Jackson, B., Boni, M.F., Bull, M.J., Colleran, A., Colquhoun, R.M., Darby, A.C., Haldenby, S., Hill, V., Lucaci, A., McCrone, J.T., et al. (2021). Generation and transmission of interlineage recombinants in the SARS-CoV-2 pandemic. Cell *184*, 5179–5188.e8. https://doi.org/10.1016/j.cell.2021.08.014.
19. Wilkinson, S.A.J., Richter, A., Casey, A., Osman, H., Mirza, J.D., Stockton, J., Quick, J., Ratcliffe, L., Sparks, N., Cumley, N., et al. (2022). Recurrent SARS-CoV-2 mutations in immunodeficient patients. https://doi.org/10.1101/2022.03.02.22271697.
20. Gonzalez-Reiche, A.S., Alshammary, H., Schaefer, S., Patel, G., Polanco, J., Amoako, A.A., Rooker, A., Cognigni, C., Floda, D., van de Guchte, A., et al. (2022). Intrahost evolution and forward transmission of a novel SARS-CoV-2 Omicron BA.1 subvariant. https://doi.org/10.1101/2022.05.25.22275533.

21. Cele, S., Karim, F., Lustig, G., San, J.E., Hermanus, T., Tegally, H., Snyman, J., Moyo-Gwete, T., Wilkinson, E., Bernstein, M., et al. (2022). SARS-CoV-2 prolonged infection during advanced HIV disease evolves extensive immune escape. Cell Host Microbe *30*, 154–162.e5. https://doi.org/10.1016/j.chom.2022.01.005.

22. Maponga, T.G., Jeffries, M., Tegally, H., Sutherland, A.D., Wilkinson, E., Lessells, R., Msomi, N., van Zyl, G., de Oliveira, T., and Preiser, W. (2022). Persistent SARS-CoV-2 infection with accumulation of mutations in a patient with poorly controlled HIV infection. SSRN Journal. https://doi.org/10.2139/ssrn.4014499.

23. Gandhi, S., Klein, J., Robertson, A.J., Peña-Hernández, M.A., Lin, M.J., Roychoudhury, P., Lu, P., Fournier, J., Ferguson, D., Mohamed Bakhash, S.A.K., et al. (2022). De novo emergence of a remdesivir resistance mutation during treatment of persistent SARS-CoV-2 infection in an immunocompromised patient: a case report. Nat. Commun. *13*, 1547. https://doi.org/10.1038/s41467-022-29104-y.

24. Weigang, S., Fuchs, J., Zimmer, G., Schnepf, D., Kern, L., Beer, J., Luxenburger, H., Ankerhold, J., Falcone, V., Kemming, J., et al. (2021). Within-host evolution of SARS-CoV-2 in an immunosuppressed COVID-19 patient as a source of immune escape variants. Nat. Commun. *12*, 6405. https://doi.org/10.1038/s41467-021-26602-3.

25. Avanzato, V.A., Matson, M.J., Seifert, S.N., Pryce, R., Williamson, B.N., Anzick, S.L., Barbian, K., Judson, S.D., Fischer, E.R., Martens, C., et al. (2020). Case study: prolonged infectious SARS-CoV-2 shedding from an asymptomatic immunocompromised individual with cancer. Cell *183*, 1901–1912.e9. https://doi.org/10.1016/j.cell.2020.10.049.

26. Kemp, S.A., Collier, D.A., Datir, R.P., Ferreira, I.A.T.M., Gayed, S., Jahun, A., Hosmillo, M., Rees-Spear, C., Mlcochova, P., Lumb, I.U., et al. (2021). SARS-CoV-2 evolution during treatment of chronic infection. Nature *592*, 277–282. https://doi.org/10.1038/s41586-021-03291-y.

27. Choi, B., Choudhary, M.C., Regan, J., Sparks, J.A., Padera, R.F., Qiu, X., Solomon, I.H., Kuo, H.H., Boucau, J., Bowman, K., et al. (2020). Persistence and evolution of SARS-CoV-2 in an immunocompromised host. N. Engl. J. Med. *383*, 2291–2293. https://doi.org/10.1056/NEJMc2031364.

28. Li, J., Zhang, L., Liu, B., and Song, D. (2020). Case report: viral shedding for 60 Days in a woman with COVID-19. Am. J. Trop. Med. Hyg. *102*, 1210–1213. https://doi.org/10.4269/ajtmh.20-0275.

29. Monrad, I., Sahlertz, S.R., Nielsen, S.S.F., Pedersen, L.Ø., Petersen, M.S., Kobel, C.M., Tarpgaard, I.H., Storgaard, M., Mortensen, K.L., Schleimann, M.H., et al. (2021). Persistent severe acute respiratory syndrome coronavirus 2 infection in immunocompromised host displaying treatment induced viral evolution. Open Forum Infect. Dis. *8*, ofab295. https://doi.org/10.1093/ofid/ofab295.

30. Wilkinson, S.A.J., Richter, A., Casey, A., Osman, H., Mirza, J.D., Stockton, J., Quick, J., Ratcliffe, L., Sparks, N., Cumley, N., et al. (2022). Recurrent SARS-CoV-2 mutations in immunodeficient patients. Virus Evol. *8*, veac050. https://doi.org/10.1093/ve/veac050.

31. Nussenblatt, V., Roder, A.E., Das, S., de Wit, E., Youn, J.H., Banakis, S., Mushegian, A., Mederos, C., Wang, W., Chung, M., et al. (2022). Yearlong COVID-19 infection reveals within-host evolution of SARS-CoV-2 in a patient with B-cell depletion. J. Infect. Dis. *225*, 1118–1123. https://doi.org/10.1093/infdis/jiab622.

32. Williamson, M.K., Hamilton, F., Hutchings, S., Pymont, H.M., Hackett, M., Arnold, D., Maskell, N.A., MacGowan, A., Albur, M., Jenkins, M., et al. (2021). Chronic SARS-CoV-2 infection and viral evolution in a hypogammaglobulinaemic individual. https://doi.org/10.1101/2021.05.31.21257591.

33. Truong, T.T., Ryutov, A., Pandey, U., Yee, R., Goldberg, L., Bhojwani, D., Aguayo-Hiraldo, P., Pinsky, B.A., Pekosz, A., Shen, L., et al. (2021). Increased viral variants in children and young adults with impaired humoral immunity and persistent SARS-CoV-2 infection: a consecutive case series. EBioMedicine *67*, 103355. https://doi.org/10.1016/j.ebiom.2021.103355.

34. Ko, K.K.K., Yingtaweesittikul, H., Tan, T.T., Wijaya, L., Cao, D.Y., Goh, S.S., Abdul Rahman, N.B., Chan, K.X.L., Tay, H.M., Sim, J.H.C., et al. (2022). Emergence of SARS-CoV-2 spike mutations during prolonged infection in immunocompromised hosts. Microbiol. Spectr. *10*, e0079122. https://doi.org/10.1128/spectrum.00791-22.

35. Borges, V., Isidro, J., Cunha, M., Cochicho, D., Martins, L., Banha, L., Figueiredo, M., Rebelo, L., Trindade, M.C., Duarte, S., et al. (2021). Long-term evolution of SARS-CoV-2 in an immunocompromised patient with non-Hodgkin lymphoma. mSphere *6*, e0024421. https://doi.org/10.1128/msphere.00244-21.

36. Hensley, M.K., Bain, W.G., Jacobs, J., Nambulli, S., Parikh, U., Cillo, A., Staines, B., Heaps, A., Sobolewski, M.D., Rennick, L.J., et al. (2021). Intractable coronavirus disease 2019 (COVID-19) and prolonged severe acute respiratory syndrome coronavirus 2 (SARS-CoV-2) replication in a chimeric antigen receptor-modified T-cell therapy recipient: a case study. Clin. Infect. Dis. *73*, e815–21. https://doi.org/10.1093/cid/ciab072.

37. McCarthy, K.R., Rennick, L.J., Nambulli, S., Robinson-McCarthy, L.R., Bain, W.G., Haidar, G., and Duprex, W.P. (2021). Recurrent deletions in the SARS-CoV-2 spike glycoprotein drive antibody escape. Science *371*, 1139–1142. https://doi.org/10.1126/science.abf6950.

38. Khatamzas, E., Rehn, A., Muenchhoff, M., Hellmuth, J., Gaitzsch, E., Weiglein, T., Georgi, E., Scherer, C., Stecher, S., Weigert, O., et al. (2021). Emergence of multiple SARS-CoV-2 mutations in an immunocompromised host. https://doi.org/10.1101/2021.01.10.20248871.

39. Halfmann, P.J., Minor, N.R., Haddock, L.A., Maddox, R., Moreno, G.K., Braun, K.M., Baker, D.A., Riemersa, K.K., Prasad, A., Alman, K.J., et al. (2022). Evolution of a globally unique SARS-CoV-2 Spike E484T monoclonal antibody escape mutation in a persistently infected, immunocompromised individual. https://doi.org/10.1101/2022.04.11.22272784.

40. Harari, S., Tahor, M., Rutsinsky, N., Meijer, S., Miller, D., Henig, O., Halutz, O., Levytskyi, K., Ben-Ami, R., Adler, A., et al. (2022). Drivers of adaptive evolution during chronic SARS-CoV-2 infections. https://doi.org/10.1101/2022.02.17.22270829.

41. Bradley, R.E., Ponsford, M.J., Scurr, M.J., Godkin, A., and Jolles, S.; Immunodeficiency Centre for Wales (2022). Persistent COVID-19 infection in Wiskott-Aldrich syndrome cleared following therapeutic vaccination: a case report. J. Clin. Immunol. *42*, 32–35. https://doi.org/10.1007/s10875-021-01158-5.

42. Shapira, G., Weiner, C., Abramovich, R.S., Gutwein, O., Rainy, N., Benveniste-Levkovitz, P., Gordon, E., Bar Chaim, A., and Shomron, N. (2022). SARS-CoV-2 evolution and evasion from multiple antibody treatments in a cancer patient. https://doi.org/10.1101/2022.06.25.22276445.

43. Delorey, T.M., Ziegler, C.G.K., Heimberg, G., Normand, R., Yang, Y., Segerstolpe, Å., Abbondanza, D., Fleming, S.J., Subramanian, A., Montoro, D.T., et al. (2021). COVID-19 tissue atlases reveal SARS-CoV-2 pathology and cellular targets. Nature *595*, 107–113. https://doi.org/10.1038/s41586-021-03570-8.

44. van Riel, D., Munster, V.J., de Wit, E., Rimmelzwaan, G.F., Fouchier, R.A.M., Osterhaus, A.D.M.E., and Kuiken, T. (2006). H5N1 virus attachment to lower respiratory tract. Science *312*, 399. https://doi.org/10.1126/science.1125548.

45. Shinya, K., Ebina, M., Yamada, S., Ono, M., Kasai, N., and Kawaoka, Y. (2006). Influenza virus receptors in the human airway. Nature *440*, 435–436. https://www.nature.com/articles/440435a.

46. Jorth, P., Staudinger, B.J., Wu, X., Hisert, K.B., Hayden, H., Garudathri, J., Harding, C.L., Radey, M.C., Rezayat, A., Bautista, G., et al. (2015). Regional isolation drives bacterial diversification within cystic fibrosis lungs. Cell Host Microbe *18*, 307–319. https://doi.org/10.1016/j.chom.2015.07.006.

47. Lieberman, T.D., Wilson, D., Misra, R., Xiong, L.L., Moodley, P., Cohen, T., and Kishony, R. (2016). Genomic diversity in autopsy samples reveals within-host dissemination of HIV-associated Mycobacterium tuberculosis. Nat. Med. *22*, 1470–1474. https://doi.org/10.1038/nm.4205.

48. Farjo, M., Koelle, K., Martin, M.A., Gibson, L.L., Walden, K.K.O., Rendon, G., Fields, C.J., Alnaji, F.G., Gallagher, N., Luo, C.H., et al. (2022). Within-host evolutionary dynamics and tissue compartmentalization during acute SARS-CoV-2 infection. https://doi.org/10.1101/2022.06.21.497047.

CellPress
OPEN ACCESS

49. Didelot, X., Walker, A.S., Peto, T.E., Crook, D.W., and Wilson, D.J. (2016). Within-host evolution of bacterial pathogens. Nat. Rev. Microbiol. *14*, 150–162. https://doi.org/10.1038/nrmicro.2015.13.

50. Fauver, J.R., Petrone, M.E., Hodcroft, E.B., Shioda, K., Ehrlich, H.Y., Watts, A.G., Vogels, C.B.F., Brito, A.F., Alpert, T., Muyombwe, A., et al. (2020). Coast-to-Coast spread of SARS-CoV-2 during the early epidemic in the United States. Cell *181*, 990–996.e5. https://doi.org/10.1016/j.cell.2020.04.021.

51. Aggarwal, D., Warne, B., Jahun, A., Hamilton, W., Fieldman, T., Plessis, L., Hill, V., Blane, B., Watkins, E., Wright, E., et al. (2022). Genomic epidemiology of SARS-CoV-2 in a UK university identifies dynamics of transmission. https://doi.org/10.21203/rs.3.rs-520627/v1.

52. Nadeau, S.A., Vaughan, T.G., Scire, J., Huisman, J.S., and Stadler, T. (2021). The origin and early spread of SARS-CoV-2 in Europe. Proc. Natl. Acad. Sci. USA *118*. https://doi.org/10.1073/pnas.2012008118.

53. Duchene, S., Featherstone, L., Haritopoulou-Sinanidou, M., Rambaut, A., Lemey, P., and Baele, G. (2020). Temporal signal and the phylodynamic threshold of SARS-CoV-2. Virus Evol. *6*, veaa061. https://doi.org/10.1093/ve/veaa061.

54. Zhou, S., Hill, C.S., Clark, M.U., Sheahan, T.P., Baric, R., and Swanstrom, R. (2021). Primer ID next-generation sequencing for the analysis of a Broad spectrum antiviral induced transition mutations and errors rates in a coronavirus genome. Bio. Protoc. *11*, e3938. https://doi.org/10.21769/BioProtoc.3938.

55. Zhou, S., Jones, C., Mieczkowski, P., and Swanstrom, R. (2015). Primer ID validates template sampling depth and greatly reduces the error rate of next-generation sequencing of HIV-1 genomic RNA populations. J. Virol. *89*, 8540–8555. https://doi.org/10.1128/JVI.00522-15.

56. Sagulenko, P., Puller, V., and Neher, R.A. (2018). TreeTime: maximum-likelihood phylodynamic analysis. Virus Evol. *4*, vex042. https://doi.org/10.1093/ve/vex042.

57. Tonkin-Hill, G., Martincorena, I., Amato, R., Lawson, A.R.J., Gerstung, M., Johnston, I., Jackson, D.K., Park, N., Lensing, S.V., Quail, M.A., et al. (2021). Patterns of within-host genetic diversity in SARS-CoV-2. Elife *10*, e66857. https://doi.org/10.7554/eLife.66857.

58. Popa, A., Genger, J.W., Nicholson, M.D., Penz, T., Schmid, D., Aberle, S.W., Agerer, B., Lercher, A., Endler, L., Colaço, H., et al. (2020). Genomic epidemiology of superspreading events in Austria reveals mutational dynamics and transmission properties of SARS-CoV-2. Sci. Transl. Med. *12*, eabe2555. https://doi.org/10.1126/scitranslmed.abe2555.

59. Simmonds, P. (2020). Rampant C→U Hypermutation in the genomes of SARS-CoV-2 and other coronaviruses: causes and consequences for their short- and long-term evolutionary trajectories. mSphere *5*, e00408-20. https://doi.org/10.1128/mSphere.00408-20.

60. Huang, K., Zhang, Y., Hui, X., Zhao, Y., Gong, W., Wang, T., Zhang, S., Yang, Y., Deng, F., Zhang, Q., et al. (2021). Q493K and Q498H substitutions in Spike promote adaptation of SARS-CoV-2 in mice. EBioMedicine *67*, 103381. https://doi.org/10.1016/j.ebiom.2021.103381.

61. Focosi, D., Novazzi, F., Genoni, A., Dentali, F., Gasperina, D.D., Baj, A., and Maggi, F. (2021). Emergence of SARS-COV-2 spike protein escape mutation Q493R after treatment for COVID-19. Emerg. Infect. Dis. *27*, 2728–2731. https://doi.org/10.3201/eid2710.211538.

62. Starr, T.N., Greaney, A.J., Addetia, A., Hannon, W.W., Choudhary, M.C., Dingens, A.S., Li, J.Z., and Bloom, J.D. (2021). Prospective mapping of viral mutations that escape antibodies used to treat COVID-19. Science *371*, 850–854. https://doi.org/10.1126/science.abf9302.

63. Weisblum, Y., Schmidt, F., Zhang, F., DaSilva, J., Poston, D., Lorenzi, J.C., Muecksch, F., Rutkowska, M., Hoffmann, H.H., Michailidis, E., et al. (2020). Escape from neutralizing antibodies by SARS-CoV-2 spike protein variants. Elife *9*, e61312. https://doi.org/10.7554/eLife.61312.

64. Liu, L., Iketani, S., Guo, Y., Chan, J.F.W., Wang, M., Liu, L., Luo, Y., Chu, H., Huang, Y., Nair, M.S., et al. (2022). Striking antibody evasion manifested by the Omicron variant of SARS-CoV-2. Nature *602*, 676–681. https://doi.org/10.1038/s41586-021-04388-0.

65. Jangra, S., Ye, C., Rathnasinghe, R., Stadlbauer, D., Personalized Virology Initiative study group; Krammer, F., Simon, V., Martinez-Sobrido, L., García-Sastre, A., and Schotsaert, M. (2021). SARS-CoV-2 spike E484K mutation reduces antibody neutralisation. Lancet. Microbe *2*, e283–e284. https://doi.org/10.1016/S2666-5247(21)00068-9.

66. Wertheim, J.O., Wang, J.C., Leelawong, M., Martin, D.P., Havens, J.L., Chowdhury, M.A., Pekar, J., Amin, H., Arroyo, A., Awandare, G.A., et al. (2022). Capturing intrahost recombination of SARS-CoV-2 during superinfection with Alpha and Epsilon variants in New York City. https://doi.org/10.1101/2022.01.18.22269300.

67. Choudhary, M.C., Crain, C.R., Qiu, X., Hanage, W., and Li, J.Z. (2022). Severe acute respiratory syndrome coronavirus 2 (SARS-CoV-2) sequence characteristics of coronavirus disease 2019 (COVID-19) persistence and reinfection. Clin. Infect. Dis. *74*, 237–245. https://doi.org/10.1093/cid/ciab380.

68. Mallapaty, S. (2022). Where did Omicron come from? Three key theories. Nature *602*, 26–28. https://doi.org/10.1038/d41586-022-00215-2.

69. Kupferschmidt, K. (2021). Where did “weird” Omicron come from? Science *374*, 1179. https://doi.org/10.1126/science.acx9738.

70. Lemieux, J.E., and Luban, J. (2022). Consulting the oracle of SARS-CoV-2 infection. J. Infect. Dis. *225*, 1115–1117. https://doi.org/10.1093/infdis/jiab623.

71. Callaway, E. (2022). How months-long COVID infections could seed dangerous new variants. Nature *606*, 452–455. https://doi.org/10.1038/d41586-022-01613-2.

72. Viberg, L.T., Sarovich, D.S., Kidd, T.J., Geake, J.B., Bell, S.C., Currie, B.J., and Price, E.P. (2017). Within-host evolution of during chronic infection of seven australasian cystic fibrosis patients. mBio *8*, e003566-17. https://doi.org/10.1128/mBio.00356-17.

73. Meng, B., Abdullahi, A., Ferreira, I.A.T.M., Goonawardane, N., Saito, A., Kimura, I., Yamasoba, D., Gerber, P.P., Fatihi, S., Rathore, S., et al. (2022). Altered TMPRSS2 usage by SARS-CoV-2 Omicron impacts infectivity and fusogenicity. Nature *603*, 706–714. https://doi.org/10.1038/s41586-022-04474-x.

74. Harvey, W.T., Carabelli, A.M., Jackson, B., Gupta, R.K., Thomson, E.C., Harrison, E.M., Ludden, C., Reeve, R., Rambaut, A., et al.; COVID-19 Genomics UK COG-UK Consortium (2021). SARS-CoV-2 variants, spike mutations and immune escape. Nat. Rev. Microbiol. *19*, 409–424. https://doi.org/10.1038/s41579-021-00573-0.

75. Greaney, A.J., Loes, A.N., Crawford, K.H.D., Starr, T.N., Malone, K.D., Chu, H.Y., and Bloom, J.D. (2021). Comprehensive mapping of mutations in the SARS-CoV-2 receptor-binding domain that affect recognition by polyclonal human plasma antibodies. Cell Host Microbe *29*, 463–476.e6. https://doi.org/10.1016/j.chom.2021.02.003.

76. Starr, T.N., Greaney, A.J., Dingens, A.S., and Bloom, J.D. (2021). Complete map of SARS-CoV-2 RBD mutations that escape the monoclonal antibody LY-CoV555 and its cocktail with LY-CoV016. Cell Rep. Med. *2*, 100255. https://doi.org/10.1016/j.xcrm.2021.100255.

77. Peacock, T.P., Penrice-Randal, R., Hiscox, J.A., and Barclay, W.S. (2021). SARS-CoV-2 one year on: evidence for ongoing viral adaptation. J. Gen. Virol. *102*, 001584. https://doi.org/10.1099/jgv.0.001584.

78. Dennehy, J.J., Gupta, R.K., Hanage, W.P., Johnson, M.C., and Peacock, T.P. (2022). Where is the next SARS-CoV-2 variant of concern? Lancet *399*, 1938–1939. https://doi.org/10.1016/S0140-6736(22)00743-7.

79. Collie, S., Champion, J., Moultrie, H., Bekker, L.G., and Gray, G. (2022). Effectiveness of BNT162b2 vaccine against Omicron variant in South Africa. N. Engl. J. Med. *386*, 494–496. https://doi.org/10.1056/NEJMc2119270.

80. Burton, D.R., and Topol, E.J. (2021). Variant-proof vaccines — invest now for the next pandemic. Nature *590*, 386–388. https://doi.org/10.1038/d41586-021-00340-4.

81. Morens, D.M., Taubenberger, J.K., and Fauci, A.S. (2022). Universal coronavirus vaccines — an urgent need. N. Engl. J. Med. *386*, 297–299. https://doi.org/10.1056/nejmp2118468.

82. Vogels, C.B.F., Brito, A.F., Wyllie, A.L., Fauver, J.R., Ott, I.M., Kalinich, C.C., Petrone, M.E., Casanovas-Massana, A., Catherine Muenker, M., Moore, A.J., et al. (2020). Analytical sensitivity and efficiency comparisons of SARS-CoV-2 RT-qPCR primer-probe sets. Nat. Microbiol. *5*, 1299–1305. https://doi.org/10.1038/s41564-020-0761-6.

83. Vogels, C.B.F., Breban, M.I., Ott, I.M., Alpert, T., Petrone, M.E., Watkins, A.E., Kalinich, C.C., Earnest, R., Rothman, J.E., Goes de Jesus, J., et al. (2021). Multiplex qPCR discriminates variants of concern to enhance global surveillance of SARS-CoV-2. PLoS Biol. *19*, e3001236. https://doi.org/10.1371/journal.pbio.3001236.

84. Li, H. (2013). Aligning sequence reads, clone sequences and assembly contigs with BWA-MEM. Preprint at arXiv. http://arxiv.org/abs/1303.3997.

85. Grubaugh, N.D., Gangavarapu, K., Quick, J., Matteson, N.L., De Jesus, J.G., Main, B.J., Tan, A.L., Paul, L.M., Brackney, D.E., Grewal, S., et al. (2019). An amplicon-based sequencing framework for accurately measuring intrahost virus diversity using PrimalSeq and iVar. Genome Biol. *20*, 8. https://doi.org/10.1186/s13059-018-1618-7.

86. Li, H., Handsaker, B., Wysoker, A., Fennell, T., Ruan, J., Homer, N., Marth, G., Abecasis, G., and Durbin, R.; 1000 Genome Project Data Processing Subgroup (2009). The sequence alignment/map format and SAMtools. Bioinformatics *25*, 2078–2079. https://doi.org/10.1093/bioinformatics/btp352.

87. O'Toole, Á., Scher, E., Underwood, A., Jackson, B., Hill, V., McCrone, J.T., Colquhoun, R., Ruis, C., Abu-Dahab, K., Taylor, B., et al. (2021). Assignment of epidemiological lineages in an emerging pandemic using the Pangolin tool. Virus Evol. *7*, veab064. https://doi.org/10.1093/ve/veab064.

88. Rambaut, A., Holmes, E.C., O'Toole, Á., Hill, V., McCrone, J.T., Ruis, C., du Plessis, L., and Pybus, O.G. (2020). A dynamic nomenclature proposal for SARS-CoV-2 lineages to assist genomic epidemiology. Nat. Microbiol. *5*, 1403–1407. https://doi.org/10.1038/s41564-020-0770-5.

89. Danecek, P., Auton, A., Abecasis, G., Albers, C.A., Banks, E., DePristo, M.A., Handsaker, R.E., Lunter, G., Marth, G.T., Sherry, S.T., et al. (2011). The variant call format and VCFtools. Bioinformatics *27*, 2156–2158. https://doi.org/10.1093/bioinformatics/btr330.

90. Li, H. (2011). A statistical framework for SNP calling, mutation discovery, association mapping and population genetical parameter estimation from sequencing data. Bioinformatics *27*, 2987–2993. https://doi.org/10.1093/bioinformatics/btr509.

91. Hadfield, J., Megill, C., Bell, S.M., Huddleston, J., Potter, B., Callender, C., Sagulenko, P., Bedford, T., and Neher, R.A. (2018). Nextstrain: real-time tracking of pathogen evolution. Bioinformatics *34*, 4121–4123. https://doi.org/10.1093/bioinformatics/bty407.

92. Huddleston, J., Hadfield, J., Sibley, T.R., Lee, J., Fay, K., Ilcisin, M., Harkins, E., Bedford, T., Neher, R.A., and Hodcroft, E.B. (2021). Augur: a bioinformatics toolkit for phylogenetic analyses of human pathogens. J. Open Source Softw. *6*, 2906. https://doi.org/10.21105/joss.02906.

93. Nguyen, L.T., Schmidt, H.A., von Haeseler, A., and Minh, B.Q. (2015). IQ-TREE: a fast and effective stochastic algorithm for estimating maximum-likelihood phylogenies. Mol. Biol. Evol. *32*, 268–274. https://doi.org/10.1093/molbev/msu300.

94. Shu, Y., and McCauley, J. (2017). GISAID: global initiative on sharing all influenza data - from vision to reality. Euro Surveill. *22*, 30494. https://doi.org/10.2807/1560-7917.ES.2017.22.13.30494.

95. Paradis, E., Claude, J., and Strimmer, K. (2004). APE: analyses of phylogenetics and evolution in R language. Bioinformatics *20*, 289–290. https://doi.org/10.1093/bioinformatics/btg412.

96. Revell, L.J. (2012). phytools: an R package for phylogenetic comparative biology (and other things). Methods Ecol. Evol. *3*, 217–223. https://doi.org/10.1111/j.2041-210x.2011.00169.x.

97. Lam, H.M., Ratmann, O., and Boni, M.F. (2018). Improved algorithmic complexity for the 3SEQ recombination detection algorithm. Mol. Biol. Evol. *35*, 247–251. https://doi.org/10.1093/molbev/msx263.

98. Quinlan, A.R., and Hall, I.M. (2010). BEDTools: a flexible suite of utilities for comparing genomic features. Bioinformatics *26*, 841–842. https://doi.org/10.1093/bioinformatics/btq033.

STAR★METHODS

KEY RESOURCES TABLE

REAGENT or RESOURCE	SOURCE	IDENTIFIER
**Bacterial and virus strains**		
SARS-CoV-2 samples	Yale New Haven Hospital; Yale School of Public Health	https://www.ynhh.org/; https://ysph.yale.edu/
**Biological samples**		
SARS-CoV-2 samples	Yale New Haven Hospital; Yale School of Public Health	https://www.ynhh.org/; https://ysph.yale.edu/
**Critical commercial assays**		
MagMAX viral/pathogen nucleic acid isolation kit	Thermo Fisher Scientific, Waltham, MA, United States	A48383
Luna universal probe 1-Step RT-qPCR kit	New England Biolabs	NEB #E3005
Illumina COVIDSeq Test RUO version	Illumina	20049393, 20051772, 20051773
ARTIC primers v.4.1	Integrated DNA Technologies	#10011442
Transmembrane protease serine 2 (TMPRSS2)-ACE2-VeroE6 kidney epithelial cells	American Type Culture Collection (ATCC)	https://www.beiresources.org/Catalog/cellBanks/NR-54970.aspx
Dulbecco’s Modified Eagle’s Medium (DMEM)	Gibco	11965092
Minimum Essential Medium (MEM)	Gibco	31985062
Qubit High Sensitivity dsDNA kit	Life Technologies	Q32854
**Deposited data**		
Sequenced SARS-CoV-2 genomes	GISAID and Sequence Read Archive	Accession numbers for each genome are provided in Table S2.
**Software and algorithms**		
R	CRAN	https://www.R-project.org/
APE	CRAN	https://cran.r-project.org/web/packages/ape/
Phytools	CRAN	https://cran.r-project.org/web/packages/phytools/
Augur	GitHub	https://github.com/nextstrain/augur
Treetime	GitHub	https://github.com/neherlab/treetime
Auspice	Auspice	https://auspice.us/
3SEQ	GitHub	https://github.com/olli0601/3SEQ
Picard	GitHub	https://github.com/broadinstitute/picard
VCFTools	Sourceforge	http://vcftools.sourceforge.net/
SAMtools	GitHub	https://samtools.github.io/
iVar	GitHub	https://github.com/andersen-lab/ivar
GISAID	GISAID	https://gisaid.org/
BWA MEM	GitHub	https://github.com/lh3/bwa
Pangolin	GitHub	https://github.com/cov-lineages/pangolin
bcl2fastq	Illumina	https://support.illumina.com/downloads/bcl2fastq-conversion-software-v2-20.html
TCS pipeline	TCS pipeline	https://www.primer-id.org/tcs
Bcftools	GitHub	https://github.com/samtools/bcftools
vcf-annotator	GitHub	https://github.com/rpetit3/vcf-annotator
BEDTools	University of Utah	https://bedtools.readthedocs.io/en/latest/
IQ-TREE	GitHub	https://github.com/Cibiv/IQ-TREE
R	CRAN	https://cran.r-project.org/
Scripts	GitHub	https://github.com/grubaughlab/2022_paper_chronic_infection
iSNV data (Data S1)	Mendeley Data	Additional Supplemental Items are available from Mendeley Data at https://doi.org/10.17632/tvbt76bnbf.1

RESOURCE AVAILABILITY

Lead contact

Further information and requests for data, resources, and reagents should be directed to and will be fulfilled by the lead contact, Nathan D. Grubaugh (nathan.grubaugh@yale.edu).

Materials availability

This study did not generate new unique reagents.

Data and code availability

- The accession numbers for individual isolates are provided in the supplementary material Table S1.
- A summary of the SARS-CoV-2 samples is available in Data S1. All other data and code are available at https://github.com/grubaughlab/2022_paper_chronic_infection.
- All other data supporting the findings of this study are available within the paper and its supplemental information files. Any additional information required is available from the lead contact upon request.

EXPERIMENTAL MODEL AND SUBJECT DETAILS

Ethics statement

This study was approved by the Yale University Human Research Protection Program Institutional Review Board (IRB Protocol ID: 2000031415). Informed consent was obtained from the participant to take part in the study and to have the results of this work published. The coded numbers presented in the tables and figures are not identifiable to the patient.

METHOD DETAILS

PCR testing and whole-genome sequencing

Nasal swabs collected from the anterior nares or nasopharynx of confirmed SARS-CoV-2 positive individuals were routinely tested by the Yale New Haven Hospital COVID-19 and Clinical Virology Laboratories. We received remnant samples that were used for diagnostic testing. We used the MagMAX viral/pathogen nucleic acid isolation kit to extract nucleic acid from 300 μL of the collected sample by eluting in 75 μL of the elution buffer. We then extracted nucleic acid and tested it for SARS-CoV-2 RNA using a "research use only" (RUO) RT-qPCR assay using the CDC nucleocapsid gene target (N1) primer and probe set.[82] We converted the resulting N1 RT-PCR Ct values into SARS-CoV-2 RNA copies using a standard curve.[83]

We used the Illumina COVIDSeq Test RUO version to sequence samples with N1 PCT Ct values $\leq$ 35. We used ARTIC V3, V4, and V4.1 primer schemes for amplicon generation (https://github.com/artic-network/artic-ncov2019/tree/master/primer_schemes/nCoV-2019). We used a slightly modified sequencing protocol involving lowering the annealing temperature to 63°C when generating the amplicons and shortening the tagmentation step to 3 min. We pooled and cleaned the final libraries before DNA quantification using the Qubit High Sensitivity dsDNA kit (Life Technologies). The generated libraries were deep-sequenced using 2 × 150 bp paired-end reads on an Illumina NovaSeq at the Yale Center for Genome Analysis. At least one million paired-end reads were generated for each sample. We ensured that contamination would be flagged by including three negative controls (water added at RNA extraction, PCR, and library preparation) with every sequencing batch. We ensure that no or <100 SARS-CoV-2 reads are generated in each control to proceed with using the results. In general, we see high-quality, high-coverage sequences generated from samples up to Ct 35 which is above the Ct values for the samples used in this study. Furthermore, the samples presented here were sequenced over several batches following the time of swab collection, rendering a systematic error or batch effect unlikely.

The sequencing data were demultiplexed and processed, including converting base call (BCL) to FASTQ formats and trimmer adapter sequences, using Illumina bcl2fastq pipeline (v2.20.0). To generate consensus SARS-CoV-2 whole genomes, we aligned the reads to the ancestral SARS-CoV-2 reference genome (GenBank accessions: MN908937.3 or NC_045512.2) using BWA-MEM (version 0.7.15)[84] to generate indexed and sorted binary alignment map (BAM) files. We trimmed adaptors, masked primers and generated consensus base calls for the BAM files based on simple majority >60% base frequency using iVar (version 1.3.1)[85] and SAMtools (version 1.7).[86] We defined ambiguous base calls as nucleotide sites containing <20 unique mapped reads. To validate the sequencing runs, we sequenced negative controls, and in all cases consisted of >99% sites with Ns. We selected sequences containing >70% of non-N base calls for submission to GISAID. We assigned the SARS-CoV-2 lineages using Pangolin (version 3.1.17).[87,88]

Primer ID sequencing using unique molecular identifiers

UMI-guided deep sequencing was done using the previously published Primer ID next-generation sequencing protocol to sequence the SARS-CoV-2 viral genomes extracted from the specimens.[54,55] We used two sets of multiplexed UMI-tagged primers targeting SARS-CoV-2 ORF1ab (nsp12) and the spike gene. The cDNA and first-round PCR primers are provided in Table S4. After two rounds

of PCR amplification, purified and pooled libraries were deep-sequenced using MiSeq 300 base paired-end sequencing. Sequencing data were first processed using the Illumina bcl2fastq pipeline to convert BCL to FASTQ and trimmer adapters (v2.20.0), followed by the TCS pipeline (v2.5.0) (https://www.primer-id.org/tcs) to de-multiplex for sequencing regions, construct template consensus sequences (TCS). We used BWA-MEM (version 0.7.15)[84] to map the TCSs against the reconstructed ancestral B.1.517 sequence for the chronic infection generated from the phylogeny of the chronic infection genomes and annotated using the ancestral SARS-CoV-2 reference genome (GenBank: MN908937.3), bcftools (version 1.11–99-g5105724)[89,90] to generate variant calls, calculate iSNV frequency and merge the variant files, and vcf-annotator (version 0.7) (https://github.com/rpetit3/vcf-annotator) to annotate the merged variants.

Testing for the infectious virus in nasopharyngeal swab samples

To determine if the samples that test positive for viral RNA also contain infectious virus, we tested whether cell lines can be infected through nasopharyngeal swab material. For this, we chose twelve samples (40%) collected throughout the course of infection and available from the biorepository. For this, transmembrane protease serine 2 (TMPRSS2)-ACE2-VeroE6 kidney epithelial cells were cultured in Dulbecco's Modified Eagle's Medium (DMEM) supplemented with 1% sodium pyruvate (NEAA) and 10% Fetal bovine serum (FBS) at 37°C and 5% CO_2. The cell line was obtained from the American Type Culture Collection (ATCC) and tested negative for Mycoplasma contamination. Briefly, 250 μL of serial fold dilutions of sample material obtained from nasopharyngeal swabs in a viral transport medium were used to infect TMPRSS2-ACE2-Vero E6 cells for 1 h at 37°C for adsorption. We overlaid the cells with Minimum Essential Medium (MEM) supplemented with $NaHCO_3$, 4% Fetal Bovine Serum (FBS) and 0.6% Avicel RC-581. We resolved the plaques at 72 h post-infection by fixing them in 10% formaldehyde for 30 min, followed by 0.5% crystal violet in 20% ethanol staining. We then rinsed the plates in water and assessed the presence or absence of plaques. All experiments were carried out in a biosafety level 3 and biocontainment (BSL3) laboratory with approval from the Yale Environmental Health and Safety (EHS) office.

Clinical data

Information on clinical history and treatment was obtained from Yale New Haven Hospital. Longitudinal measurements of immune parameters (IgG levels, lymphocyte and T cell counts) were taken from chart review and obtained by standard clinical operation procedures.

QUANTIFICATION AND STATISTICAL ANALYSIS

Phylogenetic reconstruction and recombination analysis

For the phylogenetic analysis, we masked the sites in the 5′ (position 1 to 265) and 3′ (position 29,675 to 29,903) genomic regions, which are typically poorly sequenced and are known to bias the phylogeny. To understand the genetic relationship of the consensus SARS-CoV-2 genomes from the chronic infection and other WHO-designated SARS-CoV-2 variants (https://www.who.int/activities/tracking-SARS-CoV-2-variants), we constructed phylogenetic trees with branches resolved by time and genetic divergence, i.e., number of mutations, using the Nextstrain pipeline (version 3.0.3).[91] We used Nextalign (version 1.10.2) (https://github.com/neherlab/nextalign) and Augur (version 11.1.2),[92] implemented in the Nextstrain pipeline, to filter out the genomes based on sampling dates, construct maximum likelihood phylogenies with the generalized time-reversible (GTR) model using IQ-TREE (version 2.0.3),[93] refine and reconstruct mutations on the phylogeny, and estimate the effective population size (N_e). The last was based on the Coalescent Bayesian Skyline model using Treetime (version 0.8.1).[56] Finally, interactive visualization was undertaken using Auspice (version 2.23.0) (https://auspice.us/).[91]

For other variants, we randomly selected up to three contextual SARS-CoV-2 genomes per month per lineage (Pangolin) from the GISAID database[94] using dplyr (https://github.com/tidyverse/dplyr), and phylogenies generated using the same approach. We processed and visualized phylogenetic trees, including calculating root-to-tip distances, using ape (version 5.6.2)[95] and phytools (version 0.7.70).[96] We generated plots showing the location of mutations in the nucleotide sequence alignment using snipit (https://github.com/aineniamh/snipit).

To test for potential recombination, we used 3SEQ (version 1.7)[97] to check for potential recombination, first amongst the genomes from the chronic infection and also in comparison with randomly selected genomes belonging to other SARS-CoV-2 variants detected in Connecticut, USA, over the course of the chronic infection.

Intrahost evolution and genetic diversity analysis

To investigate the intrahost evolution and genetic diversity during chronic infection, we first used 'MarkDuplicates' in Picard (version 2.18.7) to identify duplicate reads in the BAM files of each sample (http://broadinstitute.github.io/picard/). We calculated the per-base sequencing depth using genomecov option in BedTools (version 2.30.0).[98] The bcftools (version 1.11–99-g5105724)[89,90] were used to generate variant calls for each sample using the reconstructed ancestral sequences for the chronic infection samples using the 'ancestral' option in the Augur pipeline (version 11.1.2),[92] which uses Treetime (version 0.8.1).[56] We specified a maximum depth of 1,000,000 with a minimum of 50 mapped reads per nucleotide site to infer variant calls. We used bcftools to calculate iSNV frequencies per sample and merge variant call files for different samples for annotation with vcf-annotator (version 0.7)

(https://github.com/rpetit3/vcf-annotator) using the reconstructed ancestral sequence generated from the phylogeny of the chronic infection genomes and annotated using the ancestral SARS-CoV-2 reference genome. We compared the commonness of iSNVs across different samples based on the variant type (intergenic, synonymous, and non-synonymous) and gene using the unpaired two-sample Wilcoxon test or Wilcoxon rank-sum test. We analyzed and visualized the presence and absence of mutations and their dynamics using R (version 4.0.3).

Statistical analysis and data visualization

All the statistical analyses and data visualizations were done using R (version 4.0.3) (R Core Team, https://www.R-project.org/).

Cell Reports Medicine, Volume *4*

Supplemental information

Accelerated SARS-CoV-2 intrahost evolution leading to distinct genotypes during chronic infection

Chrispin Chaguza, Anne M. Hahn, Mary E. Petrone, Shuntai Zhou, David Ferguson, Mallery I. Breban, Kien Pham, Mario A. Peña-Hernández, Christopher Castaldi, Verity Hill, Yale SARS-CoV-2 Genomic Surveillance Initiative, Wade Schulz, Ronald I. Swanstrom, Scott C. Roberts, and Nathan D. Grubaugh

Supplemental information

Accelerated SARS-CoV-2 intrahost evolution leading to distinct genotypes during chronic infection

Chrispin Chaguza, Anne M. Hahn, Mary E. Petrone, Shuntai Zhou, David Ferguson, Mallery I. Breban, Kien Pham, Mario A. Peña-Hernández, Christopher Castaldi, Verity Hill, Yale SARS-CoV-2 Genomic Surveillance Initiative,

Table S1. Summary of chronic SARS-CoV-2 lineage B.1.517 infection samples included in this study. Related to Figure 2.

Sequence ID	GISAID accession	Days since first positive RT-PCR test	RT-PCR Ct value	Virus copies per mL	Infectious virus	Primer ID sequencing	Dominant intrahost genotype	Primers
hCoV-19/USA/CT-Yale-12056/2021	EPI_ISL_10548915	79	20.5	3.01×10^{08}	Yes	Yes	1	NEB V3
hCoV-19/USA/CT-Yale-12057/2021	EPI_ISL_10548916	89	22.1	1.13×10^{08}	Not tested	Not done	1	NEB V3
hCoV-19/USA/CT-Yale-12058/2021	EPI_ISL_10548917	97	15.6	6.04×10^{09}	Yes	Yes	1	NEB V3
hCoV-19/USA/CT-Yale-12059/2021	EPI_ISL_10548918	104	25.2	1.69×10^{07}	No tested	Not done	1	NEB V3
hCoV-19/USA/CT-Yale-12060/2021	EPI_ISL_10548919	135	24.4	2.76×10^{07}	Not tested	Yes	1	NEB V3
hCoV-19/USA/CT-Yale-4087/2021	EPI_ISL_2035047	149	23.2	5.76×10^{07}	Yes	Yes	1	Illumina V3
hCoV-19/USA/CT-Yale-12061/2021	EPI_ISL_10548920	162	25.9	1.10×10^{07}	Not tested	Not done	1	NEB V3
hCoV-19/USA/CT-Yale-12062/2021	EPI_ISL_10548921	184	28.5	2.25×10^{06}	Not tested	Not done	1	NEB V3
hCoV-19/USA/CT-Yale-5581/2021	EPI_ISL_2716246	192	23.5	4.80×10^{07}	Not tested	Yes	1	Illumina V3
hCoV-19/USA/CT-Yale-5673/2021	EPI_ISL_2776212	205	21.5	1.63×10^{08}	Yes	Yes	1	Illumina V3
hCoV-19/USA/CT-Yale-5792/2021	EPI_ISL_2860316	212	29	1.66×10^{06}	Not tested	Yes	1	NEB V3
hCoV-19/USA/CT-Yale-12063/2021	EPI_ISL_10548922	219	27.2	4.98×10^{06}	Not tested	Not done	1	NEB V3
hCoV-19/USA/CT-Yale-6136/2021	EPI_ISL_3133023	233	22.3	1.00×10^{08}	Not tested	Yes	1	NEB V3
hCoV-19/USA/CT-Yale-6819/2021	EPI_ISL_3370176	247	20.9	2.35×10^{08}	Not tested	Yes	1	Illumina V3
hCoV-19/USA/CT-Yale-9391/2021	EPI_ISL_4198270	281	17.6	1.77×10^{09}	Yes	Yes	2	Illumina V3
hCoV-19/USA/CT-Yale-9977/2021	EPI_ISL_4576991	291	23.53	4.71×10^{07}	Not tested	Yes	2	Illumina V3
hCoV-19/USA/CT-Yale-10101R/2021	EPI_ISL_10548912	296	22.11	1.12×10^{08}	Not tested	Not done	1	NEB V3

hCoV-19/USA/CT-Yale-10960/2021	EPI_ISL_10548913	310	29.9	9.54×10^{05}	Yes	Not done	2	NEB V3
hCoV-19/USA/CT-Yale-11558/2021	EPI_ISL_5395558	317	24.6	2.45×10^{07}	Not tested	Yes	2	NEB V3
hCoV-19/USA/CT-Yale-11887/2021	EPI_ISL_5639913	325	29.1	1.56×10^{06}	Yes	Not done	2	NEB V3
hCoV-19/USA/CT-Yale-12124/2021	EPI_ISL_5865553	332	25.4	1.50×10^{07}	Not tested	Yes	2	NEB V3
hCoV-19/USA/CT-Yale-13443/2021	EPI_ISL_7361483	347	28.3	2.54×10^{06}	Yes	Yes	1	IDT V4
hCoV-19/USA/CT-Yale-13444/2021	EPI_ISL_7361527	353	32.5	1.94×10^{05}	Not tested	Not done	1	IDT V4
hCoV-19/USA/CT-Yale-14026/2021	EPI_ISL_7980711	360	26.3	8.64×10^{06}	Not tested	Yes	2	IDT V4.1
hCoV-19/USA/CT-Yale-15439/2021	EPI_ISL_8563219	381	21.2	1.96×10^{08}	Yes	Yes	1	IDT V4
hCoV-19/USA/CT-Yale-15438/2021	EPI_ISL_8563218	394	33.6	9.91×10^{04}	No	Not done	3	IDT V4
hCoV-19/USA/CT-Yale-15437/2021	EPI_ISL_8563217	401	26.1	9.77×10^{06}	Yes	Yes	3	IDT V4
hCoV-19/USA/CT-Yale-17291/2022	EPI_ISL_10815044	446	34.1	7.30×10^{04}	Not tested	Not done	2	IDT V4.1
hCoV-19/USA/CT-Yale-17881/2022	EPI_ISL_11025821	459	30.03	8.81×10^{05}	Not tested	Not done	2	IDT V4.1
hCoV-19/USA/CT-Yale-18086/2022	EPI_ISL_11503909	471	30.9	5.18×10^{05}	No	Not done	2	IDT V4.1

Table S2. Summary of sequenced SARS-CoV-2 genomes analyzed in this study collected from the infected immunocompromised patient. Related to Figures 2-6.

Sequence ID	Sequence data repository	Accession number
hCoV-19/USA/CT-Yale-12056/2021	GISAID	EPI_ISL_10548915
hCoV-19/USA/CT-Yale-12057/2021	GISAID	EPI_ISL_10548916
hCoV-19/USA/CT-Yale-12058/2021	GISAID	EPI_ISL_10548917
hCoV-19/USA/CT-Yale-12059/2021	GISAID	EPI_ISL_10548918
hCoV-19/USA/CT-Yale-12060/2021	GISAID	EPI_ISL_10548919
hCoV-19/USA/CT-Yale-4087/2021	GISAID	EPI_ISL_2035047
hCoV-19/USA/CT-Yale-12061/2021	GISAID	EPI_ISL_10548920
hCoV-19/USA/CT-Yale-12062/2021	GISAID	EPI_ISL_10548921
hCoV-19/USA/CT-Yale-5581/2021	GISAID	EPI_ISL_2716246
hCoV-19/USA/CT-Yale-5673/2021	GISAID	EPI_ISL_2776212
hCoV-19/USA/CT-Yale-5792/2021	GISAID	EPI_ISL_2860316
hCoV-19/USA/CT-Yale-12063/2021	GISAID	EPI_ISL_10548922
hCoV-19/USA/CT-Yale-6136/2021	GISAID	EPI_ISL_3133023
hCoV-19/USA/CT-Yale-6819/2021	GISAID	EPI_ISL_3370176
hCoV-19/USA/CT-Yale-9391/2021	GISAID	EPI_ISL_4198270
hCoV-19/USA/CT-Yale-9977/2021	GISAID	EPI_ISL_4576991
hCoV-19/USA/CT-Yale-10101R/2021	GISAID	EPI_ISL_10548912
hCoV-19/USA/CT-Yale-10960/2021	GISAID	EPI_ISL_10548913

hCoV-19/USA/CT-Yale-11558/2021	GISAID	EPI_ISL_5395558
hCoV-19/USA/CT-Yale-11887/2021	GISAID	EPI_ISL_5639913
hCoV-19/USA/CT-Yale-12124/2021	GISAID	EPI_ISL_5865553
hCoV-19/USA/CT-Yale-13443/2021	GISAID	EPI_ISL_7361483
hCoV-19/USA/CT-Yale-13444/2021	GISAID	EPI_ISL_7361527
hCoV-19/USA/CT-Yale-14026/2021	GISAID	EPI_ISL_7980711
hCoV-19/USA/CT-Yale-15439/2021	GISAID	EPI_ISL_8563219
hCoV-19/USA/CT-Yale-15438/2021	GISAID	EPI_ISL_8563218
hCoV-19/USA/CT-Yale-15437/2021	GISAID	EPI_ISL_8563217
hCoV-19/USA/CT-Yale-17291/2022	GISAID	EPI_ISL_10815044
hCoV-19/USA/CT-Yale-17881/2022	GISAID	EPI_ISL_11025821
hCoV-19/USA/CT-Yale-18086/2022	GISAID	EPI_ISL_11503909
hCoV-19/USA/CT-Yale-12056/2021	Sequence Read Archive	SRR23085675
hCoV-19/USA/CT-Yale-12057/2021	Sequence Read Archive	SRR23085674
hCoV-19/USA/CT-Yale-12058/2021	Sequence Read Archive	SRR23085663
hCoV-19/USA/CT-Yale-12059/2021	Sequence Read Archive	SRR23085652
hCoV-19/USA/CT-Yale-12060/2021	Sequence Read Archive	SRR23085651
hCoV-19/USA/CT-Yale-4087/2021	Sequence Read Archive	SRR23085650
hCoV-19/USA/CT-Yale-12061/2021	Sequence Read Archive	SRR23085649
hCoV-19/USA/CT-Yale-12062/2021	Sequence Read Archive	SRR23085648

hCoV-19/USA/CT-Yale-5581/2021	Sequence Read Archive	SRR23085647
hCoV-19/USA/CT-Yale-5673/2021	Sequence Read Archive	SRR23085646
hCoV-19/USA/CT-Yale-5792/2021	Sequence Read Archive	SRR23085673
hCoV-19/USA/CT-Yale-12063/2021	Sequence Read Archive	SRR23085672
hCoV-19/USA/CT-Yale-6136/2021	Sequence Read Archive	SRR23085671
hCoV-19/USA/CT-Yale-6819/2021	Sequence Read Archive	SRR23085670
hCoV-19/USA/CT-Yale-9391/2021	Sequence Read Archive	SRR23085669
hCoV-19/USA/CT-Yale-9977/2021	Sequence Read Archive	SRR23085668
hCoV-19/USA/CT-Yale-10101R/2021	Sequence Read Archive	SRR23085667
hCoV-19/USA/CT-Yale-10960/2021	Sequence Read Archive	SRR23085666
hCoV-19/USA/CT-Yale-11558/2021	Sequence Read Archive	SRR23085665
hCoV-19/USA/CT-Yale-11887/2021	Sequence Read Archive	SRR23085664
hCoV-19/USA/CT-Yale-12124/2021	Sequence Read Archive	SRR23085662
hCoV-19/USA/CT-Yale-13443/2021	Sequence Read Archive	SRR23085661
hCoV-19/USA/CT-Yale-13444/2021	Sequence Read Archive	SRR23085660
hCoV-19/USA/CT-Yale-14026/2021	Sequence Read Archive	SRR23085659
hCoV-19/USA/CT-Yale-15439/2021	Sequence Read Archive	SRR23085658
hCoV-19/USA/CT-Yale-15438/2021	Sequence Read Archive	SRR23085657
hCoV-19/USA/CT-Yale-15437/2021	Sequence Read Archive	SRR23085656
hCoV-19/USA/CT-Yale-17291/2022	Sequence Read Archive	SRR23085655

hCoV-19/USA/CT-Yale-17881/2022	Sequence Read Archive	SRR23085654
hCoV-19/USA/CT-Yale-18086/2022	Sequence Read Archive	SRR23085653

Table S3. Nucleotide substitution or mutation rates of the chronic infection samples and other SARS-CoV-2 variants. Related to Figure 3.

**Strain**	**Mutations or substitutions per year (s/y)**			**Mutations or substitutions per site year (s/s/y)**			**Regression R^2**
	**Estimate**	**Lower 95% CI**	**Upper 95% CI**	**Estimate**	**Lower 95% CI**	**Upper 95% CI**	
Chronic infection	35.55	31.56	39.54	1.21×10^{-03}	1.07×10^{-03}	1.34×10^{-03}	0.92
Theta	24.74	22.23	27.26	8.41×10^{-04}	7.56×10^{-04}	9.27×10^{-04}	0.67
All lineages	17.16	16.36	17.96	5.83×10^{-04}	5.56×10^{-04}	6.11×10^{-04}	0.41
B.1.517 (other)	16.93	13.46	20.41	5.76×10^{-04}	4.58×10^{-04}	6.94×10^{-04}	0.51
Lambda	15.30	13.96	16.64	5.20×10^{-04}	4.75×10^{-04}	5.66×10^{-04}	0.59
Omicron	12.26	9.68	14.84	4.17×10^{-04}	3.29×10^{-04}	5.05×10^{-04}	0.24
Mu	11.05	9.62	12.49	3.76×10^{-04}	3.27×10^{-04}	4.25×10^{-04}	0.35
Gamma	10.62	9.27	11.97	3.61×10^{-04}	3.15×10^{-04}	4.07×10^{-04}	0.34
Zeta	10.26	8.56	11.96	3.49×10^{-04}	2.91×10^{-04}	4.07×10^{-04}	0.27
Iota	9.31	7.45	11.17	3.17×10^{-04}	2.53×10^{-04}	3.80×10^{-04}	0.20
Epsilon	8.98	7.56	10.41	3.05×10^{-04}	2.57×10^{-04}	3.54×10^{-04}	0.30
Delta	8.96	7.64	10.27	3.05×10^{-04}	2.60×10^{-04}	3.49×10^{-04}	0.24
Beta	8.93	7.46	10.39	3.04×10^{-04}	2.54×10^{-04}	3.53×10^{-04}	0.27
Alpha	8.91	7.83	9.99	3.03×10^{-04}	2.66×10^{-04}	3.40×10^{-04}	0.29

Table S4. cDNA and forward primer sequences for Multiplexed Primer ID (MPID) MiSeq library preparation for the SARS-CoV-2 S gene and nsp12. Related to Figure 6.

**Mix A**		
**Region**	**cDNA primer with Primer ID**	**First Round PCR Forward**
S-N-3	GTGACTGGAGTTCAGACGTGTGCTCTTCCGATCTNNNNNNN<br>NNNNCAGTAGTACCAAAAATCCAGCCTCT	GCCTCCCTCGCGCCATCAGAGATGTGTATAAGAGACAGNNNN<br>AAGGGGTACTGCTGTTATGT
S-RBD-1	GTGACTGGAGTTCAGACGTGTGCTCTTCCGATCTNNNNNNN<br>NNNNCAGTAGTTGCTGATTCTCTTCCTGT	GCCTCCCTCGCGCCATCAGAGATGTGTATAAGAGACAGNNNN<br>AATTTAGTGCGTGATCTCCCT
S-RBD-10	GTGACTGGAGTTCAGACGTGTGCTCTTCCGATCTNNNNNNN<br>NNNNCAGTTGCTGGTGCATGTAGAAGTT	GCCTCCCTCGCGCCATCAGAGATGTGTATAAGAGACAGNNNN<br>TTCCGCATCATTTTCCACTTT
S2-9	GTGACTGGAGTTCAGACGTGTGCTCTTCCGATCTNNNNNNN<br>NNNNCAGTGGCAATGATGGATTGACTAGC	GCCTCCCTCGCGCCATCAGAGATGTGTATAAGAGACAGNNNN<br>GGCACAGGTGTTCTTACTGA
NSP12-5	GTGACTGGAGTTCAGACGTGTGCTCTTCCGATCTNNNNNNN<br>NNNNCAGTGTGCCAACCACCATAGAATTT	GCCTCCCTCGCGCCATCAGAGATGTGTATAAGAGACAGNNNN<br>CTTCTTCTTTGCTCAGGATGG
**Mix B**		
**Region**	**cDNA primer with Primer ID**	**First Round PCR Forward**
S1-15	GTGACTGGAGTTCAGACGTGTGCTCTTCCGATCTNNNNNNN<br>NNNNCAGTGTTCTAAAGCCGAAAAACCCT	GCCTCCCTCGCGCCATCAGAGATGTGTATAAGAGACAGNNNN<br>TTGATAACCCTGTCCTACCA
S-9	GTGACTGGAGTTCAGACGTGTGCTCTTCCGATCTNNNNNNN<br>NNNNCAGTAGCTATAACGCAGCCTGTAA	GCCTCCCTCGCGCCATCAGAGATGTGTATAAGAGACAGNNNN<br>TGTACGTTGAAATCCTTCACTG

S-gap-4	GTGACTGGAGTTCAGACGTGTGCTCTTCCGATCTNNNNNNNNNNNNCAGTCAGGGACTTCTGTGCAGTTA	GCCTCCCTCGCGCCATCAGAGATGTGTATAAGAGACAGNNNNCCGGTAGCACACCTTGTAAT
S2-3	GTGACTGGAGTTCAGACGTGTGCTCTTCCGATCTNNNNNNNNNNNNCAGTGACCTCTTGCTTGGTTTTGA	GCCTCCCTCGCGCCATCAGAGATGTGTATAAGAGACAGNNNNNTGCAGGTATATGCGCTAGTT

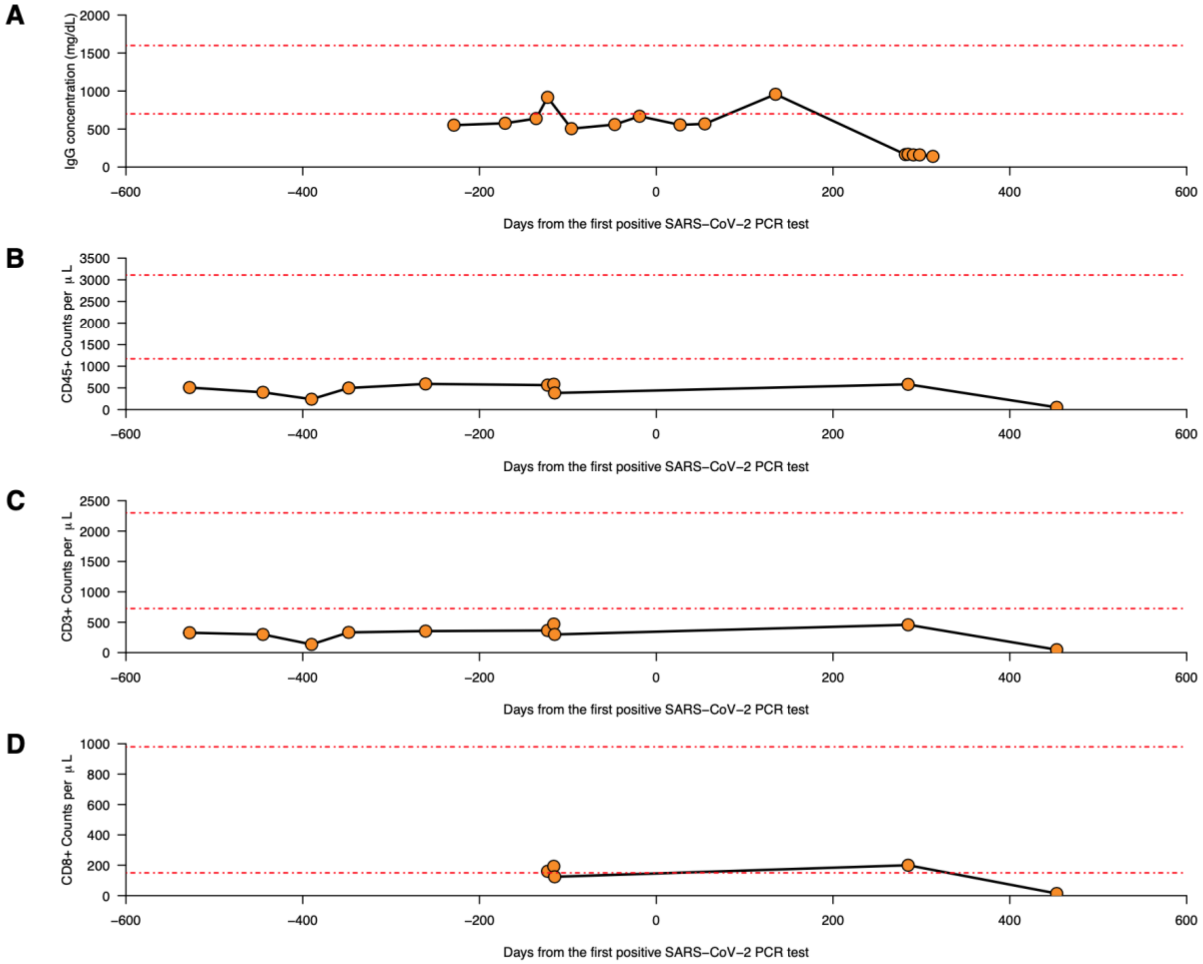

Figure S1: Key adaptive immune parameters as obtained from longitudinal chart reviews. Related to Figure 2. (A) The red dotted lines show the serum concentration of Immunoglobulin G (IgG) (reference range from 700-1600 mg/dL). **(B)** Cell counts per µl blood for $CD45^+$ lymphocytes (reference range from 1170-3110/µl are shown by the red dotted lines) **(C)**, $CD3^+$ T cells (reference range from 725-2300/µl are indicated by the red dotted lines) and **(D)** $CD8^+$ cytotoxic T cells (reference range from 150-980/µl are shown by the red dotted lines).

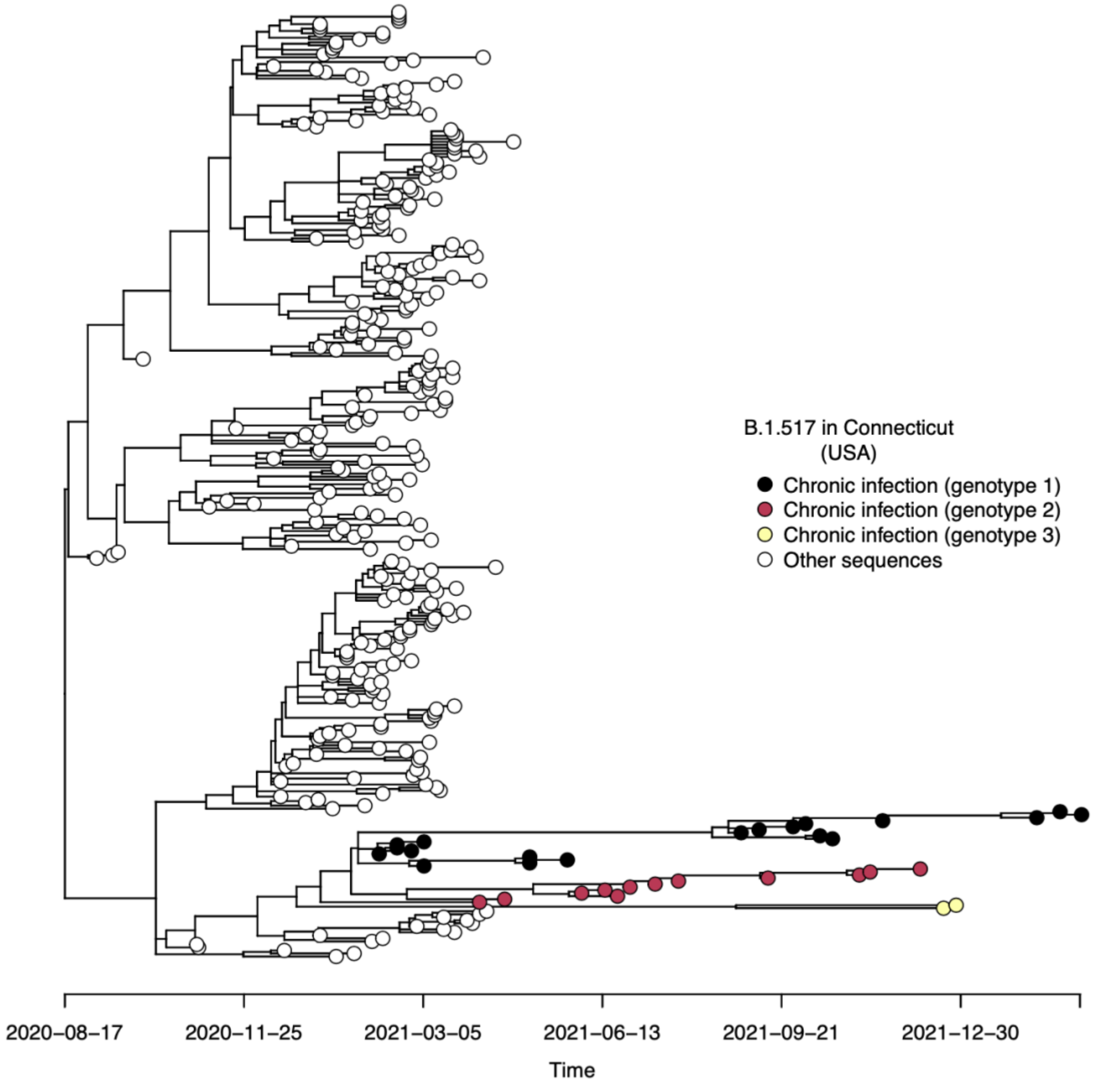

Figure S2: Time-resolved phylogenetic tree showing genetic relatedness of the B.1.517 SARS-CoV-2 strains from the chronic infection and contextual genomes from Connecticut, USA. Related to Figures 1 and 2. The B.1.517 sequences associated with the chronic infection formed a separate monophyletic clade from the rest of the B.1.517 sequences from Connecticut, USA indicating that there were no detectable onward transmission events from the patient with the chronic B.1.517 infection into the wider population in Connecticut, USA.

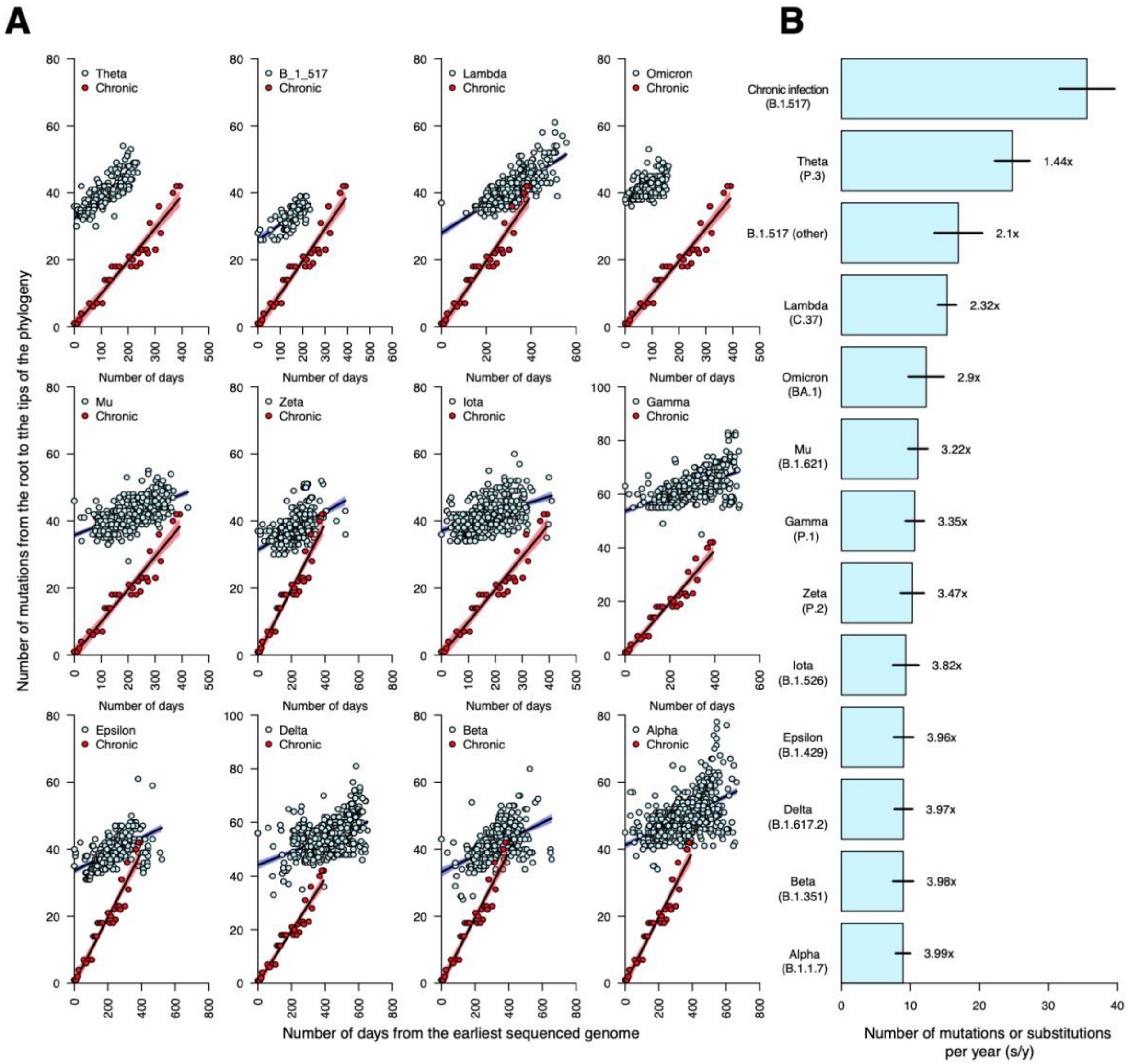

Figure S3: Genomes from B.1.517 chronic infection samples accelerated genetic divergence or higher mutation rates than other SAR-CoV-2 variants of interest and concern. **Related to Figure 3. (A)** Scatter plots show the relationship between phylogenetic root-to-tip distances, expressed as the number of mutations or nucleotide substitutions per site, and time as the number of days from the first sampled genome. The data points associated with the chronic infection are colored in red while those representing other variants are colored in sky blue. The lines and shaded bands surrounding them represent the linear regression models fitted to the data points for the chronic infection data and other variants. **(B)** Bar graph showing the average mutation rates expressed as the number of nucleotide substitutions per site per year (s/s/y) for the chronic infection samples and other variants based on the regression coefficients (β) generated from the plots in panel A. Specific values for the mutation rate are shown in **Table S3**.

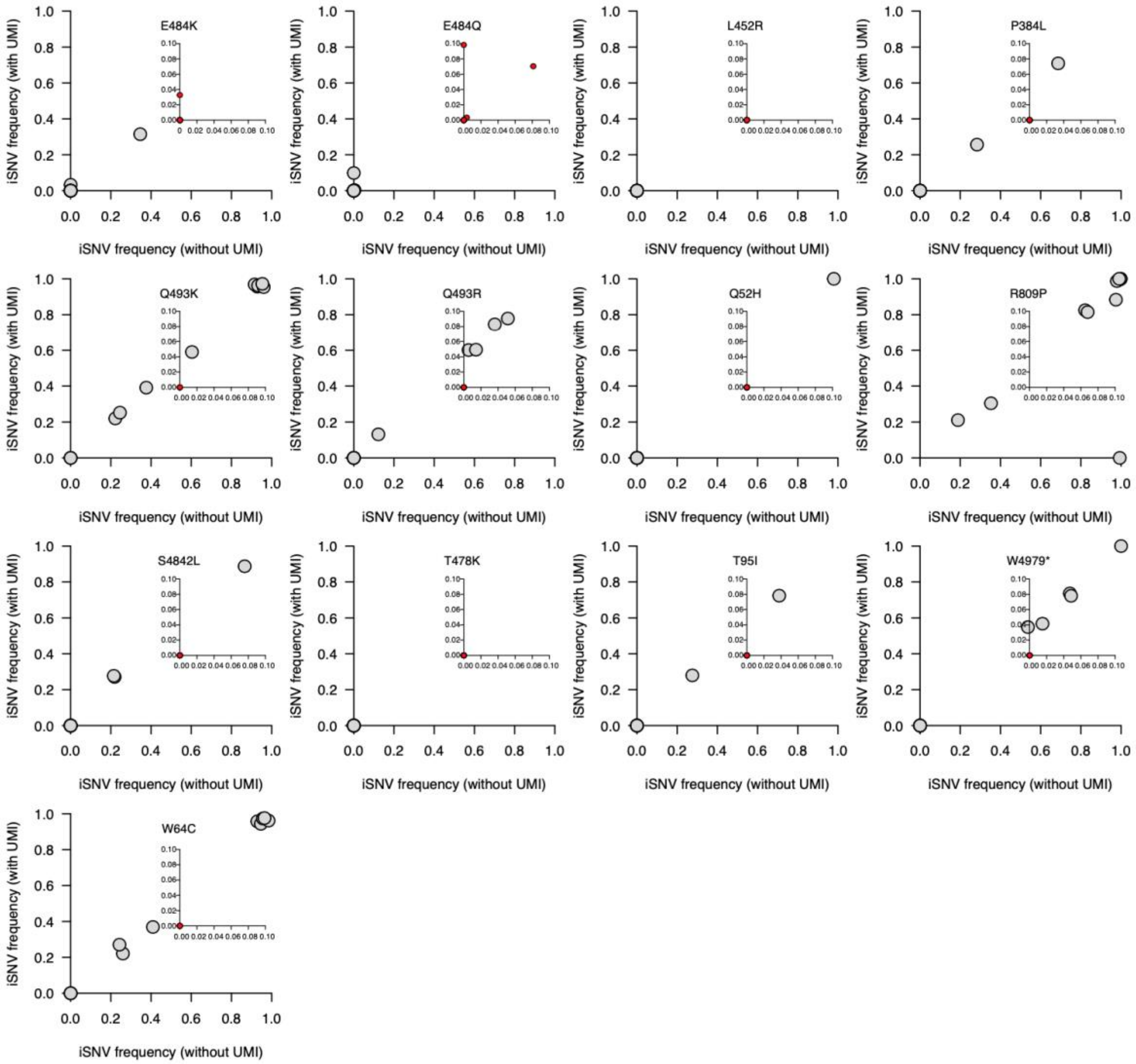

Figure S4: High concordance of the frequency of single nucleotide variants of the spike glycoprotein mutations between samples deep sequenced with and without unique molecular identifiers (UMI). Related to Figure 6. The genomic data without the UMIs were deep-sequenced using routine amplicon-based sequencing protocol using ARTIC V3, V4, and V4.1 primers for amplicon generation using 2×150 bp paired-end reads on an Illumina NovaSeq. Samples selected for deep-sequencing with the UMIs based on the Primer ID next-generation sequencing protocol were sequenced using MiSeq 2×300 bp paired-end reads. The insets show variants, colored in red, with frequencies between 0 to 0.1.

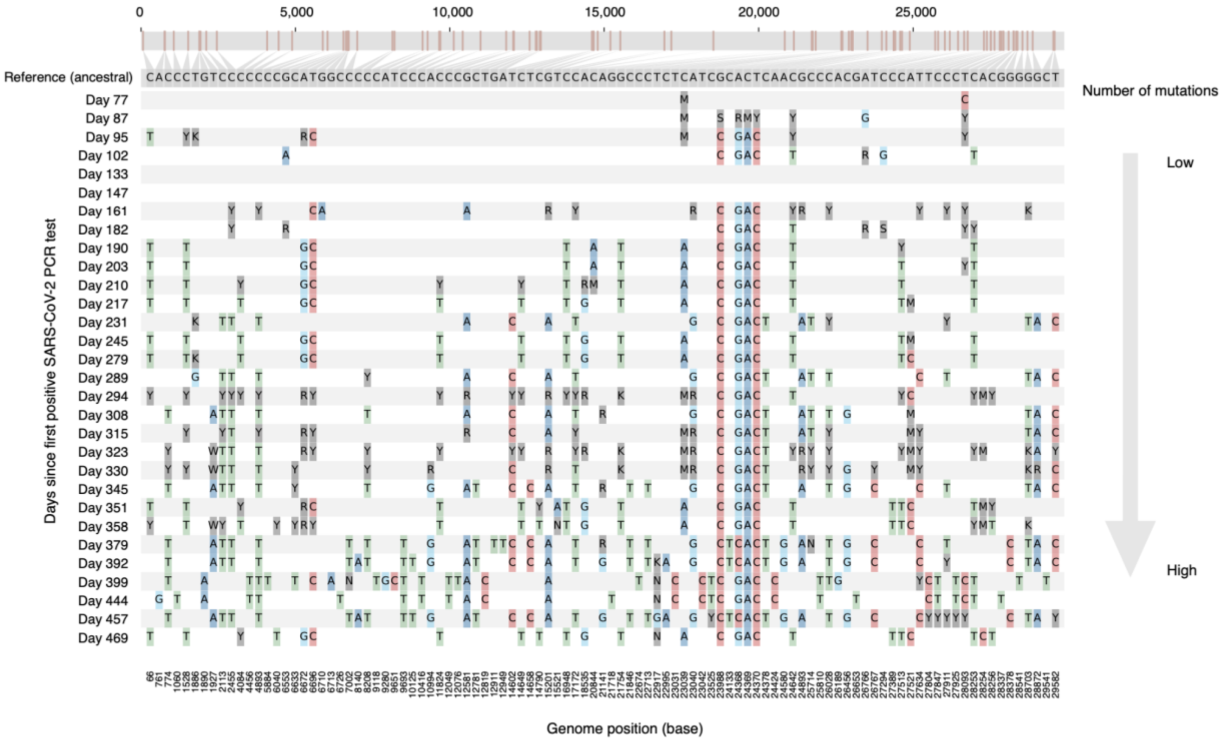

Figure S5: Nucleotide variation in B.1.517 SARS-CoV-2 genomes longitudinally sampled from a chronically infected patient. Related to Figures 4-5. Consensus sequences for each genome were compared against a reference genome, a reconstructed ancestral sequence for all the chronic infection genomes.

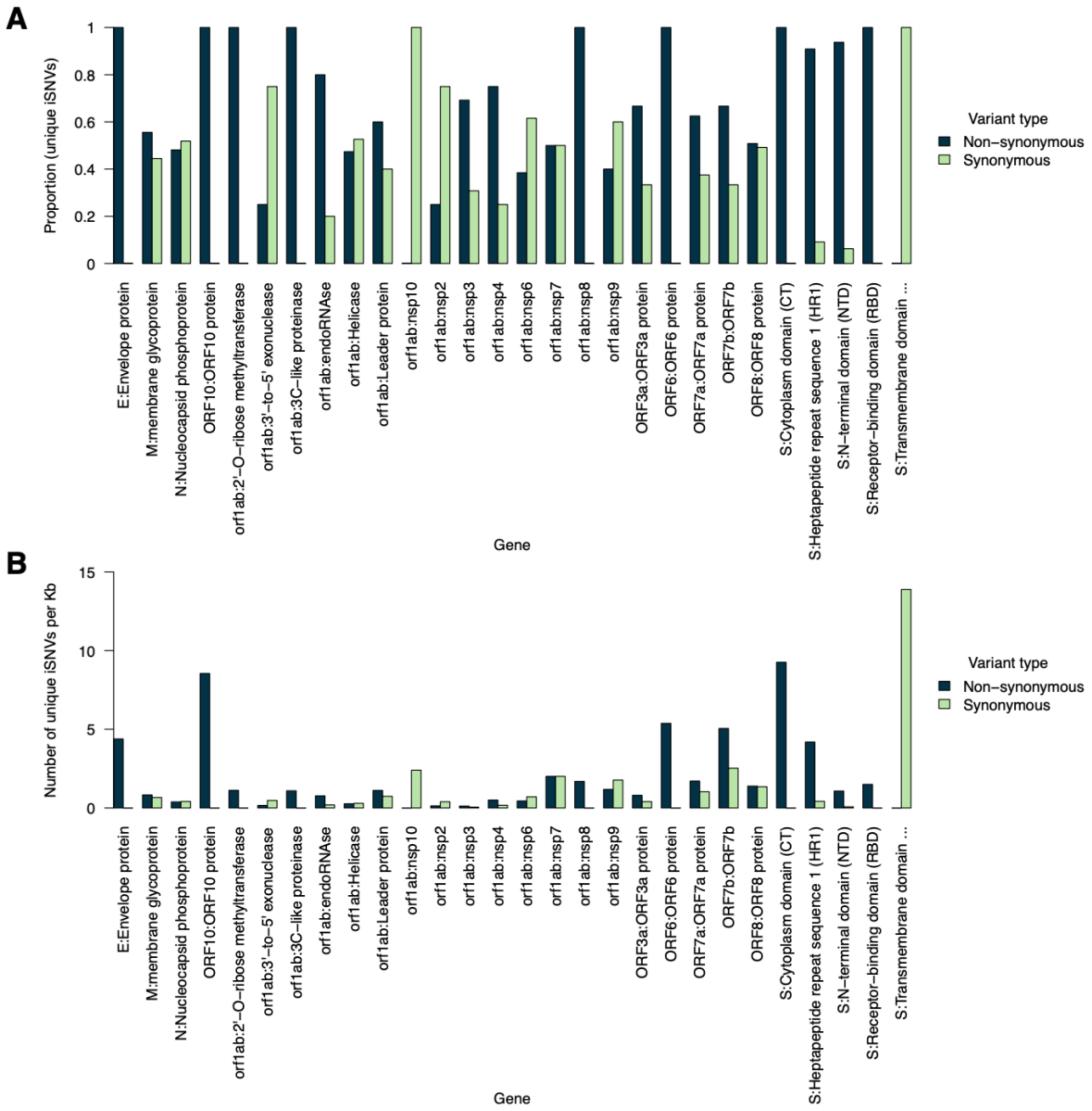

Figure S6: Distribution of iSNVs or mutations detected in the deep-sequenced longitudinal chronic infection samples. Related to Figures 4-5. The y-axis shows the number of iSNVs per kilobase for different SARS-CoV-2 genomic features based on the sequence annotations in the ancestral SARS-CoV-2 reference genome (GenBank accession: NC_045512.2). The bars in the graph are colored by the variant or mutation type. Additional information is provided in **Data S1**.

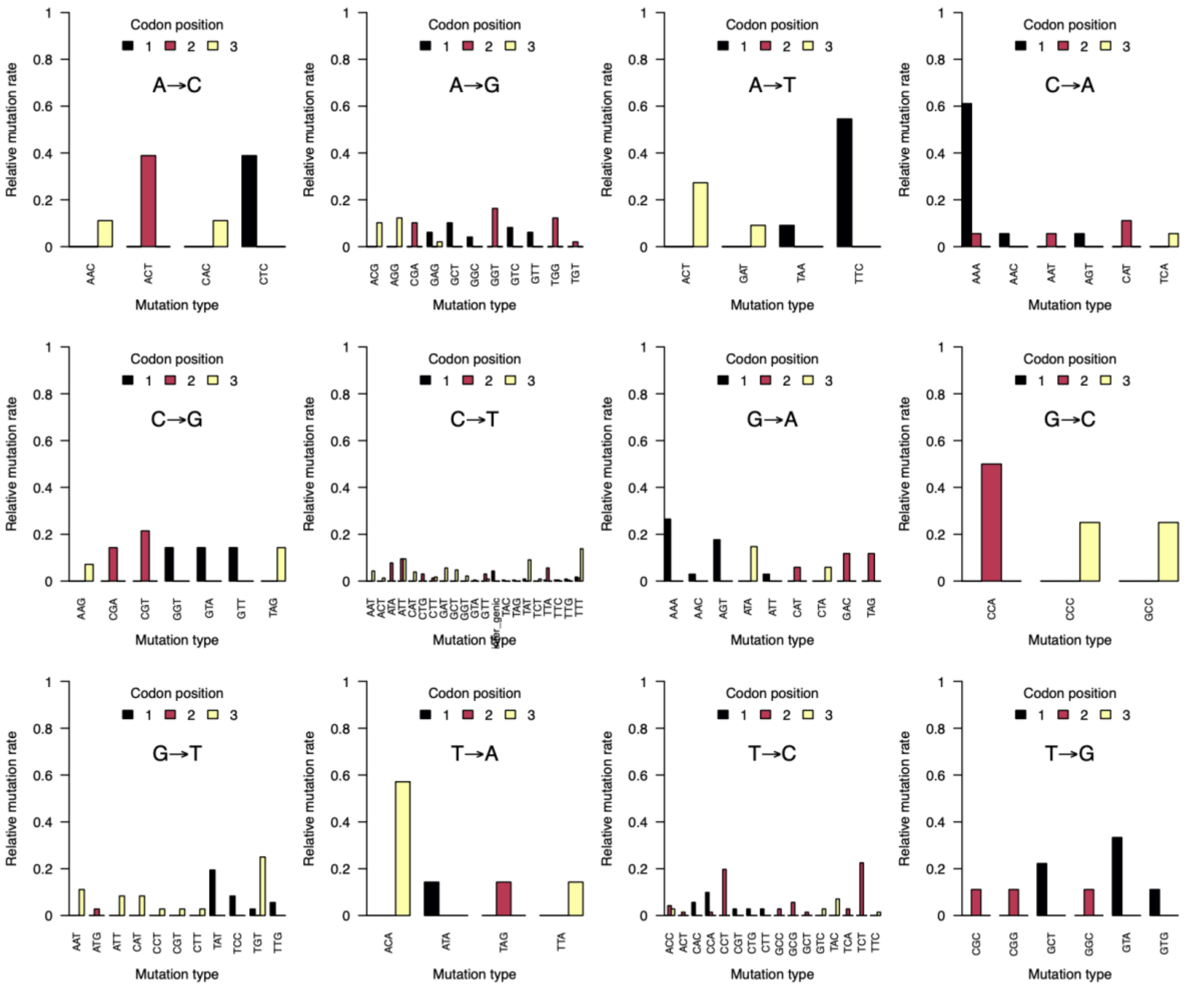

Figure S7: Mutation spectra of identified twelve trinucleotides during the B.1.517 chronic infection stratified by codon position. Related to Figures 4-5. Additional information is provided in **Data S1**.

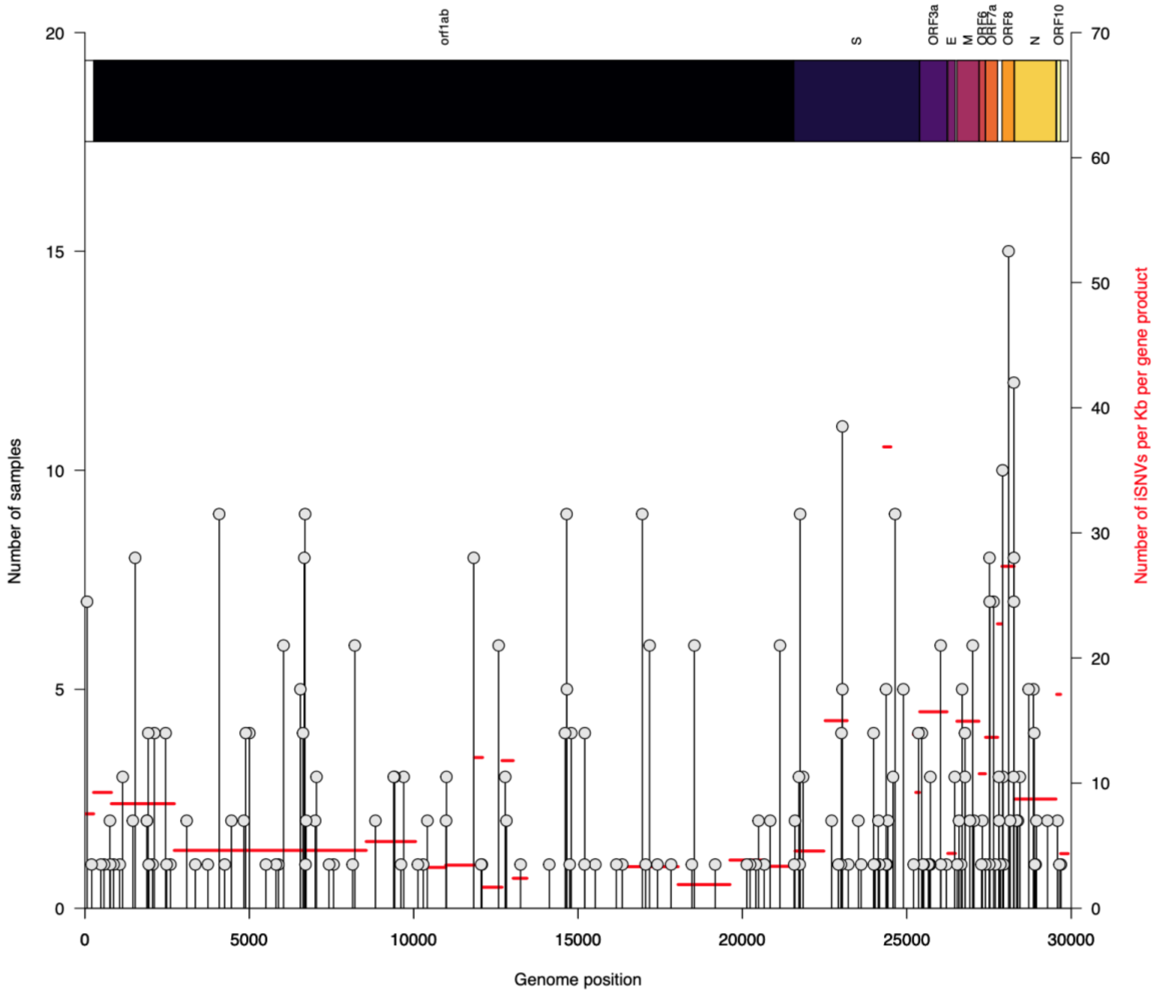

Figure S8: Distribution of iSNVs or mutations detected in the deep-sequenced longitudinal chronic infection samples. Related to Figures 4-5. Graph showing the number of samples containing each unique iSNV and its position in the ancestral SARS-CoV-2 reference genome. The y-axis labels on the right side of the plot show the number of iSNVs per gene and their position in the SARS-CoV-2 genomes stratified based on the sequence feature annotations in the ancestral SARS-CoV-2 reference genome (GenBank accession: NC_045512.2). Additional information is provided in **Data S1**.

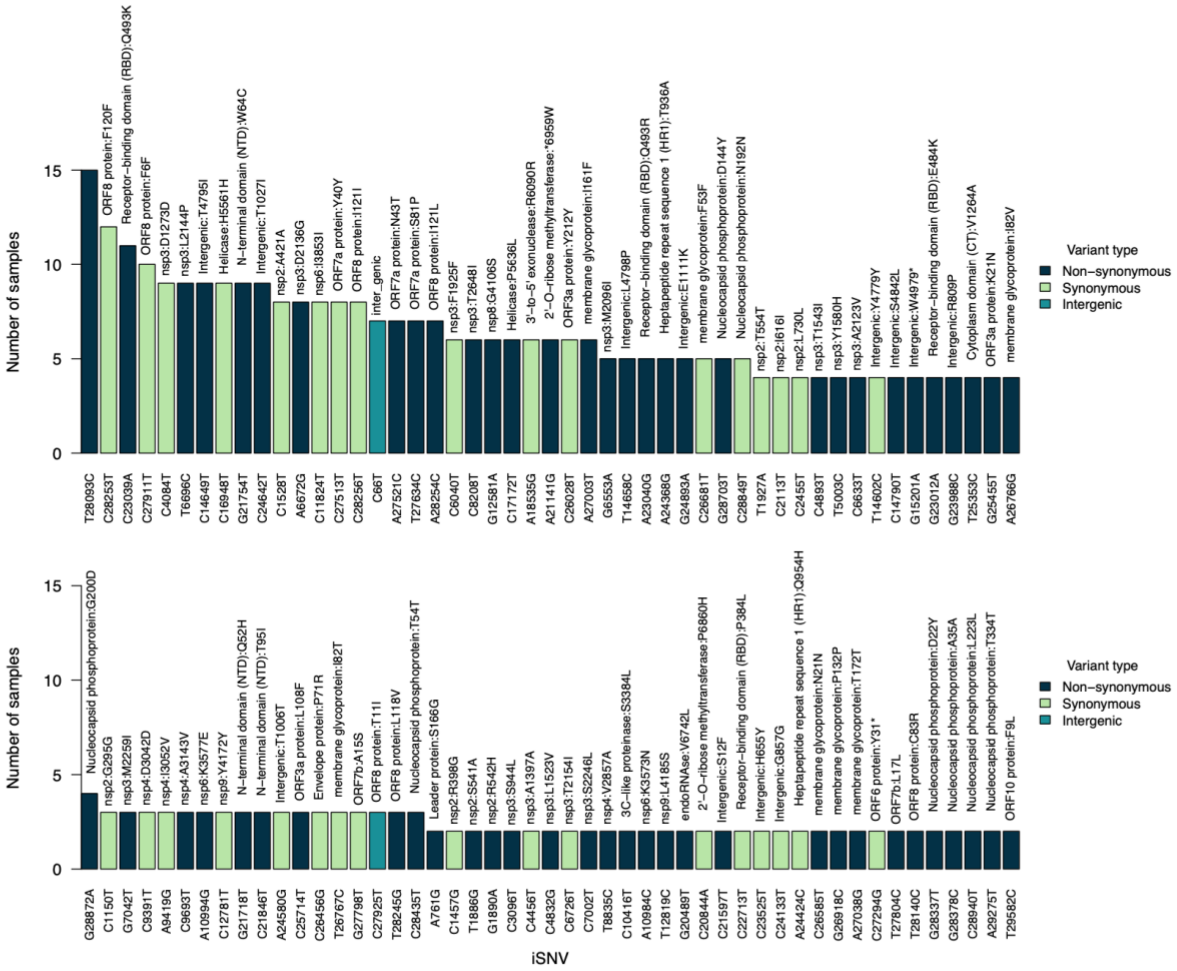

Figure S9: Distribution of iSNVs or mutations detected in the deep-sequenced longitudinal chronic infection samples. Related to Figures 4-5. The x-axis labels represent iSNVs corresponding to specific nucleotide substitutions and positions in the genome. The labels above the bars show the specific amino acid changes and their specific position in the SARS-CoV-2 genomes stratified based on the sequence feature annotations in the ancestral reference genome (GenBank accession: NC_045512.2). All the iSNVs are colored by the variant or mutation type. Additional information is provided in **Data S1**.

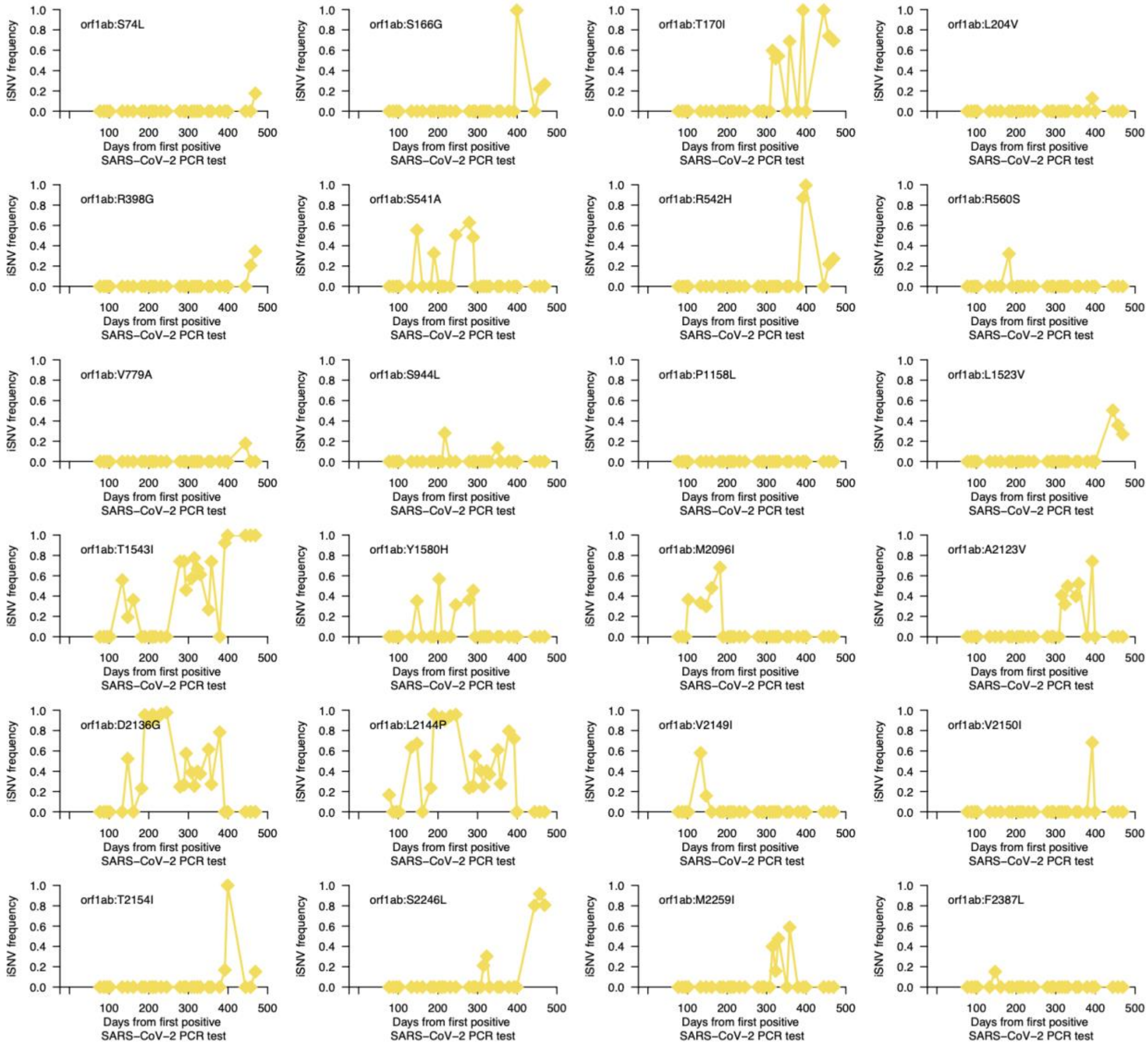

Figure S10: Intrahost non-synonymous mutation dynamics during chronic B.1.517 SARS-CoV-2 infection. Related to Figure 6. The graphs show temporal frequencies of non-synonymous iSNVs or mutations identified in the entire genome. Additional information for all the identified mutations (intergenic, synonymous, and non-synonymous) are provided in **Data S1**.

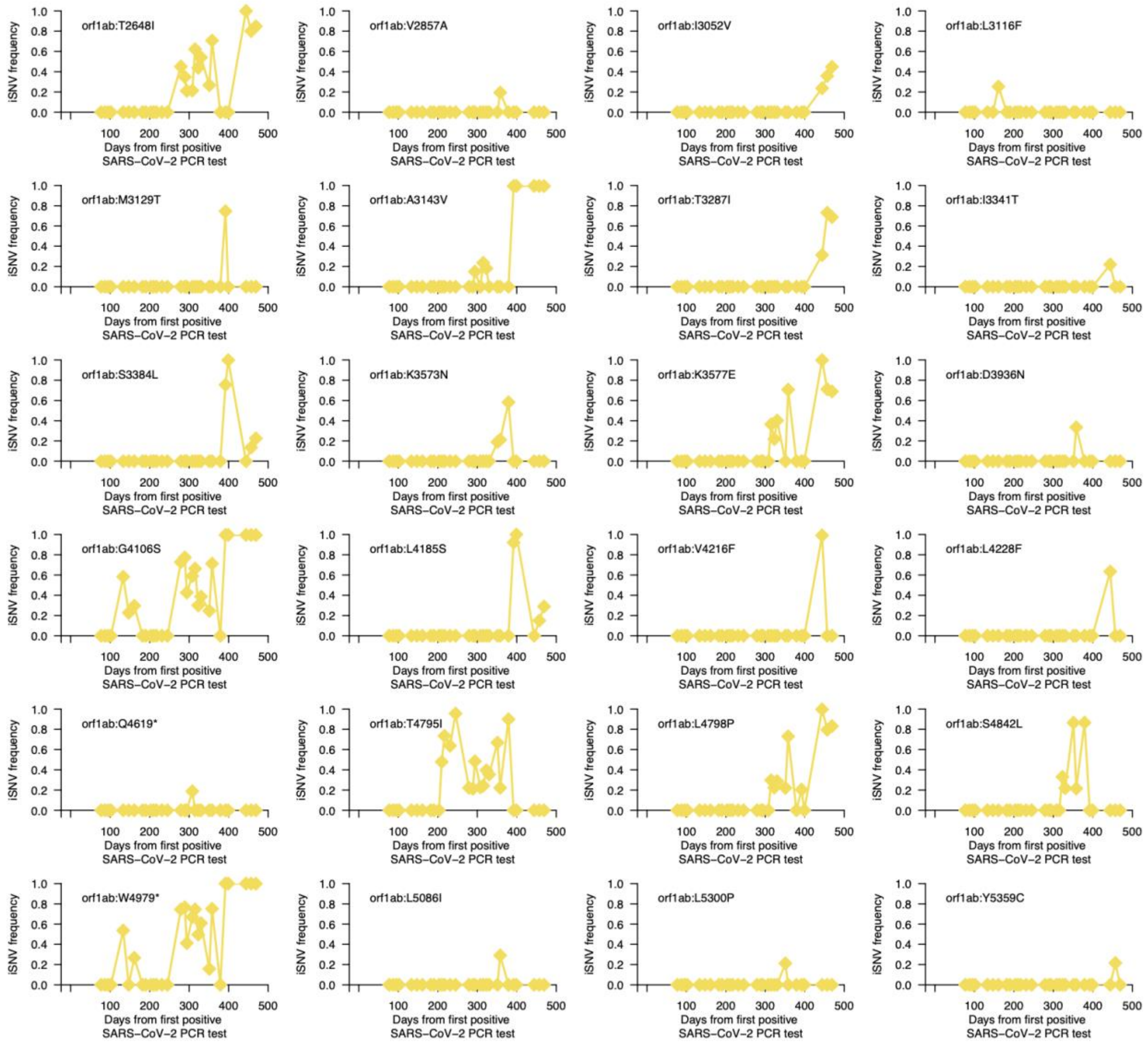

Figure S10 (continued): Intrahost non-synonymous mutation dynamics during chronic B.1.517 SARS-CoV-2 infection. Related to Figure 6. The graphs show temporal frequencies of non-synonymous iSNVs or mutations identified in the entire genome. Additional information for all the identified mutations (intergenic, synonymous, and non-synonymous) are provided in **Data S1**.

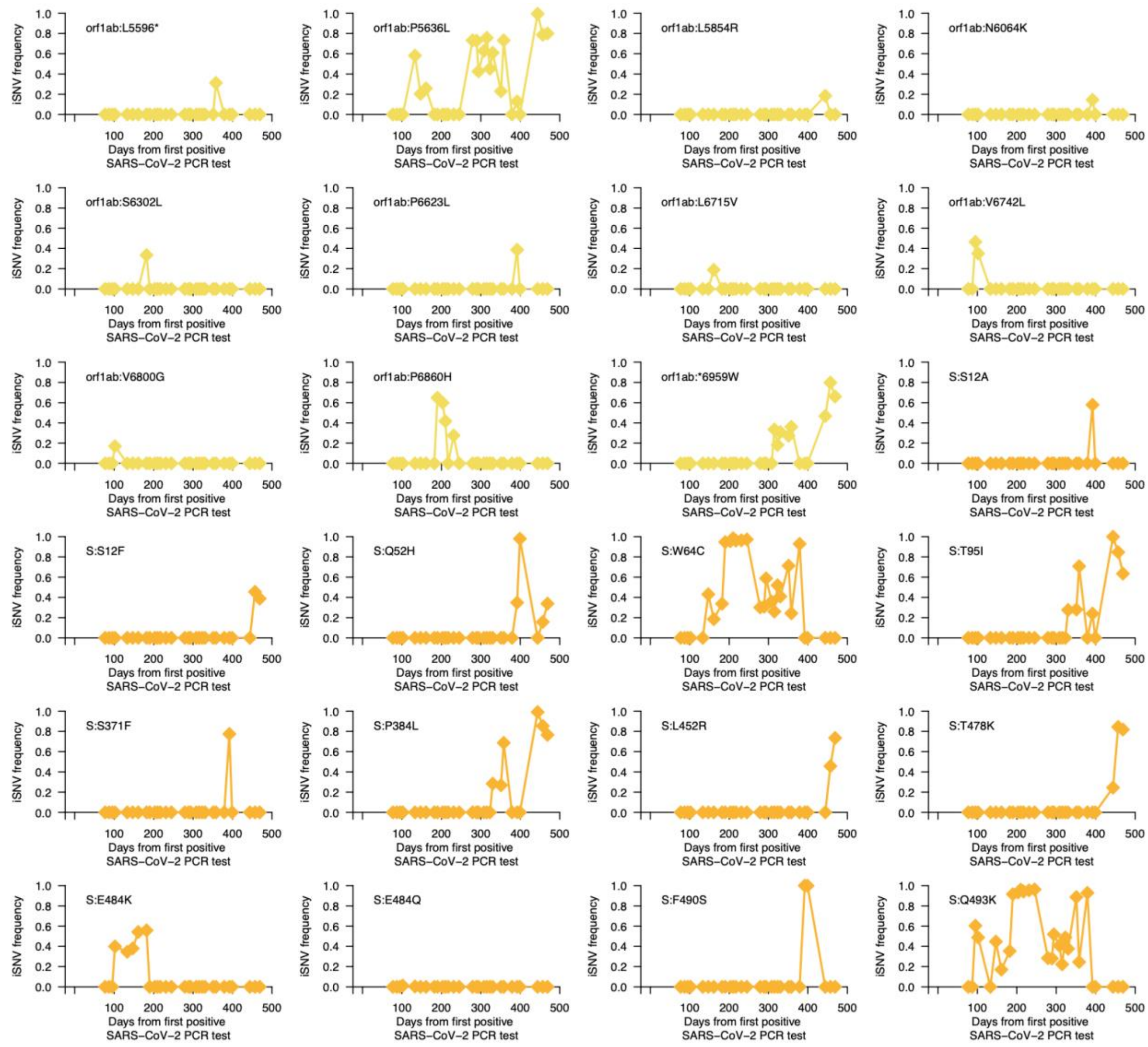

Figure S10 (continued): Intrahost non-synonymous mutation dynamics during chronic B.1.517 SARS-CoV-2 infection. Related to Figure 6. The graphs show temporal frequencies of non-synonymous iSNVs or mutations identified in the entire genome. Additional information for all the identified mutations (intergenic, synonymous, and non-synonymous) are provided in **Data S1**.

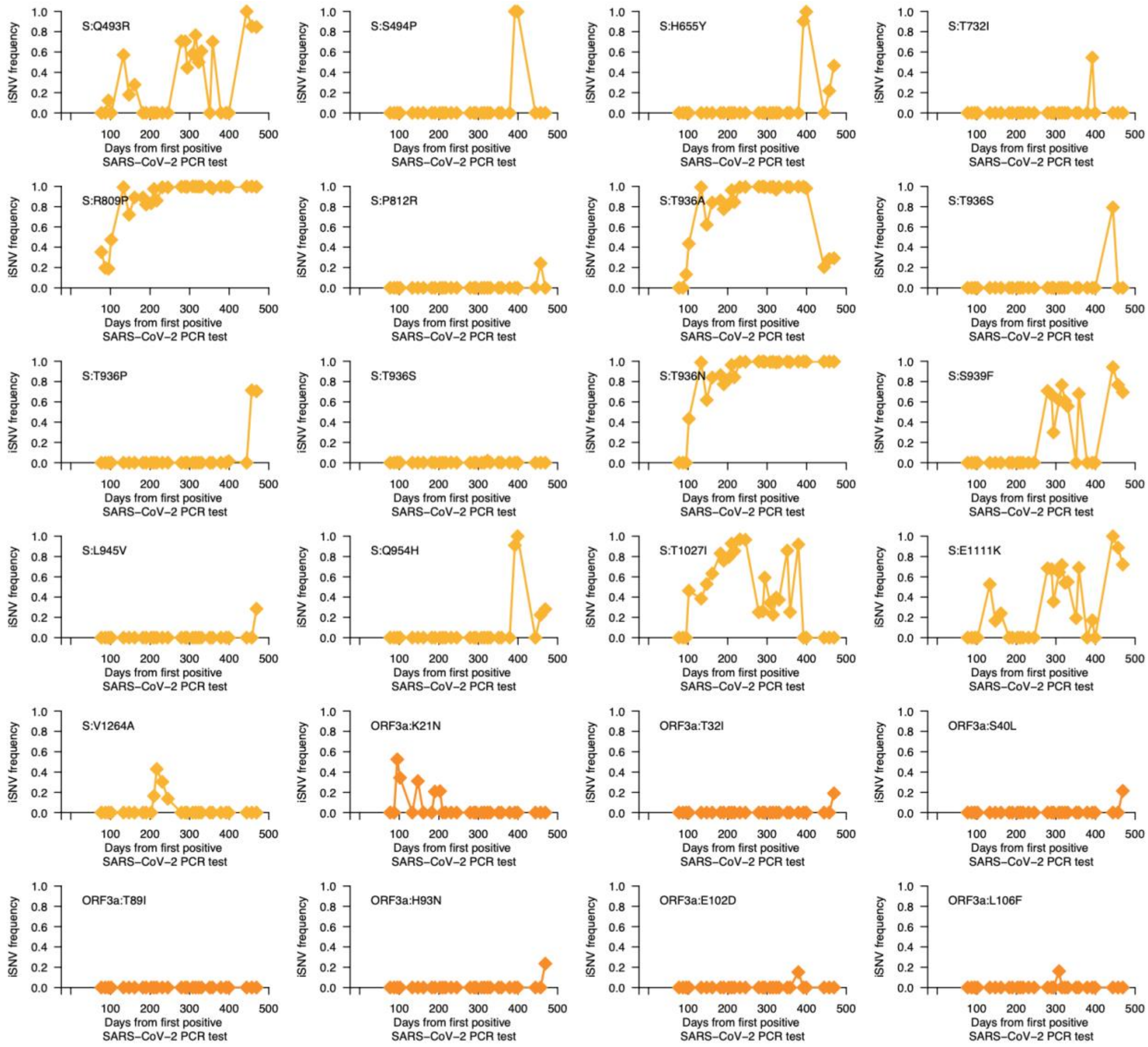

Figure S10 (continued): Intrahost non-synonymous mutation dynamics during chronic B.1.517 SARS-CoV-2 infection. Related to Figure 6. The graphs show temporal frequencies of non-synonymous iSNVs or mutations identified in the entire genome. Additional information for all the identified mutations (intergenic, synonymous, and non-synonymous) are provided in **Data S1**.

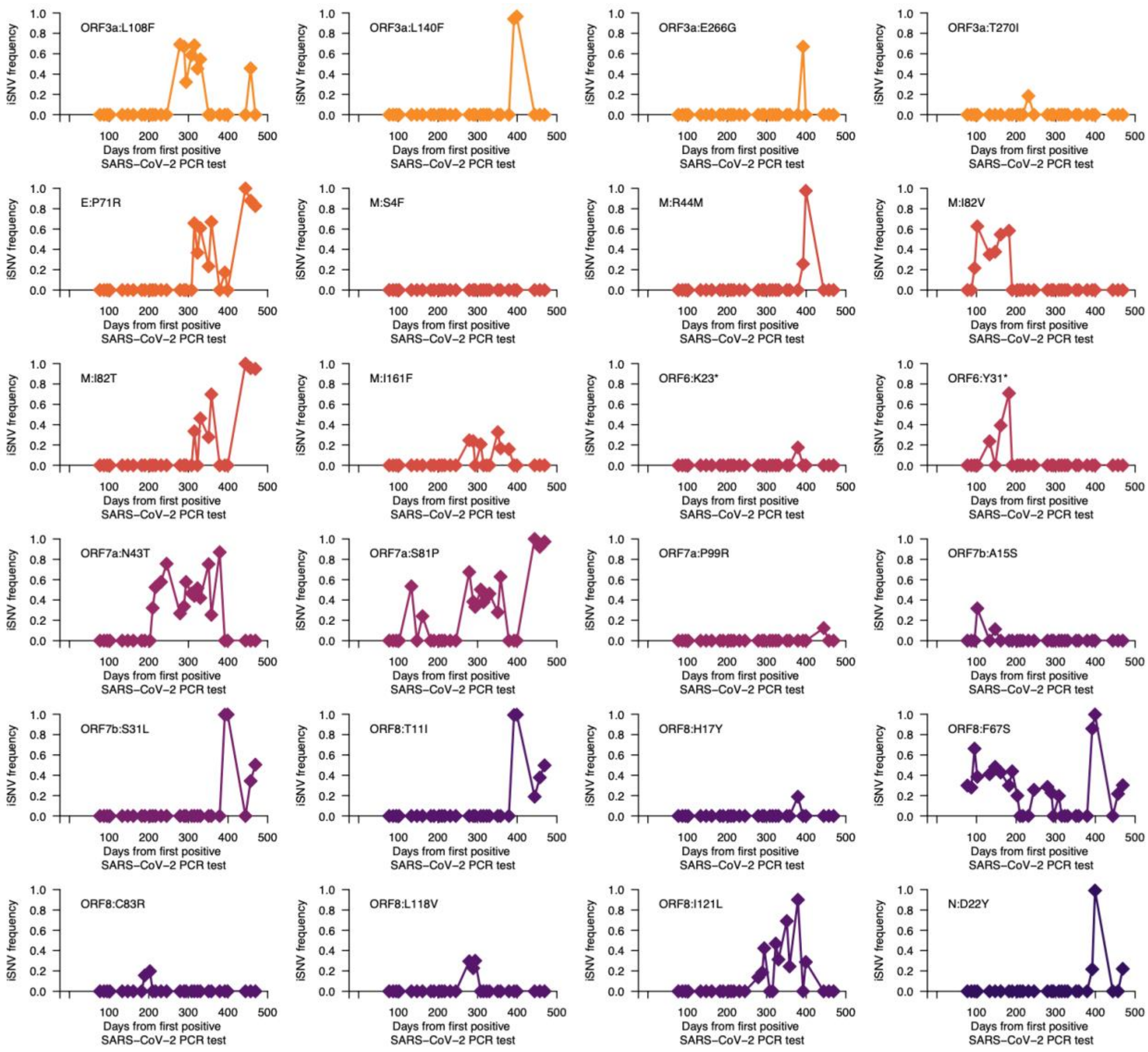

Figure S10 (continued): Intrahost non-synonymous mutation dynamics during chronic B.1.517 SARS-CoV-2 infection. Related to Figure 6. The graphs show temporal frequencies of non-synonymous iSNVs or mutations identified in the entire genome. Additional information for all the identified mutations (intergenic, synonymous, and non-synonymous) are provided in **Data S1**.

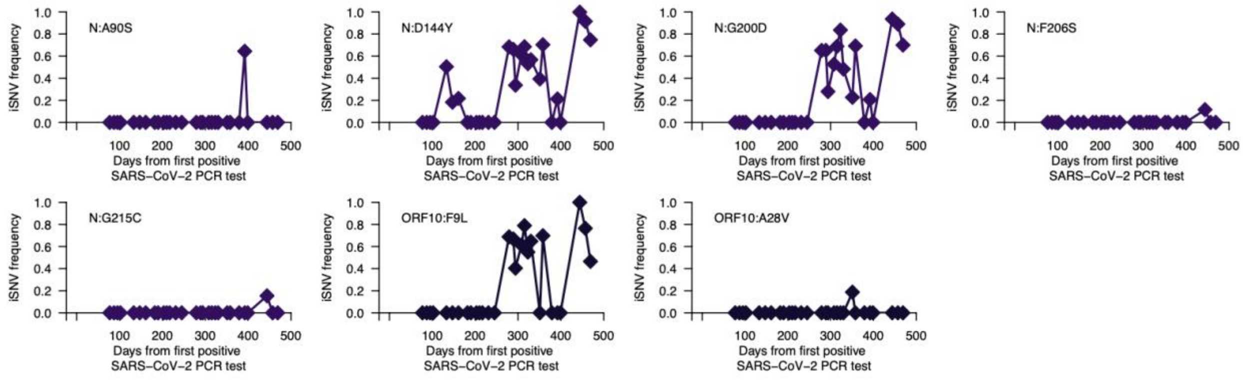

Figure S10 (continued): Intrahost non-synonymous mutation dynamics during chronic B.1.517 SARS-CoV-2 infection. Related to Figure 6. The graphs show temporal frequencies of non-synonymous iSNVs or mutations identified in the entire genome. Additional information for all the identified mutations (intergenic, synonymous, and non-synonymous) are provided in **Data S1**.
